# MRanalysis: a comprehensive online platform for integrated, multimethod Mendelian randomization and associated post-GWAS analyses

**DOI:** 10.1093/gigascience/giaf131

**Published:** 2025-10-22

**Authors:** Abao Xing, Tiantian Cai, Haofan Du, Zhifan Li, Hoiman Ng, Junrong Li, Guanmin Jiang, Lijun Chen, Kefeng Li

**Affiliations:** Centre for Artificial Intelligence Driven Drug Discovery, Faculty of Applied Sciences, Macao Polytechnic University, Rua de Luís Gonzaga Gomes, Macao 999078, Macao SR; Big Data and Internet of Things Program, Faculty of Applied Sciences, Macao Polytechnic University, Rua de Luís Gonzaga Gomes, Macao 999078, Macao SR; School of Physics and Technology, Nanjing Normal University, Nanjing, Jiangsu 210023, China; Big Data and Internet of Things Program, Faculty of Applied Sciences, Macao Polytechnic University, Rua de Luís Gonzaga Gomes, Macao 999078, Macao SR; Clinical laboratory, Kiang Wu Hospital 999078, Macao SR; Centre for Artificial Intelligence Driven Drug Discovery, Faculty of Applied Sciences, Macao Polytechnic University, Rua de Luís Gonzaga Gomes, Macao 999078, Macao SR; Department of Clinical Laboratory, The Fifth Affiliated Hospital, Sun Yat-sen University, Zhuhai, Guangdong 519000, China; Department of Hematology and Rheumatology, Zhuhai People’s Hospital (Zhuhai Hospital affiliated with Jinan University), Zhuhai 519000, China; Centre for Artificial Intelligence Driven Drug Discovery, Faculty of Applied Sciences, Macao Polytechnic University, Rua de Luís Gonzaga Gomes, Macao 999078, Macao SR

**Keywords:** Mendelian randomization, GWAS, online platform, MRanalysis, GWASkit, rs ID conversion, SNP-to-gene enrichment, visualization

## Abstract

**Background:**

Mendelian randomization (MR) is a powerful epidemiological method for inferring causal relationships between exposures and outcomes using genome-wide association study (GWAS) data. However, its adoption is limited by inconsistent data formats, lack of standardized workflows, and the need for programming expertise. To address these challenges, we developed MRanalysis, a user-friendly, web-based platform for integrated MR analysis, and GWASkit, a standalone tool for GWAS data preprocessing.

**Results:**

MRanalysis provides a comprehensive, no-code workflow for MR analysis, including data quality assessment, power estimation, single-nucleotide polymorphism to gene enrichment, and visualization. It supports univariable, multivariable, and mediation MR analyses through an intuitive interface. GWASkit facilitates rapid GWAS data preprocessing, such as rs ID conversion and format standardization, with significantly higher accuracy and efficiency than existing tools. Case studies demonstrate the utility and efficiency of both tools in real-world scenarios.

**Conclusions:**

MRanalysis and GWASkit lower barriers to MR analysis, making it more accessible, reliable, and efficient. By democratizing MR, these tools can accelerate discoveries in genetic epidemiology, inform public health strategies, and guide targeted interventions. MRanalysis is freely available at https://mranalysis.cn, and GWASkit can be accessed at https://github.com/Li-OmicsLab-MPU/GWASkit. Together, they represent a significant advance in understanding the complex relationships between genes, exposures, and health outcomes.

## Graphical Abstract

**Graphical Abstract:**
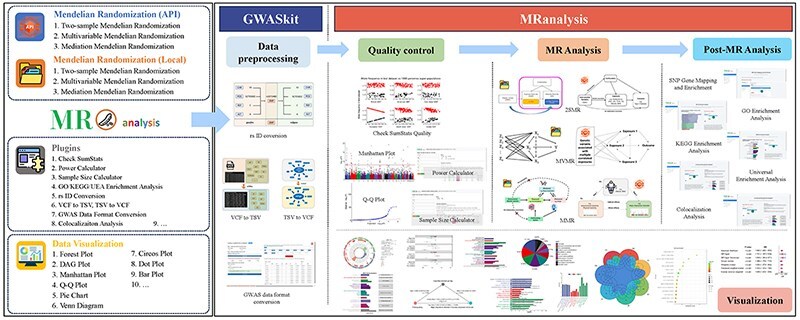

Key Points:

- GWASkit tool: A standalone, installation-free tool for rapid genome-wide association study (GWAS) dataset preprocessing and format standardization, outperforming current existing tools (e.g., ANNOVAR and gwaslab) in both single-nucleotide polymorphism rs ID conversion time and conversion accuracy.
- Versatility: Supports various Mendelian randomization (MR) methodologies, including univariable, multivariable, and mediation MR analyses, catering to diverse research needs.
- Efficiency and accuracy: Case studies demonstrate the utility, efficiency, and ease of use of both MRanalysis and GWASkit in real-world scenarios, highlighting their potential to accelerate MR research.
- Real-time code generation: Generates and assembles code based on user-defined parameters, enhancing transparency and reproducibility.
- Visual guidance: Detailed GIF tutorials for all applications, improving user experience.

## Introduction

Mendelian randomization (MR) is a powerful research approach that uses genetic variants (usually single-nucleotide polymorphisms [SNP]) as instrumental variables (IVs) to infer causal relationships between exposures and outcomes [1]. It is based on the stability of genes and Mendel’s first and second laws of inheritance [2]. The way genes are allocated determines that the relationship between genes and outcomes is not affected by postnatal environmental, behavioral, socioeconomic, and other confounding factors. Therefore, the causal relationships derived from MR studies are more reasonable and reliable. MR has evolved significantly since its inception. Early MR studies were generally limited by small sample sizes and involved few IVs, resulting in relatively low statistical power. However, with the exponential growth in the number of genome-wide association studies (GWASs) conducted globally, the summary data of tens of millions of relationships between exposures, diseases, and genetic variants have been successively released, continuously increasing the power of MR studies and significantly improving their accuracy.

Compared with traditional observational studies and randomized controlled trials (RCTs), MR studies have more advantages [3]. Observational studies are generally used to assess the causal relationship between exposures and outcomes [4], and RCTs also provide high-level evidence for causal relationship testing [5]. However, due to the need for strict quality control, comprehensive design, long-term follow-up, multieffect interventions, ethical issues, and compliance, observational studies or RCTs to elucidate disease outcomes are often not feasible. Observational studies or RCTs have difficulties in controlling all potential confounding factors, while MR studies can more effectively avoid these factors. Moreover, by utilizing the inherent characteristics of genetic variants, MR studies can address the common problem of reverse causality in observational studies and provide more reliable causal inference. For instance, MR suggests there is a causal relationship between gut microbiota and delirium [6]; Li et al. [7] used the MR method to study the association between cathepsins and lung cancer and indicate that elevated cathepsin H levels increase the overall risk of lung cancer, adenocarcinoma, and lung cancer among smokers; Ye et al. [8] performed a 2-sample MR analysis to estimate the causal effect of mental well-being, and some mediators were identified. In summary, MR has been instrumental in validating or refuting hypothesized causal relationships in various fields, including cardiovascular diseases [9–12], metabolic disorders [13, 14], psychiatric conditions [15–17], and drug discovery [18–20].

MR has emerged as a powerful approach for investigating causal relationships between exposures and outcomes using GWAS summary data. However, conducting MR analyses can be challenging due to the inconsistency of GWAS data format, the complexity of different methods, the lack of standardized workflows, and the need for extensive coding experience to complete the entire process, which can lead to unreliable results. Meanwhile, the lack of standardization of workflows not only affects the reliability of the results but also hinders the reproducibility of MR studies across different research groups. Existing tools for handling or visualizing GWAS datasets and conducting MR analysis are mostly implemented in R software and focus on specific functionalities, such as specific MR approaches, data munging, or plotting. The fragmentation of workflows further complicates the MR analysis process, as researchers must navigate multiple packages or tools and integrate them into a coherent workflow. These tools also often lack user-friendly interfaces, making them inaccessible to researchers without extensive programming skills. Furthermore, data preprocessing, a crucial and fundamental step in MR analysis, remains a significant hurdle, hindering the widespread adoption of MR in genetic epidemiology investigations. The lack of standardization in GWAS summary data formats across different databases or consortiums complicates the usage of these data. Despite efforts to develop a standard GWAS format, the large number of existing unprocessed GWAS summary data remains a challenge for data sharing and efficient reuse. It is therefore vital that we can ensure consistency across these datasets to minimize the risk of analytical mistakes due to user error. One such inconsistency is the naming of the effect allele and noneffect allele in these datasets [21]. Besides, the missingness of certain information (such as rs ID) and certain value conversions like −log10 transformation of *P* values can also hinder the direct reuse of these GWAS data, especially for beginners, and are error-prone during data and format conversions without careful reading manuals. Moreover, the conversion of SNP identifiers, specifically from CHR:POS:REF:ALT (chromosome, basepair location, noneffect allele, and effect allele) to rs IDs, is often time-consuming and inefficient with existing tools, and the need for strong programming skills to use command-line tools, coupled with the complex installation and deployment processes, further impedes the broader adoption of MR in genetic epidemiology studies.

To address these challenges in MR analyses and make them more accessible, reliable, and reproducible, we developed MRanalysis, an integrated, versatile, and comprehensive web-based platform for MR as well as some post-GWAS/MR analyses, and GWASkit, a standalone tool for rapid GWAS data preprocessing, to ensure seamless compatibility with a wide range of other post-GWAS tools and applications in our platform through functions such as rs ID conversion, data format standardization, and data extraction. Our aim was to create an intuitive, efficient, and user-friendly toolset that streamlines the entire MR workflow while enhancing accessibility for researchers across disciplines. By incorporating the guidelines proposed by Burgess et al. [22], we standardized MR analyses and ensured adherence to best practices, improving the quality and reproducibility of MR studies. The combination of these tools provides a comprehensive solution for handling and visualizing GWAS data, performing various MR analyses, and lowering the barrier to perform MR analyses. By streamlining the MR workflows, promoting best practices, and supporting various MR methodologies, including univariable, multivariable, and mediation MR analyses, our platform has the potential to accelerate MR studies and contribute to a better understanding of causal relationships in complex biological systems.

MRanalysis and GWASkit, as a zero-code platform and tool, meet the needs of researchers with limited programming experience, lowering the barrier to performing MR analyses. Through the powerful interactive capabilities of the MRanalysis platform, users can perform personalized analyses and visualizations. The platform also provides code generation functionality, assembling code based on the current user-set parameters. This offers further customization and result reproduction for users with some coding ability, allowing them to understand all the details of the entire analysis. Simultaneously, the powerful functionalities of GWASkit facilitate a wide range of post-GWAS analyses, greatly enhancing genetic research using GWAS summary data. To demonstrate the utility, practicality, convenience, and reproducibility of our platform and tool, we performed several real-world case studies. The development of MRanalysis and GWASkit represents a significant stride forward in genetic epidemiology research, facilitating more comprehensive investigations into relationships between genes and various phenotypes and accelerating discoveries in genetic epidemiology and drug discovery, ultimately leading to improved public health strategies and more targeted clinical interventions. Such a platform would serve as a bridge linking unprocessed GWAS summary data to post-GWAS/MR analysis tools seamlessly, enabling researchers with diverse backgrounds and varying levels of coding expertise to conduct MR studies with confidence. The increased accessibility and standardization of MR methods will not only enhance the reliability and reproducibility of MR studies but also foster collaboration and knowledge exchange among researchers from different disciplines.

## Materials and Methods

### Data sources

GWAS summary data regarding the data sources and sample sizes used in this study are outlined in Table 1. All cases utilized publicly available summary-level GWAS data from participants of European descent, and no specific ethical approval was necessary for conducting this study. The GWAS summary data for major depressive disorder (MDD), including genome-wide summary statistics from a meta-analysis of 33 cohorts of the Psychiatric Genomics Consortium (PGC) (excluding UK BioBank and 23andMe data), were described in Wray et al. [23]. The total number of individuals in these data is 500,199 (329,443 controls and 170,756 cases) with 8,483,301 variants analyzed. The frailty index (FI), derived from the cumulative defect model, served as a metric for assessing frailty severity [24] (sample size is 175,226). Each individual’s FI was calculated by dividing the number of defects by the total 49 defects. Individuals received a score of 0 or 1 based on the presence of defects (0 indicating none). A higher FI value indicated greater frailty. Other GWAS data were obtained from the Integrative Epidemiology Unit (IEU) OpenGWAS directly. For more detailed information, please refer to Table 1.

**Table 1:** GWAS data sources included in the case study

Phenotype	ID^a^	Sample size (overall or case/control)	Consortium or author	PMID
MDD	ieu-b-102	170,756/329,443	PGC, UK Biobank	30718901, Howard et al. [25]
Frailty index	ebi-a-GCST90020053	175,226	UK Biobank, TwinGene	34431594, Atkins et al. [24]
HDL cholesterol	ieu-a-299	187,167	GLGC	24097068, Willer et al. [26]
LDL cholesterol	ieu-a-300	173,082	GLGC	24097068, Willer et al. [26]
Triglyceride	ieu-a-302	177,861	GLGC	24097068, Willer et al. [26]
Creatinine	met-d-Creatinine	110,058	Borges CM	-
Coronary heart disease	ieu-a-7	60,801/123,504	CARDIoGRAMplusC4D	26343387, Nikpay et al. [27]
Feeling lonely	ebi-a-GCST006942	376,352	Mats N	29500382, Nagel et al. [28]
MDD in trauma-unexposed individuals	ebi-a-GCST009981	9,487/39,677	Jonathan R I C	31969693, Coleman et al. [29]
Cigarettes smoked per day	ieu-b-142	249,752	GSCAN	30643251, Liu et al. [30]

HDL: high-density lipoprotein; LDL: low-density lipoprotein; MDD: major depressive disorder; PGC: Psychiatric Genomics Consortium; PMID: PubMed ID.

^a^ID in IEU OpenGWAS.

### rs ID mapping

We evaluated the performance of 5 locally installed tools—GWASkit v1.0.0, ANNOVAR v2020-06-07 (latest version), snpEff v5.2c, MungeSumstats v1.10.1, and gwaslab v3.4.48—along with 2 web-based tools, the NCBI dbSNP database and SNPnexus, for their ability to handle rs ID conversion tasks. To ensure a comprehensive and concrete assessment, we utilized a large-scale GWAS data (GCST90236305 [31]) downloaded from the EMBL-EBI GWAS catalog, which contained 14,519,897 variants with complete information, including chromosome, basepair location, other allele (noneffect allele), effect allele, and rs ID, making it ideal test data. During the evaluation process, we maintained the default parameters and employed the latest default reference data for each of the 5 local tools to ensure a fair comparison.

The primary testing environment is in a Linux Ubuntu 20.04.4 long-term support (LTS) operating system, equipped with a 16-core Intel (CPU), 128 GB of RAM, and a 12 TB hard drive. To assess the cross-platform compatibility and the performance of our GWASkit, we additionally tested it on a Windows operating system featuring 16 GB of memory and Intel 4-core i7-8650U (CPU) with a 1 TB hard drive.

### GWAS summary data standardization

The GWAS summary data for MDD and FI were downloaded from DataShare and the GWAS catalog (above data sources session, Table 1), respectively. However, these 2 data were not in the standard variant call format (VCF) format, and the MDD GWAS data only contained rs ID without essential chromosome and position information. To address this issue, we utilized the GWASkit rs2pos (rs2pos -I PGC_UKB_depression_genome-wide.txt -O PGC_UKB_depression.tsv.gz –rs MarkerName –rsdb /rsdb/GRCh37 –SEP 1 –rm -V -Z) command to annotate the rs IDs with chromosome number, basepair position, and noneffect allele and effect allele information. Subsequently, we performed the tsv2vcf command (GWASkit tsv2vcf -I PGC_UKB_depression.tsv.gz -O PGC_UKB_depression.vcf.gz –TYPE GRCh37 –CHR CHR –POS POS –REF A2 –ALT A1 –RSID SNP –BETA LogOR –EAF Freq –SE StdErrLogOR –PVALUE P –SS 500199 -V) to standardize the GWAS statistics and generate the VCF file in a standard format, which can be directly uploaded to the MRanalysis online platform for performing MR analysis. In the case of the FI GWAS data, since it already contained all the necessary information for MR analysis, we only need to directly apply the tsv2vcf command for data standardization (GWASkit tsv2vcf -I 34431594-GCST90020053-EFO_0009885.h.tsv.gz –CHR chromosome –POS base_pair_location –REF other_allele –ALT effect_allele –RSID variant_id –BETA variant_id –EAF effect_allele_frequency –SE standard_error –PVALUE p_value –TYPE GRCh37 –SS 175226 -O GCST90020053.vcf.gz -V).

It is worth noting that all the abovementioned annotation and standardization operations can also be performed using the Windows version of GWASkit. The detailed process for using GWASkit on Windows can be found in the GWASkit help documentation, which is available on GitHub. By leveraging the powerful features of GWASkit, researchers can convert different kinds of formats of GWAS summary statistics into a standard one, enabling seamless integration with the MRanalysis platform for conducting MR analysis. This streamlined workflow not only saves time and effort but also ensures the accuracy and reliability of the results obtained from the MR analysis.

### GWAS summary data quality control

MRanalysis is also a convenient platform for processing the quality control (QC) of GWAS statistics, integrating 3 main functionalities: CheckSumStats, quantile–quantile (Q–Q) plot, and Manhattan plot.

CheckSumStats is an R package developed by Haycock et al. [32] that provides a QC pipeline to identify potential metadata errors, summary data issues, and other analytical problems in GWAS results. These errors and issues can introduce substantial bias into downstream analyses, such as 2-sample MR studies. CheckSumStats leverages 3 groups of SNPs to perform its check: a 1000 Genomes reference set, GWAS catalog associations, and the test GWAS top hits. By extracting summary data for these SNP groups from the target GWAS, CheckSumStats can confirm the identity of the effect allele frequency and effect allele columns, identify errors or analytical issues in the summary dataset, and infer the study’s ancestry. The package aims to enhance the integrity of collated summary data prior to analysis, thereby increasing the reliability of post-GWAS analyses. To make CheckSumStats more accessible to researchers, we developed a web-based application that allows users to directly upload their data for quality control analysis easily.

The Q–Q plot is a graphical representation of the deviation of the observed *P* values from the null hypothesis: the observed *P* values for each SNP are sorted from largest to smallest and plotted against expected values from a theoretical $\chi ^2$-distribution. Additionally, a Manhattan plot represents the *P* values of the entire GWAS on a genomic scale, and it is normally used to check for consistency and to identify spurious associations. In a Manhattan plot, the *P* values are represented in genomic order by chromosome and position on the chromosome (x-axis). The value on the y-axis represents the −log10 of the *P* value. In this case, we also use Q–Q plot and Manhattan plot applications in MRanalysis to perform these 2 analyses directly.

### Two-sample Mendelian randomization

**Figure 1:**
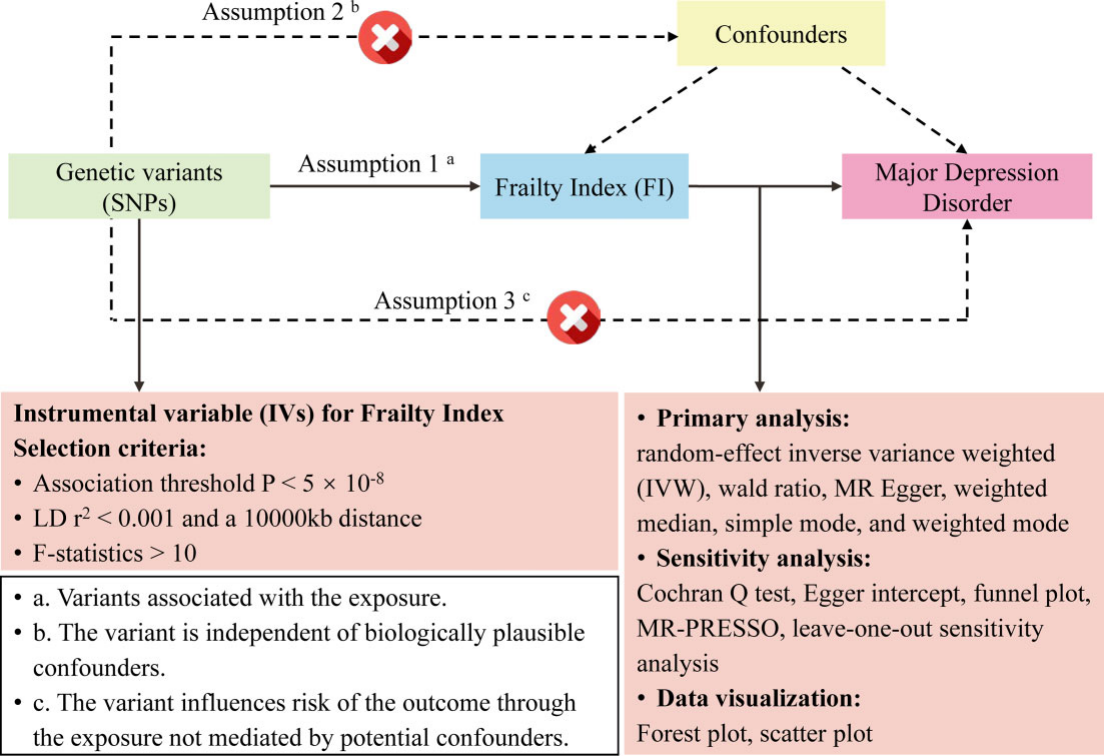
Workflow of the current 2-sample Mendelian randomization (MR) study revealing causality from the frailty index on major depressive disorder. LD: linkage disequilibrium; MR-PRESSO: MR pleiotropy residual sum and outlier; SNPs: single-nucleotide polymorphisms.

To validate the accuracy of our platform, we replicated the findings of causality between MDD and FI from Wang et al. [33] using our online platform. To investigate the causal relationship between MDD and FI, we obtained GWAS summary statistics for MDD (*n* = 500,199) and FI (*n* = 175,226) from previously published studies, serving as exposure and outcome datasets, respectively (Table 1). The SNPs associated with the exposures at genome-wide significance ($P < 5 \times 10^{-8}$) were selected as IVs with *F*-statistics all greater than 10, satisfying MR assumptions (Fig. 1). To ensure the independence of these IVs, we pruned them for linkage disequilibrium (LD) using LD clumping (*r*$^2$ < 0.001, distance = 10,000 kb).

In the MR analysis, we employed the random-effect inverse variance weighted (IVW) method as the primary analysis to study the causality between MDD and FI. We performed Cochran’s *Q* test to assess the heterogeneity. To evaluate the robustness of the MR estimates, we compared the IVW approach with other MR methods, including the Wald ratio, simple mode, MR-Egger, weighted median, and weighted mode. We also utilized the intercept term derived from MR-Egger and MR-PRESSO to assess the horizontal pleiotropy. Leave-one-out analysis was also conducted to evaluate the sensitivity of MR results.

All the aforementioned steps of 2-sample MR analysis can be performed online using our platform (both the application programming interface [API] and local modes) with default parameters. When removing confounding factors, we referred to the article by Wang et al. [33]. By leveraging our online platform, researchers can conveniently and without coding conduct 2-sample MR analyses to explore the causal relationships between complex traits and diseases, providing important theoretical foundations and practical guidance for the development of disease prevention and treatment strategies.

All MR analyses in our platform were conducted using “TwoSampleMR” (version 0.6.8) and “MendelianRandomization” (version 0.9.0) packages in R software (version 4.4.1).

### Multivariable Mendelian randomization

MR is a powerful approach for inferring causal relationships between exposures and outcomes using genetic variants (SNPs) as IVs. It can be conducted using either individual-level data or summary data from GWASs, which provide the estimated effect of each SNP of exposure on the outcome. Multivariable Mendelian randomization (MVMR) extends the traditional MR framework by allowing for the estimation of causal effects of multiple exposures on the outcome, conditional on the other exposures included in the model. MVMR also can be used to evaluate mediating effects of an independent variable, to adjust for possible pleiotropy bias due to horizontal pleiotropy of a specific effect, or to adjust for potential confounding.

**Figure 2:**
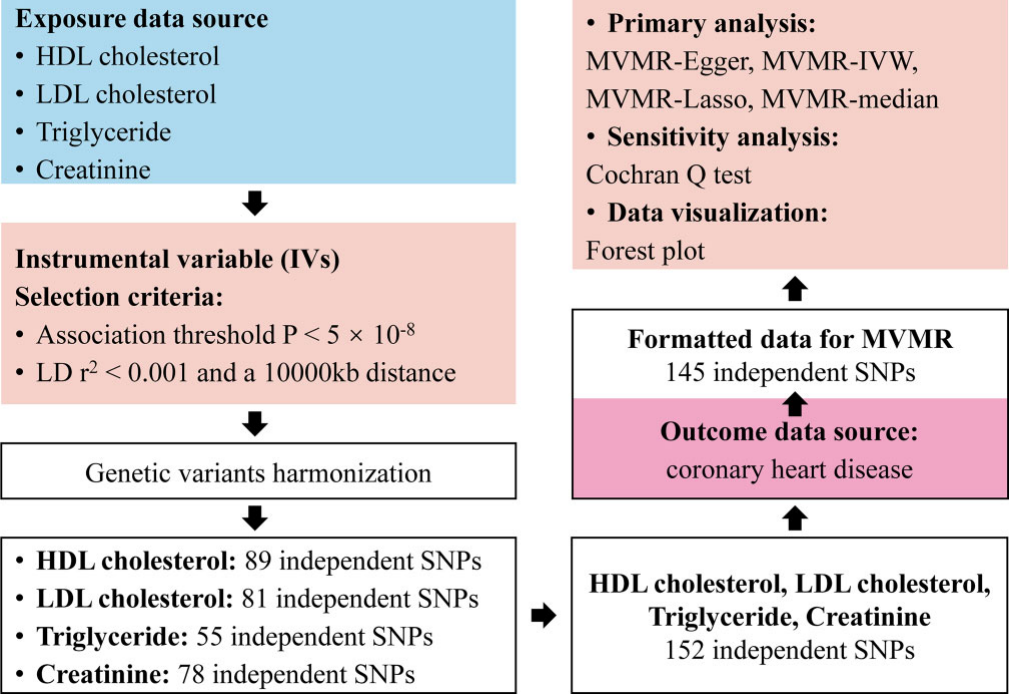
Workflow of the current multivariable Mendelian randomization (MVMR) study revealing causality from HDL cholesterol, LDL cholesterol, triglyceride, and creatinine on coronary heart disease. HDL: high-density lipoprotein; IVW, inverse variance weighted; LD: linkage disequilibrium; LDL, low-density lipoprotein; SNPs, single-nucleotide polymorphisms.

In this case, as shown in Fig. 2, we performed a 2-sample MVMR analysis using a summary dataset from GWAS of high-density lipoprotein (HDL) cholesterol, low-density lipoprotein (LDL) cholesterol, triglyceride, and creatinine as exposures and coronary heart disease (CHD) as the outcome (Table 1). We selected SNPs that reached genome-wide significance ($P < 5 \times 10^{-8}$) in at least one of the exposure traits and pruned them for LD using a pairwise *r*$^2$ threshold of 0.001 and 10,000 kb distance. The resulting set of independent SNPs was then used as IVs in the MVMR analysis. We employed several MVMR methods, including multivariable MR-Egger, multivariable IVW, multivariable MR-Lasso, and multivariable median-based approaches, to estimate the causal effects of the exposures on the outcome. These analyses were conducted using the “MVMR” (version 0.4) and “MendelianRandomization” packages in R.

To assess the strength and validity of the IVs in the 2-sample data, we used Cochran’s *Q* statistical test to evaluate the robustness of results and ensure that the assumptions of MR were satisfied. By leveraging the power of MVMR and the wealth of summary data from large-scale GWAS, our applications provide valuable insights into the complex causal relationships between multiple exposures and the outcome of interest. The use of multiple MVMR methods and the assessment of IVs’ strength and validity further strengthen the reliability of our tools.

### Two-step or mediation Mendelian randomization

Mediation analysis is a powerful approach to study the underlying mechanisms through which an exposure affects an outcome. In the context of MR, a 2-step MR analysis can be employed to assess the potential role of a third variable (mediator) in the causal pathway between exposure and outcome. In this application, the first step involves using genetic IVs associated with the exposure to determine the causal effect of the exposure on the potential mediator. The second step then utilizes IVs associated with the potential mediator, independent of those used in step 1, to estimate the effect of the mediator on the outcome of interest. Methods such as the product of coefficients can be applied to quantify the extent of mediation. Importantly, the MR assumptions must be satisfied for both steps of the analysis: (i) exposure on mediator and (ii) mediator on outcome.

**Figure 3:**
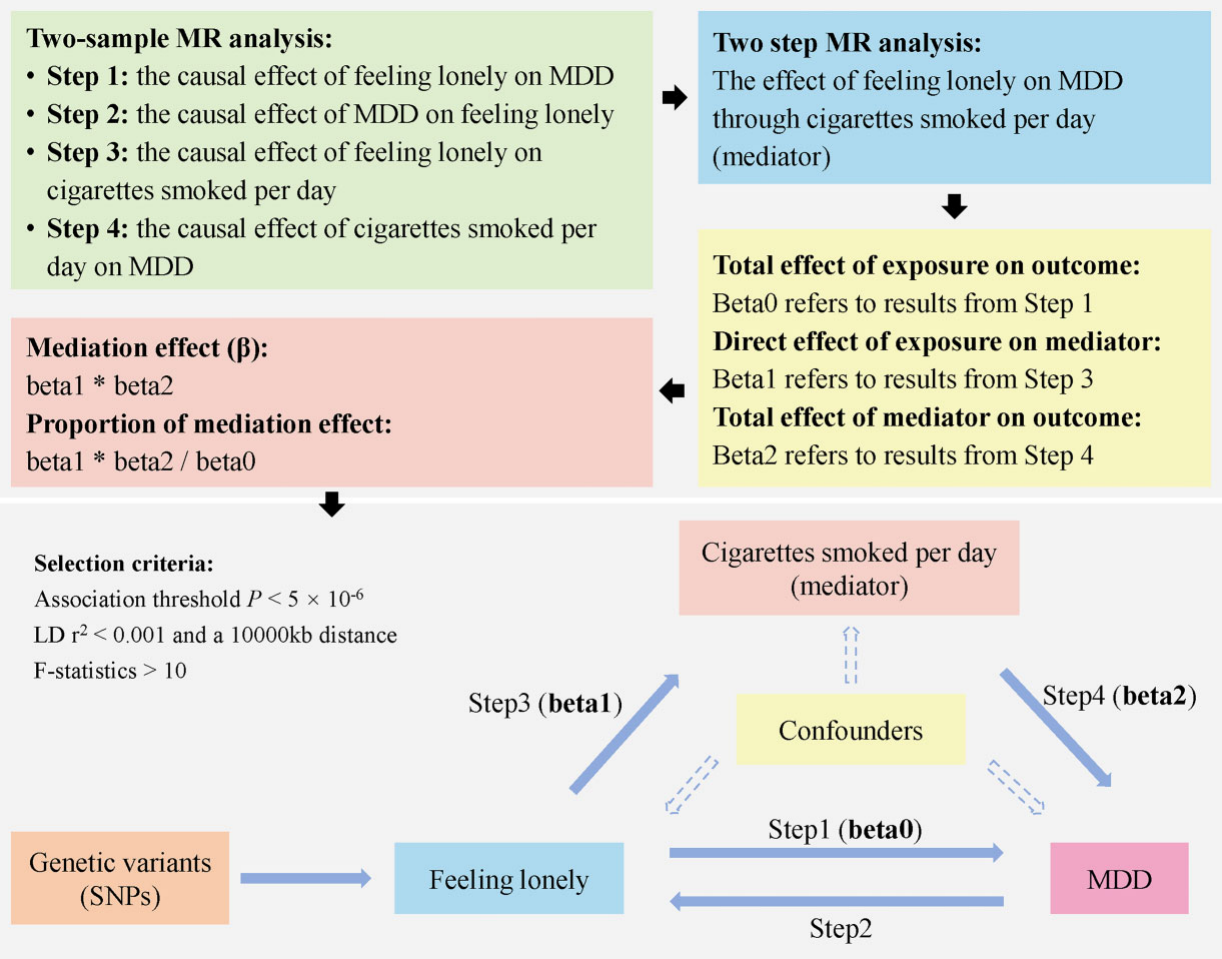
Flowchart of 2-step Mendelian randomization analysis revealing the mediating effect of cigarettes smoked per day on the risk of MDD through feeling lonely. MDD: major depressive disorder in trauma-unexposed individuals; MR: Mendelian randomization; SNPs: single-nucleotide polymorphisms.

In this case, we conducted a 4-step, 2-sample MR analysis to evaluate the complex relationships among feeling lonely, MDD in trauma-unexposed individuals, and cigarettes smoked per day (Table 1). Step 1 involved an MR analysis of feeling lonely on MDD, while step 2 examined the reverse causal relationship between feeling lonely and MDD. These 2 steps were combined into a bidirectional MR analysis to explore the potential primary and reverse causal relationships between feeling lonely and MDD. Steps 1, 3, and 4 were then integrated into a 2-step MR mediation analysis to assess the potential mediating role of cigarettes smoked per day in the relationship between feeling lonely and MDD. Specifically, step 3 investigated the causal effect of feeling lonely on cigarettes smoked per day, and step 4 evaluated the causal effect of cigarettes smoked per day on MDD. In this mediation analysis, beta0 represents the total effect, while beta1 and beta2 represent the direct effects of feeling lonely on cigarettes smoked per day and cigarettes smoked per day on MDD, respectively (Fig. 3). The specific calculation method (product of coefficients) for the mediating effect is also shown in Fig. 3.

In the first step, we evaluated the causal relationship between exposure and mediation variables (beta1). Subsequently, in the second step, we estimated the causal effect of mediators on outcomes through multivariate MR (beta2). We then calculated the total effect (beta0) between exposure and outcome using 2-sample MR analysis. When beta0, beta1, and beta2 were all significant, a causal relationship existed between the outcome and exposure, and the mediating variable played a partial mediational role in this causal relationship. The mediating effect was calculated using beta1*beta2, while the mediating proportion of the causal effect between exposure and outcome was calculated using (beta1*beta2)/beta0. Finally, we estimated the proportion of the mediation effect in the total effect using the delta method. We calculated the odds ratios (ORs) and 95% confidence intervals (CI) to measure causal effects.

### SNP gene mapping and enrichment

Gene and gene-set analysis are powerful statistical approaches that enable researchers to investigate the combined effects of multiple genetic markers on complex, polygenic traits. These methods are particularly useful when individual markers have weak effects that are difficult to detect using traditional single-marker analysis. Furthermore, gene-set analysis can provide valuable insights into the functional and biological mechanisms underlying the genetic component of a trait. While several methods for gene and gene-set analysis are available, they often suffer from various statistical issues and can be computationally intensive. To address these challenges, De Leeuw et al. [34] have developed a new method called MAGMA, which offers improved statistical power and computational efficiency compared to existing methods. To make MAGMA more accessible and user-friendly, we have developed a Shiny application that allows users to directly upload their data for this analysis. This application streamlines the process of conducting gene and gene-set analysis, making it easier for researchers to explore the genetic basis of complex traits.

The genes identified through the aforementioned mapping process were subsequently analyzed using our MRanalysis platform. The platform conducted Gene Ontology (GO) and Kyoto Encyclopedia of Genes and Genomes (KEGG) enrichment analyses based on the clusterProfiler (v4.12.6) R package [35], with default parameters applied.

### Power and sample size estimation

For power and sample size estimation, we used the PowerCalculator and SampleSizeCalculator applications integrated within the MRanalysis platform to determine statistical power and the minimum required sample size for 3 types of MR analyses: 2-sample univariable MR (2SMR), MVMR, and mediation MR (MMR). Because the tools do not directly support power and sample size calculations for MVMR and MMR, we decomposed each multivariable or mediation analysis into exposure–outcome components and estimated sample sizes separately. For each computation, only one of “sample size” or “power” was specified, and the other was computed by the tool. In case VIII, we applied the MR analyses described above and, for each exposure–outcome pair, computed the minimum required sample size individually. The minimum detectable effect size with the current samples, assuming 80% power and a 2-sided significance level of 0.05, was obtained using the SampleSizeCalculator application, which implements code from Burgess [36]. Required inputs included the proportion of variance in the exposure explained by the selected genetic instruments (*R*$^2$), the anticipated causal effect size (OR per standard deviation for binary outcomes or standard deviation change per standard deviation for continuous outcomes), the significance level ($\alpha$), and, for binary outcomes, the case-to-control ratio. For 2-sample analyses, the sample size was defined as the number of participants in the variant–outcome association dataset.

## Results

### Overall design and workflow of MRanalysis

MRanalysis is a comprehensive web-based platform designed to streamline and standardize MR analysis and preprocess the GWAS summary dataset. By leveraging the extensive interactive features of the R Shiny framework, MRanalysis provides a wide range of interactive functionalities, from handling various GWAS data file formats (e.g., comma-separated values (CSV), tab-separated values (TSV), VCF) to performing data extraction, format standardization, rs ID mapping, gene mapping, enrichment analysis, data visualization, and supporting several common MR approaches. To enhance user experience and facilitate easier adoption of our platform, we have prepared sample datasets for each application. These example datasets allow users to familiarize themselves with the platform’s functionalities and test its features before using their own data. Moreover, we have created comprehensive animated GIFs for each application, visually illustrating the key steps of the process. These animated guides provide a clear, step-by-step visual representation of how to navigate the platform and utilize its various tools. The overall structure of our platform can be divided into 3 main components: the analysis section (containing common MR approaches), the plugin section (encompassing various post-GWAS/MR methods), and the visualization section. The analysis section is the core component of MRanalysis, where researchers can conveniently perform univariable, multivariable, and mediation MR in both local and API modes.

**Figure 4:**
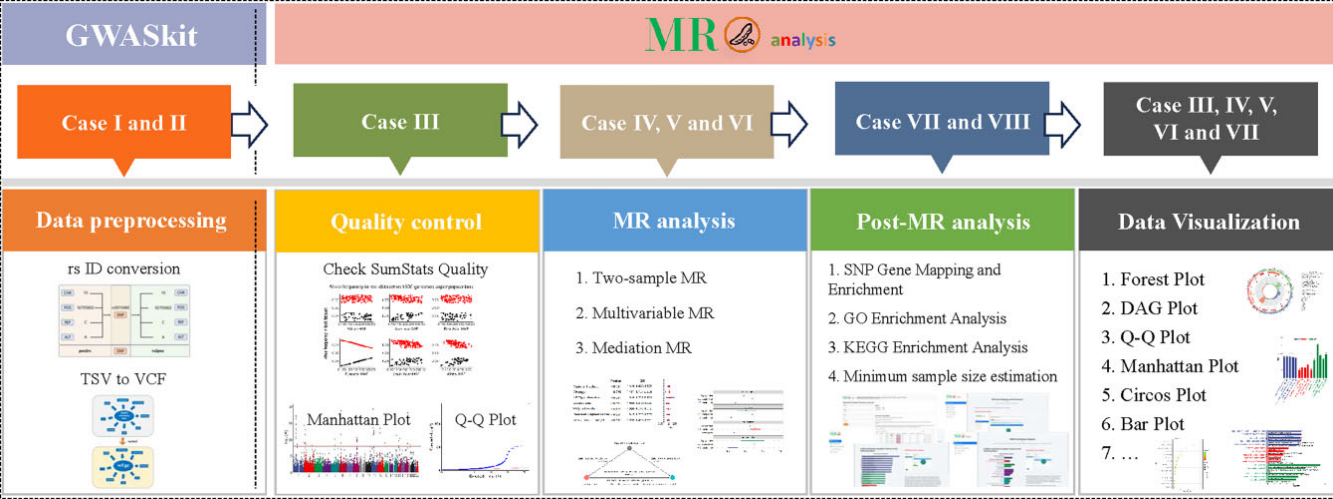
Overview of MRanalysis design.

When users want to perform an MR analysis, the overall pipeline can be divided into 5 main stages: data preprocessing, QC, MR analysis, post-MR analysis, and data visualization (Fig. 4). Taking local 2-sample MR analysis (univariable) as an example, (i) in the data preprocessing stage, users can utilize the GWASkit standalone tool to efficiently handle tasks such as format conversion (e.g., VCF to TSV or TSV to VCF), rs ID mapping (converting chromosome, basepair location, and effect and noneffect allele information to SNP IDs), and GWAS data format standardization. GWASkit’s “pos2rs” and “rs2pos” subcommands specifically cater to mapping between rs IDs and their coordinates, offering advantages of being preinstalled, multiplatform, efficient, highly accurate, and fast compared to other existing tools. (ii) The QC stage is crucial before conducting MR analyses. Our platform provides 3 key functions: CheckSumStats for identifying allele frequency conflicts and metadata errors in GWAS datasets, Manhattan plot for visualizing genetic associations and significance levels for multiple SNPs, and Q–Q plot for assessing the distribution and overall characteristics of GWAS data. These features ensure the quality and reliability of the data before proceeding with MR analyses. (iii) In the analysis stage, MRanalysis offers 3 mainstream MR approaches, including 2-sample univariable MR, multivariable MR, and mediation MR, through its “Analysis” module and associated plugins. Users can easily conduct these analyses using either API mode for searching GWAS data online or local mode for uploading their own GWAS data. (iv) After MR analysis, we can annotate instrumental variables and conduct gene enrichment analyses, including GO and KEGG. This process enhances biological interpretation of MR results, potentially revealing functional pathways underlying causal relationships. (v) Last, the results can be visualized using our plot applications, such as forest plot, directed acyclic graphs (DAGs), bar plot, dot plot, and circos plot, to facilitate intuitive interpretation and presentation of the findings.

To further enhance the robustness and reliability of MR analyses, MRanalysis also provides several additional utilities, including a Power Calculator application for estimating the statistical power of MR studies and a Sample Size Calculator application for determining the minimum sample size required for a given power [37]. The power of an MR study is determined by the sample size and the strength of the association between the proposed instruments and the risk factor [38]. Users can also perform SNP to gene annotation and enrichment analysis using the corresponding plugins (e.g., MAGMA application [34]) within our platform directly.

In summary, MRanalysis, coupled with the GWASkit, presents a complete and user-centric solution for researchers to efficiently handle GWAS data preprocessing, QC, MR analyses, and visualization, as well as perform enrichment analyses. The platform’s modular structure and extensive capabilities render it a crucial resource for researchers, particularly those with limited coding skills, to address the intricacies of MR analysis and elucidate causal relationships between genetic variants and phenotypes of interest. It is worth noting that each application within the platform provides complete code generation capabilities, enabling local reproduction of results. Through this code, users can clearly understand the entire analysis process and support further personalized analyses. The seamless integration of the 3 main sections within MRanalysis highlights its value and utility in MR, providing an accessible, versatile, and potent platform for scientists to overcome the challenges associated with GWAS data processing and MR studies.

### Case I: rs ID mapping

SNPs are genomic locations known to vary between individuals. The rs ID number is a unique identifier (“rs” followed by a number, e.g., rs12306) used by researchers and databases to designate a specific SNP. This naming convention, which stands for Reference SNP cluster ID, is widely used for most SNPs. When researchers identify a SNP, they send a report containing the sequence surrounding the SNP to the dbSNP database. Submitted variants are categorized, organized, and annotated, with duplicate variants being consolidated. Unlike the CHR-POS identifier, which changes with different reference genome versions, rs ID remains consistent across versions. This consistency provides a stable method for representing variants, making it more suitable for large-scale studies in population genetics or precision medicine.

GWASkit pos2rs provides functions to convert CHR-POS-REF-ALT (chromosome, basepair locations, noneffect [other or reference] allele, and effect allele) to rs ID using reference files downloaded from the National Center for Biotechnology Information (NCBI) dbSNP database or self-prepared files for rapid conversion.

**Figure 5:**
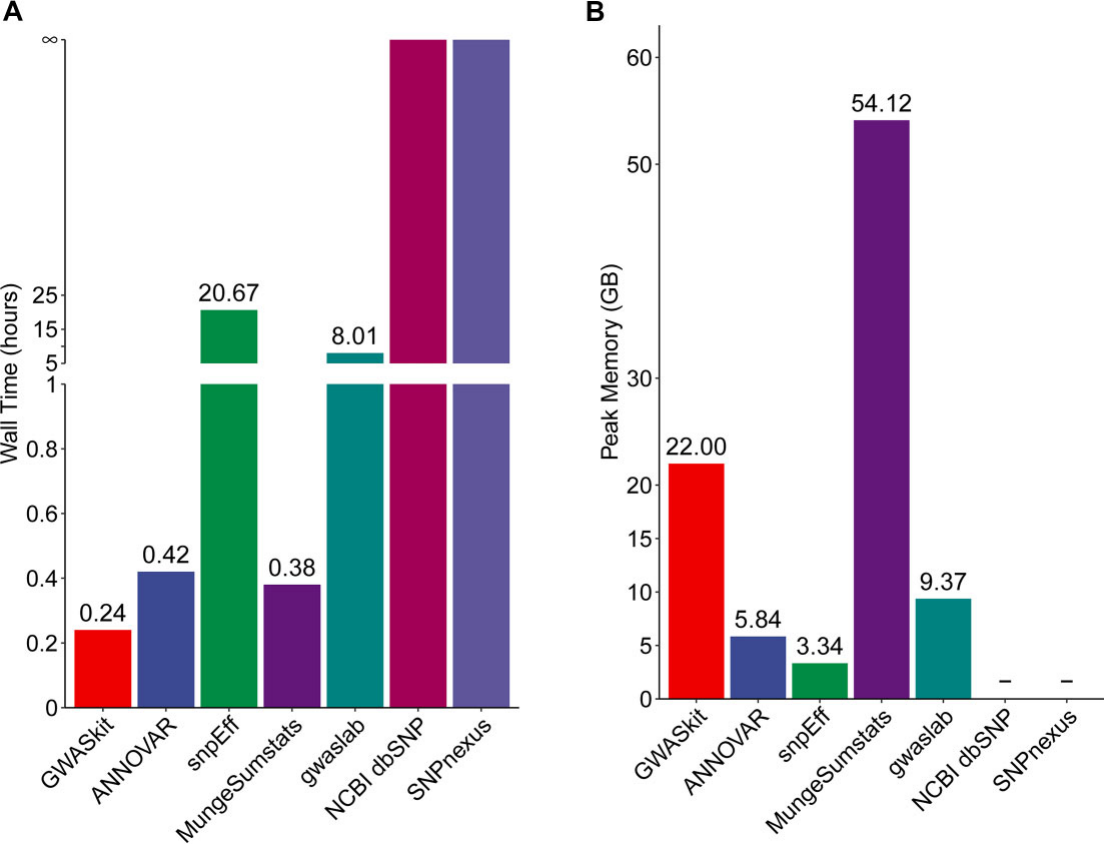
Runtime (A) and peak memory consumption (B) by different tools. Test data: large-scale GWAS summary data (GCST90236305) downloaded from the EMBL-EBI GWAS catalog database, which contained 14,519,897 variants with complete information, including chromosome, location, other allele, effect allele, and rs ID details. Test environment: Linux Ubuntu 20.04.4 LTS operating system, equipped with a 16-core Intel (CPU), 128 GB of RAM, and a 12 TB hard drive.

To evaluate the performance of rs ID conversion by GWASkit and other existing tools, we used GWAS summary data downloaded from the GWAS catalog as test data. Figure 5A illustrates the runtime for rs ID conversion using GWASkit and 6 other existing tools (ANNOVAR [39], snpEff [40], MungeSumstats [41], gwaslab [42], NCBI dbSNP database, and SNPnexus [43]). In comparison, GWASkit required the shortest time to complete the rs ID conversion, taking only 0.24 hours (14.40 minutes), while ANNOVAR, MungeSumstats, and gwaslab required 0.42, 0.38, and 8.01 hours, respectively (Fig. 5A). Meanwhile, snpEff required the longest time, 20.67 hours. It is important to note that snpEff is a genetic variant annotation and functional effect prediction toolbox, which simultaneously annotates many other pieces of information, resulting in a more time-consuming process. In terms of RAM usage, ANNOVAR and snpEff required similar amounts of memory consumption but much less than MungeSumstats (Fig. 5B). The high memory requirement of MungeSumstats is likely due to the R compiler’s tendency to use more memory. In comparison, GWASkit uses a moderate amount of memory, 22.00 GB, primarily to achieve better performance. We also tested GWASkit on a Windows computer with 8 GB of available memory, and it worked normally, although the runtime was extended by approximately 1 hour.

When comparing different tools from various perspectives (Table 2), we find that the choice of tool should be based on specific usage scenarios. For batch processing needs, local tools are more recommended due to their efficient processing capabilities; conversely, if only several SNPs need to be annotated, direct use of some online tools, such as the NCBI dbSNP database or SNPnexus, is sufficiently convenient. Considering that GWAS summary data are usually very large, often reaching hundreds of megabytes (MB) or even 1 or 2 gigabytes (GB), the advantages of local tools for processing such large data are particularly evident (Fig. 5). It is worth noting that most of these tools are developed based on the Linux operating system or require certain programming skills, posing some requirements for users. From the user’s perspective, accuracy is the primary indicator for evaluating tool performance, while the simplicity of tool installation and ease of use are also important, as they directly set a threshold that limits more researchers from using it. Regarding accuracy, our tool GWASkit significantly outperforms other tools with its extremely high accuracy rate (99.999993%, close to 100%; Table 2), followed by ANNOVAR and snpEff with accuracy rates of 97.25% and 98.87%, respectively. In contrast, MungeSumstats has the lowest accuracy rate of only 59.67%, which somewhat limits its application in high-accuracy scenarios.

**Table 2:** Feature comparisons of currently available rs ID conversion tools (local and web tools)

Tool	Type	Platforms	Standalone	User interface	Batch	Accuracy (%)
GWASkit	Local	Linux, Windows	Yes	UI for Windows	Yes	100.0
ANNOVAR	Local	Linux	Perl Scripts	No	Yes	97.25
snpEff	Local	Linux	JAR file	No	Yes	98.87
MungeSumstats	Local	Linux, Windows	R package	No	Yes	59.67
gwaslab	Local	Linux, Windows	Python module	No	Yes	98.35
NCBI dbSNP	Online	—	—	Yes	No	—
SNPnexus	Online	—	—	Yes	Yes^a^	—

^a^SNPnexus limits the maximum number of variants in a single batch query to 10,000. UI: user interface.

Overall, GWASkit not only stands out with its excellent accuracy but is also particularly suitable for researchers with limited or even no programming skills. GWASkit is a standalone binary executable file that avoids complex installation steps and supports multiple operating systems (Table 2). Especially with its Windows version, which features a simple user interface (UI), users can complete tasks that typically require programming skills and complex installation by just clicking and entering necessary information. This greatly lowers the usage threshold and enhances efficiency. Additionally, using GWASkit for rs ID conversion can be done with just a single command.

### Case II: GWAS summary data standardization

The VCF is a standard text file format widely used in bioinformatics for storing gene sequence variations. VCF files facilitate the integration of GWAS summary data with other genomic datasets by providing metrics and filters that ensure only reliable variants are considered. The standardized format allows them to be used across different tools and platforms, making VCF files a versatile choice for researchers. GWASkit provides functions for standardizing the format of GWAS summary data, ensuring that datasets from a wide range of sources are as interoperable as possible. Most importantly, for local MR analysis applications (e.g., 2-sample MR analysis), reading a large-scale GWAS summary data with R scripts typically requires a significant amount of memory and is time-consuming. For example, processing 500-MB GWAS summary data locally might use 8 to 10 GB of memory and take about 20 minutes. To address this, MRanalysis has optimized the process by using standard VCF format files for local analysis, reducing the time to just 2 to 3 minutes and requiring only 1 to 2 GB of memory.

To illustrate the versatility of GWASkit in converting GWAS statistics from various formats into the standardized VCF format, we provided a comprehensive walkthrough using the MDD and FI data from Table 1. The MDD data posed a challenge, as they only included rs ID information, lacking some crucial details such as chromosome number and basepair location. To address this issue, we employed the “rs2pos” subcommand of GWASkit, which efficiently filled the missing information. The process took approximately 12.93 minutes and had a peak memory usage of 17.79 GB. Upon completion of the data augmentation, we proceeded to utilize the “tsv2vcf” subcommand to perform a standardization of the data. This step required 3.27 minutes and had a peak memory usage of 6.18 GB. In contrast, the FI dataset already contained all the necessary information, allowing us to directly apply the “tsv2vcf” for standardization, which also took about 3 minutes and had a peak memory usage of 7.33 GB. By successfully converting the MDD and FI GWAS data into standardized VCF format, we prepared them for subsequent MR analysis.

GWASkit’s robust data processing and format conversion capabilities offer researchers an efficient and user-friendly tool to tackle GWAS data in various formats from various databases. The streamlined workflow provided by GWSAkit, from data augmentation to standardization, not only saves time and computational resources but also ensures data consistency and compatibility across different platforms. This standardization is particularly crucial for large-scale meta-analysis and collaborative research efforts, where data from multiple sources need to be integrated and analyzed together. Moreover, the reduced memory usage and processing time achieved by MRanalysis using standardized VCF files significantly enhance the accessibility and feasibility of local MR analyses. This optimization enables researchers with limited computational resources to perform complex analyses on their own machines.

### Case III: GWAS summary data quality control

**Figure 6:**
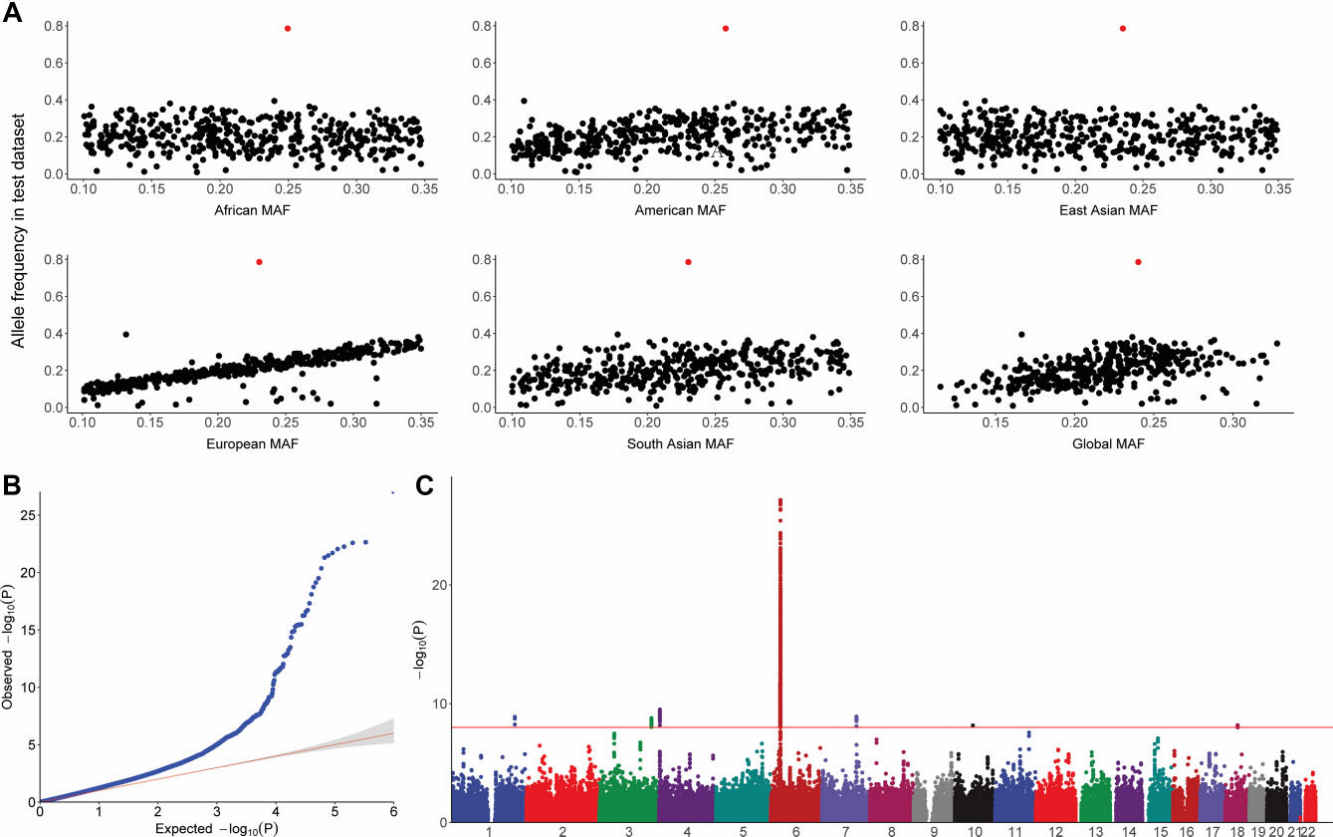
Quality control of GWAS summary data of frailty index. (A) Allele frequency in test dataset (frailty index) versus 1000 Genomes super populations. Allele frequencies are expected to be <0.50 (black points). A high allele frequency conflict is defined as an allele frequency of >0.58 (red points) in the test data or if the allele frequency differs by >10 points between the test and reference datasets. Moderate allele frequency conflicts are allele frequencies of >0.50 but $ \le$ 0.58. (B) Q–Q plot of the GWAS dataset. The observed results deviate significantly from the expected results, suggesting that the effects of these loci exceed random effects and may be significantly associated with the phenotype. (C) Manhattan plot of the GWAS dataset. The horizontal axis represents the genomic locations of all the tested SNPs in physical order. The vertical axis shows −10log *P* values for individual variant association with frailty index. Red lines indicate the threshold for genome-wide significance at $5 \times 10^{-8}$.

The comparison of allele frequencies between the FI GWAS dataset and the 1000 Genomes reference population data allows for a systematic examination of the accuracy and consistency of the genetic data, establishing a solid foundation for subsequent genetic statistical analyses. In this case, we used the CheckSumStats application to intuitively present the distribution patterns of SNP allele frequencies in the 2 datasets. As shown in Fig. 6A, black points represent SNP loci with consistent frequencies, while red points represent SNP loci with frequency conflicts. Most SNPs have frequencies less than 0.5 and are consistent between the 2 datasets, suggesting that the reported effect allele frequencies in this case dataset can accurately correspond to the effect alleles themselves. However, a considerable proportion of SNPs exhibit frequency conflicts, and the speculated reason may be that the effect allele frequency column confuses effect alleles and noneffect alleles, actually recording the minor allele frequency. The identification of allele frequency conflicts underscores the importance of careful data curation and quality control in genetic association studies. Misclassification of effect and noneffect alleles can introduce biases and lead to erroneous conclusions. Therefore, it is crucial to implement robust data cleaning and validation procedures to ensure the accuracy and reliability of the dataset before conducting downstream analyses.

The Q–Q plot (Fig. 6B) shows that the observed results deviated significantly from the expected results at lower *P* values, suggesting that the effect of these loci exceeds random effects and might be significantly associated with the phenotype. This deviation from the expected distribution indicates the presence of true associations between the genetic variants and the FI, warranting further investigation into the biological mechanism underlying these associations. The Manhattan plot (Fig. 6C) revealed that a SNP cluster on chromosome 6 showed the most significant association with the FI. This finding highlights the potential importance of genetic variants in this region in influencing an individual’s susceptibility to frailty. In conclusion, the CheckSumStats, in conjunction with the Q–Q plot and Manhattan plot, provides a comprehensive assessment of the GWAS datasets.

### Case IV: Two-sample Mendelian randomization

In this case, the findings of causality between MDD and FI of Wang et al. [33] were replicated using our web platform. Two approaches were employed: (i) using the local mode of 2-sample MR analysis with the standardized GWAS dataset (cases I, II, and III) and (ii) directly utilizing the API mode application to access online data from the IEU OpenGWAS project. Remarkably, the results obtained from both approaches were nearly identical, demonstrating the robustness and reproducibility of the findings. Some minor discrepancies observed between the 2 approaches can likely be attributed to the personalized processing and curation steps implemented by IEU OpenGWAS during their data cleaning and preprocessing pipeline.

**Figure 7:**
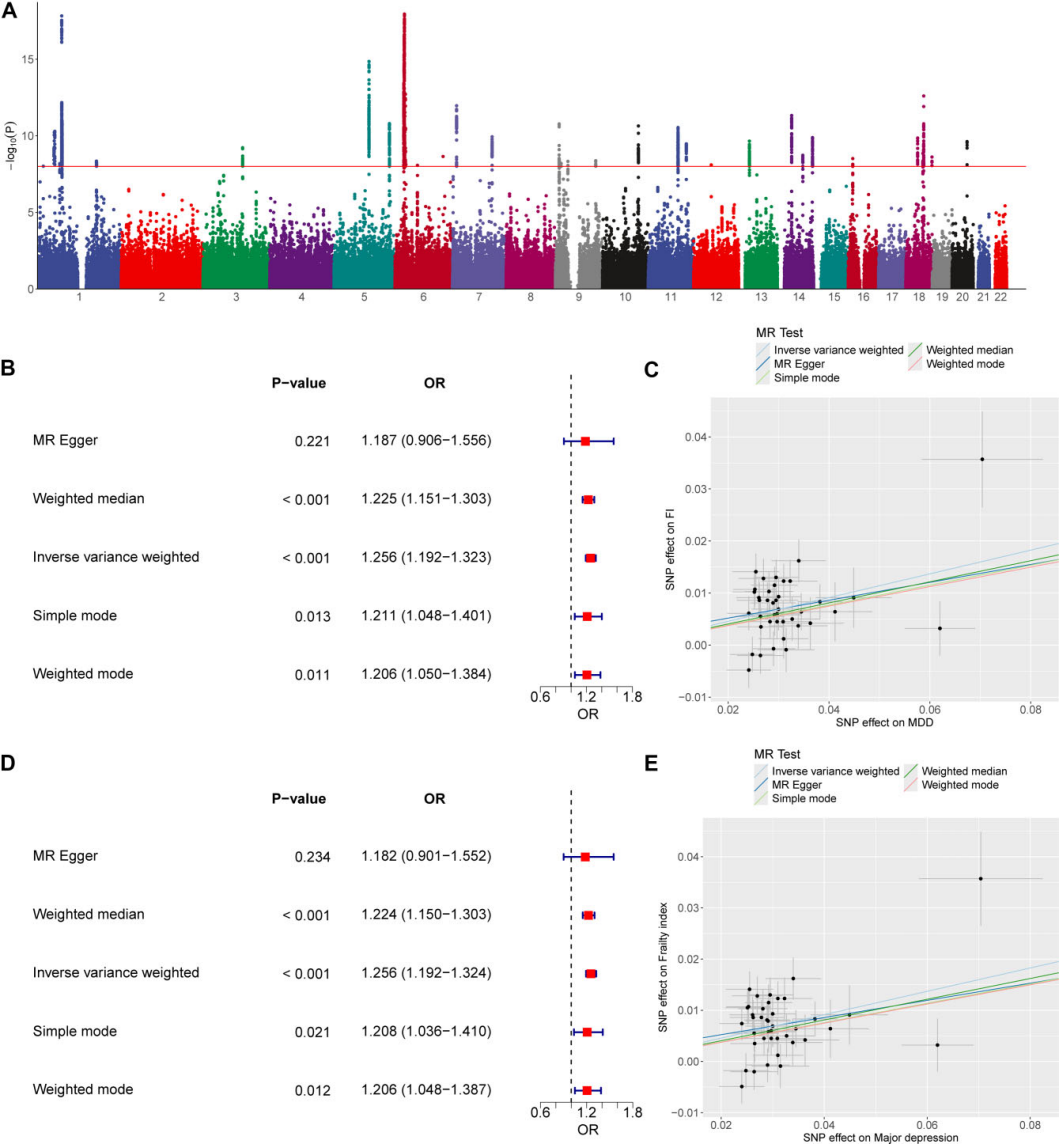
The results of 2SMR analysis. (A) Manhattan plot showing distribution of *P* values from genome-wide association study of MDD. (B) Forest plot of MR analysis of the MDD and FI using local mode. (C) Individual estimates about the effect of MDD on FI using local mode. The x-axis shows the SNP effect and SE on each of the instrumental variables of MDD. The y-axis shows the SNP effect and SE on FI. Analyses were conducted by using MR-Egger, weighted median, inverse variance weighted, simple mode, and weighted mode. The slope of each line corresponds to the estimated MR effect per method. (D) Forest plot of MR analysis of MDD (ieu-b-102) and FI (ebi-a-GCST90020053) using API mode. (E) Individual estimates about the effect of MDD on FI using API mode. 2SMR: 2-sample Mendelian randomization; API: application programming interface; FI: frailty index; MDD, major depressive disorder; OR: odds ratio; SE: standard error; SNP: single-nucleotide polymorphism.

In this replication study, a total of 3,008 genetic variants associated with MDD reached genome-wide significance ($P < 5 \times 10^{-8}$) (Fig. 7A). Of these, 50 SNPs were selected as the IVs. The *F*-statistics for the IVs ranged from 29.7519 to 78.4487, all exceeding the threshold of 10, indicating that the IVs were not biased by weak instruments. This ensures the validity of the selected genetic variants as robust IVs of MDD in the MR analysis. The IVW analysis showed that the genetic changes in the MDD were statistically associated with an increased risk of FI (local mode: OR = 1.256, 95% confidence interval [CI]: 1.192–1.323, *P* < 0.001, Fig. 7B; API mode: OR = 1.256, 95% CI: 1.192–1.324, *P* < 0.001, Fig. 7D), with some heterogeneities observed among IVs (*Q* = 73.3864 and 74.5128 in local and API mode, respectively, and *P* < 0.001 both in 2 modes). The causality between MDD and FI was also further confirmed by other MR methods, including the MR-Egger, weight median, simple mode, and weight mode (Fig. 7B, D). The scatterplot and trend line showed the consistent trend of causal relationship between MDD and FI for all 5 MR methods (Fig. 7C, E). To assess the presence of horizontal pleiotropy, which can bias the MR estimates, the MR-Egger intercept test and MR-PRESSO distortion test were performed. Both tests showed no indication of horizontal pleiotropy (all *P* values greater than 0.05), supporting the validity of the MR assumptions and the reliability of the causal estimates.

The consistency of results across different data sources and processing methods highlights the reliability and validity of the MRanalysis platform in conducting MR analyses. By offering both local and API-based data integration options. MRanalysis provides researchers with the flexibility to choose the most suitable approach based on their specific requirements and data availability. This versatility ensures that researchers can conduct MR analyses using the most appropriate and up-to-date data sources while maintaining the integrity and comparability of the results.

### Case V: Multivariable Mendelian randomization

In this case, we investigated the potential causal relationship between creatinine and CHD, considering the possibility that IVs for creatinine could act through lipid species like triglycerides to influence the risk of CHD. To account for this potential mediation, we constructed MVMR models that estimate the creatinine to CHD relationship conditioned on HDL cholesterol, LDL cholesterol, and triglycerides.

**Figure 8:**
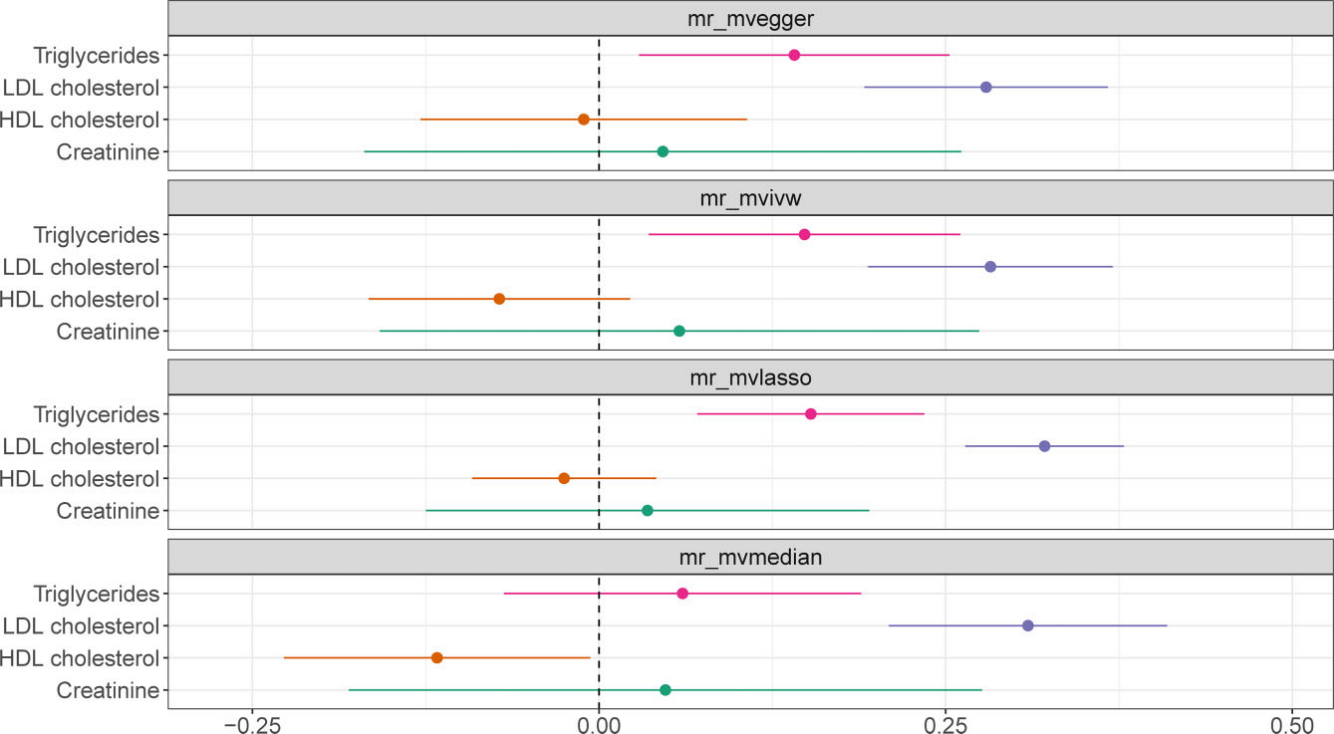
Multivariable Mendelian randomization (MVMR) models investigating the effect of creatinine and major lipid species on coronary heart disease. Each panel represents the results from a different MVMR model (each with different underlying assumptions). HDL cholesterol: high-density lipoprotein cholesterol; IVW: inverse variance weighted; LDL cholesterol: low-density lipoprotein cholesterol; MR: Mendelian randomization.

To further investigate the independent effects of creatinine and major lipid species on CHD, we screened 145 SNPs as IVs for the MVMR. The MVMR-IVW method showed that triglycerides (OR = 1.16, 95% CI: 1.04–1.30, *P* = 0.010) and LDL cholesterol (OR = 1.33, 95% CI: 1.21–1.45, *P* < 0.001) were significantly associated with the risk of CHD. In contrast, HDL cholesterol (OR = 0.93, 95% CI: 0.85–1.02, *P* = 0.135) and creatinine (OR = 1.06, 95% CI: 0.85–1.32, *P* = 0.600) were not significantly associated with the risk of CHD (Fig. 8). These results suggest that higher levels of triglycerides and LDL cholesterol may causally contribute to the development of CHD, independent of the effects of creatinine and HDL cholesterol. The consistency of the results across the remaining 4 MVMR methods further strengthens the reliability of these findings. The agreement between different MR methods, each with its own assumptions and robustness properties, provides additional confidence in the conclusions drawn from the analysis.

### Case VI: 2-step or mediation Mendelian randomization

**Figure 9:**
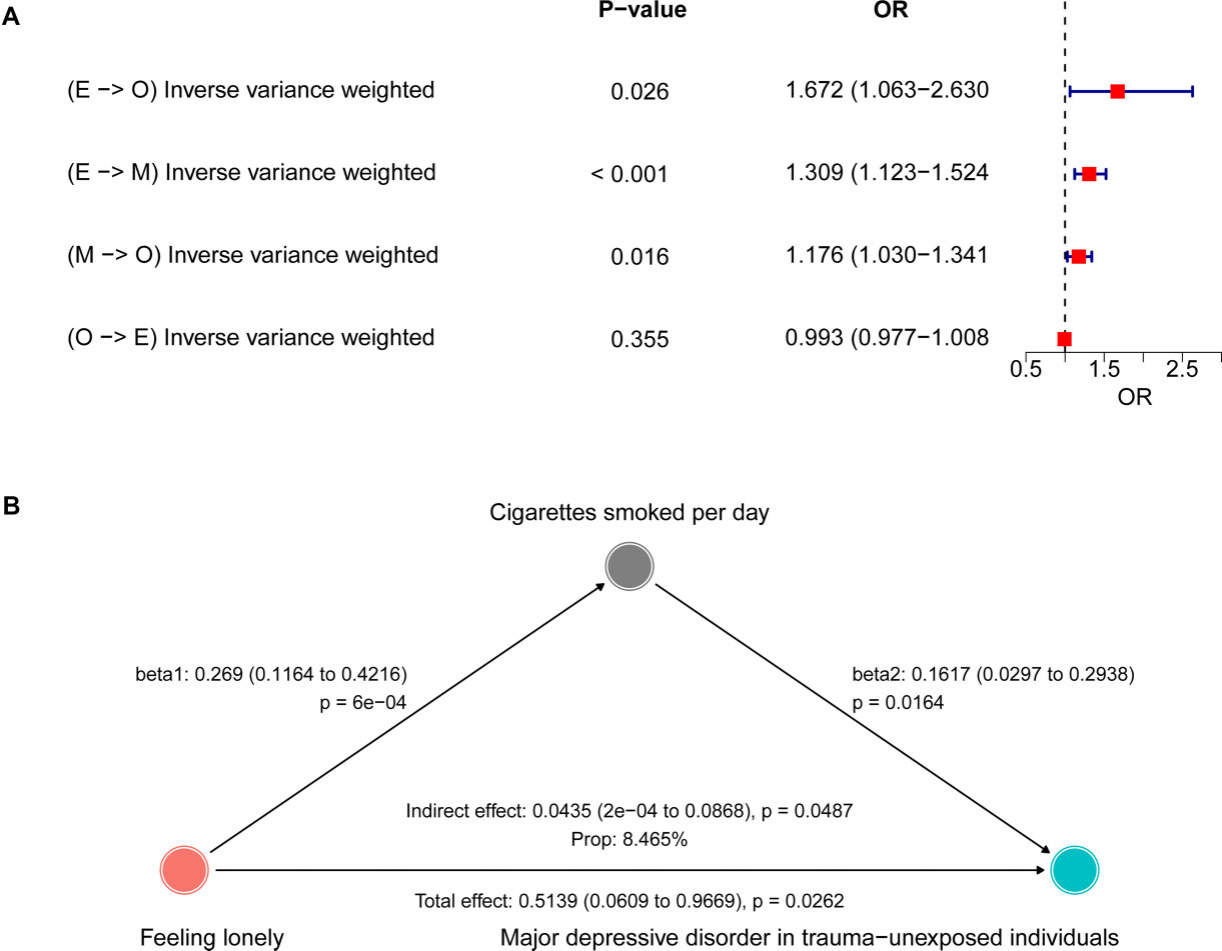
The results of mediation Mendelian randomization (MMR) analysis. (A) Forest plot of 4-step, 2-sample MR approaches. (B) Directed acyclic graph for the causal mediation analyses for the complex relationships between feeling lonely, major depressive disorder in trauma-unexposed individuals, and cigarettes smoked per day. E: exposure; O: outcome; ML mediation; MDD, major depressive disorder in trauma-unexposed individuals; MR: Mendelian randomization; OR, odds ratio.

This study employs a two-sample Mendelian randomization (MR) framework to investigate the complex relationships between feeling lonely, major depressive disorder (MDD) in trauma-unexposed individuals, and cigarettes smoked per day. The investigation consists of two main components: a bidirectional MR analysis to assess the causal directionality between feeling lonely and MDD, and a 2-step mediation MR analysis to explore the potential role of cigarettes smoked per day as a mediator in this relationship. The result of the IVW method shows that the genetic changes in feeling lonely were statistically associated with the risk of MDD (OR = 1.67, 95% CI: 1.06–2.63, *P* = 0.026, Fig. 9A), but the SNP changes in the MDD were not significantly associated with feeling lonely (OR = 0.99, 95% CI: 0.98–1.00, *P* = 0.355, Fig. 9A), indicating that the causal relationship between feeling lonely and MDD may be unidirectional. In the analysis of the exposure–mediator relationship, the IVW results indicate a positive causal relationship between feeling lonely and cigarettes smoked per day (OR = 1.31, 95% CI: 1.12–1.52, *P* < 0.001, Fig. 9A). This finding suggests that individuals who feel lonely may be more likely to engage in smoking behavior, potentially as a coping mechanism or due to shared underlying factors. Furthermore, for the mediator to outcome relationship, IVW results suggest that cigarettes smoked per day significantly increase the risk of MDD (OR = 1.18, 95% CI: 1.03–1.34, *P* = 0.016, Fig. 9A).

Based on the findings of step 1 (feeling lonely to MDD), step 3 (feeling lonely to cigarettes smoked per day), and step 4 (cigarettes smoked per day to MDD), the mediating role of cigarettes smoked per day in the relationship between feeling lonely and MDD has been demonstrated (Fig. 9B). The mediating effect of cigarettes smoked per day in increasing the risk of MDD through feeling lonely was statistically significant ($\beta$ = 0.044; 95% CI: 0.0003–0.0868, *P* = 0.049). The mediation proportion, indicating the proportion of the total effect of feeling lonely on MDD that is mediated by cigarettes smoked per day, was estimated to be 8.47.

### Case VII: SNP gene mapping and enrichment

**Figure 10:**
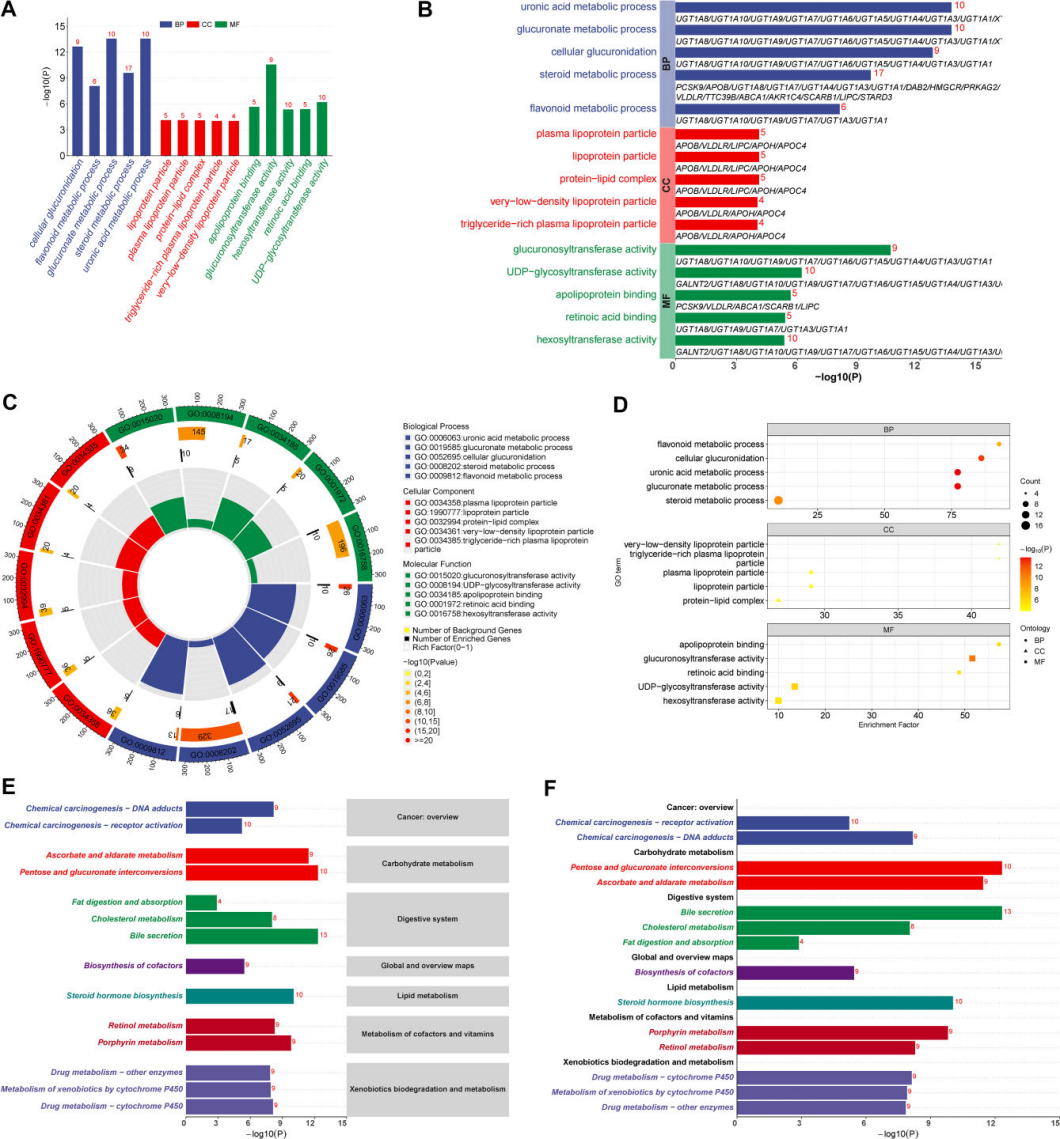
The results of GO/KEGG enrichment analyses. (A) A vertical bar chart displaying GO enrichment. (B) A horizontal bar chart displaying GO enrichment. (C) A circos plot displaying GO enrichment. (D) A dot plot displaying GO enrichment. (E) A hierarchical horizontal bar chart displaying KEGG enrichment (style I). (F) A hierarchical horizontal bar chart displaying KEGG enrichment (style II). BP: biological process; CC: cellular component; GO: Gene Ontology; MF: molecular function.

In case V, a multivariable Mendelian randomization analysis was used to study the potential causal effects of creatinine and major lipid species on CHD. To further study the functional implications of the genetic variants associated with CHD, the MAGMA plugin was employed to annotate the 146 IVs to their corresponding genes. This annotate resulted in a set of 99 genes. MAGMA uses MSigDB by default for enrichment analysis, while its extensibility allows seamless integration with external bioinformatics tools to further explore the functional impact of identified genes. In this case study, we leveraged MAGMA’s capabilities to map SNPs to their corresponding genes. This crucial step bridges the gap between genetic variants and their potential functional impacts at the gene level. Following this mapping process, we conducted enrichment analyses using applications of GO and KEGG enrichment analysis. To enhance the interpretability and visual representation of our results, we imported the enrichment analysis outputs into the visualization module of MRanalysis. This visualization module offers a suite of powerful visualization tools, including circos plots, bar plots, and dot plots (Fig. 10). These enrichment analyses provide valuable insights into the biological processes (BP), molecular functions (MF), and cellular components (CC) associated with the identified genes, as well as their involvement in various biological pathways in CHD.

MAGMA plugin is a powerful application that combines robust statistical methodology with an intuitive user interface, enabling researchers to uncover novel insights into the genetic architecture of complex traits. By integrating genetic association data with functional genomic annotations, MAGMA/GO/KEGG applications facilitate the identification of biologically meaningful gene sets and pathways, ultimately advancing our understanding of the underlying biological mechanisms. By integrating genetic association data with functional genomic annotations and enabling seamless integration with external bioinformatics tools, enrichment analysis empowers users to uncover novel insights into the underlying biological mechanisms.

### Case VIII: Minimum sample size estimation

Using the MRanalysis SampleSizeCalculator, we estimated the minimum total sample size required to achieve 80% power at $\alpha$ = 0.05 for each exposure–outcome pair and compared these thresholds with the available GWAS outcome sample sizes. Five of 9 analyses met or exceeded the predicted minimum and were therefore adequately powered at the observed effect sizes, while 4 were underpowered. Specifically, MDD–frailty index (*N*_available = 175,226 vs. *N*_required = 52,600; 3.3$\times$), feeling lonely–MDD (500,199 vs. 203,700; 2.5$\times$), MDD–feeling lonely (376,352 vs. 41,900; 9.0$\times$), LDL cholesterol–coronary heart disease (184,305 vs. 4,200; 44$\times$), and triglycerides– coronary heart disease (184,305 vs. 25,100; 7.3$\times$) were all adequately powered. In contrast, feeling lonely–cigarettes smoked per day (249,752 vs. 3,250,800; 0.08$\times$), cigarettes smoked per day–MDD (500,199 vs. 1,241,241,600; 0.0004$\times$), HDL cholesterol–coronary heart disease (184,305 vs. 538,500; 0.34$\times$), and creatinine–coronary heart disease (184,305 vs. 2,513,800; 0.07$\times$) were underpowered, with the smoking-to-MDD analysis particularly constrained by an effect estimate near the null (log OR $\approx$ 0.0014; OR $\approx$ 1.0014), implying an unrealistically large required sample size (Table 3).

**Table 3:** Feature comparisons of currently available rs ID conversion tools (local and web tools)

Exposure	Outcome	Outcome type	rsq^a^	b1^b^	OR	Ratio^c^	Actual^d^	Estimated^e^
ieu-b-102	ebi-a-GCST90020053	Continuous	0.0038	0.1976	1.2185	—	175,226	52,600
ebi-a-GCST006942	ieu-b-102	Binary	0.0036	0.2187	1.2445	0.5183	500,199	203,700
ieu-b-102	ebi-a-GCST006942	Continuous	0.0097	0.1393	1.1494	—	376,352	41,900
ebi-a-GCST006942	ieu-b-142	Continuous	0.0037	0.0255	1.0258	—	249,752	3,250,800
ieu-b-142	ieu-b-102	Binary	0.0139	0.0014	1.0014	0.5183	500,199	1,241,241,600
ieu-a-299	ieu-a-7	Binary	0.0537	0.0350	1.0357	0.4923	184,305	538,500
ieu-a-300	ieu-a-7	Binary	0.0503	0.4110	1.5084	0.4923	184,305	4,200
ieu-a-302	ieu-a-7	Binary	0.0473	0.1731	1.1890	0.4923	184,305	25,100
met-d-Creatinine	ieu-a-7	Binary	0.0358	-0.0199	0.9803	0.4923	184,305	2,513,800

^a^Coefficient of determination (*R*$^2$) of exposure on genetic variants. ^b^Causal effect. ^c^Ratio of case/controls. ^d^Actual sample size. ^e^Estimated minimum sample size.

These comparisons indicate that for adequately powered pairs, the minimum detectable effect size given the available sample size was smaller than the estimated causal effect, whereas for underpowered pairs, it exceeded the observed magnitude. For binary outcomes, calculations accounted for the case-control composition of the corresponding GWAS (case fractions 0.49–0.52, as reported). In 2-sample analyses, the outcome sample size was defined as the number of participants contributing to the variant–outcome association estimates, in accordance with the calculator’s conventions.

## Discussion

MRanalysis and GWASkit provide a comprehensive and user-friendly platform for MR analyses and processing GWAS summary data. The development of our platform addresses several key challenges in the field, such as the complexity of different MR methods, the lack of standardized workflows, and the need for extensive coding experience. By integrating data preprocessing, QC, MR analysis, and visualization, MRanalysis streamlines the entire MR workflows, making it more accessible to researchers with diverse backgrounds and varying levels of coding expertise (all applications in MRanalysis include test data, and complete operation GIFs are recorded).

While MRanalysis democratizes MR methodology for researchers across diverse backgrounds, we acknowledge the critical importance of preventing methodological misuse. Increased accessibility raises legitimate concerns about atheoretical applications—particularly conducting cross-trait analyses without biological justification or examining arbitrarily selected exposure–outcome pairs. MR’s validity as a causal inference framework fundamentally depends on satisfying core assumptions and establishing biologically plausible relationships. Users must ground analyses in well-defined, evidence-based hypotheses and adhere to established best-practice guidelines [22]. MRanalysis enforces comprehensive quality control standards, including instrument strength evaluation (*F*-statistics), heterogeneity assessment (Cochran’s *Q*), horizontal pleiotropy detection (MR-Egger intercept, MR-PRESSO), and mandatory power calculations. Violation of fundamental assumptions—relevance, independence, and exclusion restriction—particularly when selecting exposure–outcome pairs without considering biological plausibility or pleiotropic pathways, invariably yields spurious causal inferences. To facilitate methodologically sound research, MRanalysis incorporates automated quality checks, sample size calculators, transparent assumption reporting, and real-time analytical warnings. This integrated framework balances accessibility with methodological rigor, fostering responsible implementation while emphasizing that biological plausibility and methodological scrutiny remain paramount for valid causal inference in genetic epidemiology.

One of the major strengths of MRanalysis is the modular structure, which allows users to perform a wide range of analyses, from univariable and multivariable to mediation MR. Our platform also offers additional utilities, such as power and sample size calculators, SNP to gene annotation, and enrichment analysis, further enhancing the robustness and reliability of MR studies. The incorporation of best practices and guideline proposed by Burgess et al. [22] ensures the standardization of MR analyses and improves the quality and reproducibility of the results. Interpretation of MR results requires comprehensive evidence synthesis rather than reliance on isolated analyses. MRanalysis promotes a robust causal inference framework that integrates multiple analytical strategies, combining univariable MR for establishing basic causal relationships, multivariable MR for controlling confounding exposures, and mediation MR for elucidating causal pathways. Our platform facilitates robust validation through the integration of complementary analytical approaches, encompassing rigorous quality control procedures, colocalization analyses, fine-mapping studies, and pathway enrichment analyses where feasible. We emphasize that MR findings should be contextualized within broader evidence bases, including experimental validation, replication across diverse populations, and consistency with observational epidemiological studies. This framework treats MR results as one line of evidence for causality, not definitive proof, helping prevent overinterpretation that could mislead clinical decisions.

GWASkit, as a standalone tool and bridge, complements MRanalysis by facilitating the preprocessing of kinds of GWAS summary datasets. It is efficient handling of tasks, such as rs ID mapping, format conversion, and data standardization, enabling seamless compatibility with a wide range of post-GWAS tools and applications within the MRanalysis platform. These case studies demonstrated the versatility, convenience, and robustness of our platform. In particular, the case study on rs ID conversion shows that our GWASkit outperformed other existing tools, exhibiting high accuracy, fast processing speed, and moderate memory usage. In addition, the availability of GWASkit on multiple operating systems and its user-friendly interface make it accessible to researchers with limited programming skills.

Currently, there are several online platforms for MR analysis (Table 4), with MR-Base [44] being one of the earliest, appearing in 2018. MR-Base is an analytical platform for MR developed by the MRC IEU at the University of Bristol. This team has also developed the OpenGWAS database [50], which is a database of genetic associations from the GWAS summary dataset, available for online querying and download. Since the launch of the MR-Base and OpenGWAS database, other online tools have emerged, each with a slightly different focus. However, most of these tools primarily support the 2-sample MR approach and do not offer functionality for multivariable and mediation MR. Furthermore, these tools often lack the necessary steps for preprocessing and QC of GWAS datasets. In contrast to the existing platforms, MRanalysis is specially designed for MR analyses and related GWAS data processing. We aim to establish a comprehensive, standardized, and user-friendly open platform that caters to the needs of users conducting MR studies. By providing a complete workflow that includes data preprocessing, QC, and various MR approaches, our platform seeks to address the limitations of current platforms and facilitate more robust and reproducible MR analyses.

**Table 4:** Feature comparisons of currently available Mendelian randomization analysis platform

Platform	Input	Data preprocessing	Free	QC	2SMR	MVMR	MMR	Colocalization analysis	Visualization	Year	Reference
**MRanalysis**	**BI, UP**	**Yes**	**Yes**	**Yes**	**Yes**	**Yes**	**Yes**	**Yes**	**Yes**	**2025** ^a^	—
MR-base	BI, UP^b^	No	Yes	No	Yes	No	No	No	No	2018	Hemani et al. [44]
MetaboAnalyst	BI^c^	No	Yes	No	Yes	No	No	No	Yes	2024	Pang et al. [45]
HiOmics	BI, UP	No	No	No	Yes	No	No	No	Yes	2023	Li et al. [46]
SUMMER	BI^d^	No	Yes	No	Yes	No	No	No	Yes	2019	Xin et al. [47]
MRbrowse	BI^e^	No	Yes	No	Yes	No	No	No	Yes	2018	—
ExPheWas	BI^f^	No	Yes	No	Yes	No	No	No	Yes	2021	Legault et al. [48]
MRAD	BI^g^	No	Yes	No	Yes	No	No	No	No	2024	Zhao et al. [49]

2SMR: 2-sample MR analysis; BI: built-in; MMR: mediation MR; MR: Mendelian randomization; MVMR: multivariable MR; QC: quality control; UP: upload.

^a^Last update time. ^b^Only the exposure data can be uploaded by users, while the outcome data are not supported for user uploads. ^c^The exposure data primarily focus on metabolites. ^d^The outcome data primarily focus on cancers. ^e^Preperformed Mendelian randomization (MR) analyses of hundreds of proteins against hundreds of clinical outcomes using genetic data. ^f^The platform reports on genetic associations between genes and phenotypes. ^g^The MRAD application was created to identify the risk of protective factors for Alzheimer’s disease.

Another key strength of MRanalysis lies in its transparency and reproducibility. By providing full access to the underlying code, we empower researchers to not only understand the intricacies of their analyses but also modify and extend them as needed. This approach fosters a deeper engagement with the analytical process and promotes methodological rigor in MR studies. By integrating best practices and standardized workflows, we strive to ensure the reliability and consistency of MR analyses conducted using our platform. A crucial feature of MRanalysis is the real-time generation of complete code for all applications based on user-customized parameters. This allows users to gain a deeper understanding of the analytical steps involved. Simultaneously, those with extensive programming experience may prefer more flexible and customizable options. Leveraging this feature, users can generate code from the applications and then perform further personalized analyses locally. This capability bridges the gap between user-friendly interfaces and the need for advanced customization, catering to researchers with varying levels of technical expertise.

However, it is important to acknowledge some limitations and future directions for MR analysis and GWASkit. Several methodological limitations inherent to 2-sample Mendelian randomization necessitate careful consideration during result interpretation. The fundamental assumption of shared genetic architecture and linkage disequilibrium patterns between exposure and outcome GWAS cohorts may be compromised when integrating studies across diverse ancestral populations or geographic regions. Differential population stratification between samples can introduce systematic bias, thereby undermining the validity of causal estimates. Furthermore, 2-sample MR quantifies lifelong genetic effects, which may not accurately reflect the therapeutic potential of short-term interventions or late-onset exposures of clinical relevance. The methodology remains vulnerable to horizontal pleiotropy, wherein genetic instruments influence outcomes through biological pathways independent of the target exposure. Detecting such pleiotropic effects using summary-level statistics presents considerable analytical challenges. Statistical power is contingent upon instrument strength and GWAS sample dimensions, with weak instrument bias persisting despite apparently adequate *F*-statistics. Additional methodological concerns include collider bias and the inability to model time-varying associations or nonlinear dose–response relationships, all of which may confound causal inference. Thus, investigators should interpret 2-sample MR findings as contributory evidence toward causal inference rather than definitive proof of causation, particularly when considering clinical translation. These methodological constraints underscore the critical importance of our platform’s emphasis on comprehensive quality control procedures and multimethod validation for responsible causal inference in biomedical research. As the field of MR continues to evolve, the platform will require regular updates to incorporate new methods and address emerging challenges. Future developments may focus on expanding the platform’s compatibility with a broader range of data formats and integrating more advanced visualization techniques to facilitate result interpretation.

## Conclusion

In summary, MRanalysis and GWASkit offer a comprehensive, efficient, and user-centric solution for conducting MR analyses and handling GWAS summary data. By providing a unified and standardized platform that integrates diverse functionalities and promotes best practices, this platform has the potential to accelerate discoveries in genetic epidemiology, ultimately leading to improved understanding of complex diseases and more targeted interventions. As the user base of MRanalysis continues to grow, it is poised to become an essential resource for the genetic epidemiology community, empowering researchers to unravel causal relationships and advance our understanding of human health and disease.

## Availability of Source Code and Requirements

- Project name: MRanalysis/GWASkit
- Project homepage: https://github.com/xingabao/MRanalysis; https://github.com/Li-OmicsLab-MPU/GWASkit
- Operating system(s): Windows, MacOS, Linux
- Programming language: R, Python, CSS, HTML, Dockerfile
- Other requirements: N/A
- License: The MRanalysis/GWASkit codebase is licensed with a CC0 1.0 license (dataset) and the MIT license.
- Bio.tools id: biotools:mranalysis (https://bio.tools/mranalysis)
- RRID:SCR_027338
- Workflow hub: 10.48546/WORKFLOWHUB.WORKFLOW.1872.1

## Abbreviations

2SMR: 2-sample univariable Mendelian randomization; API: application programming interface; BP: biological processes; CC: cellular components; CHD: coronary heart disease; CI: confidence intervals; CPU: central processing unit; CSV: comma-separated values: DAG: directed acyclic graph; FI: frailty index; GO: Gene Ontology; GWAS: genome-wide association study; HDL cholesterol: high-density lipoprotein cholesterol; IEU: Integrative Epidemiology Unit; IV: instrumental variable; IVW: inverse variance weighted; KEGG: Kyoto Encyclopedia of Genes and Genomes; LD: linkage disequilibrium; LDL cholesterol: low-density lipoprotein cholesterol; LTS: long-term support; MDD: major depressive disorder; MF: molecular functions; MMR: mediation Mendelian randomization; MR: Mendelian randomization; MVMR: multivariable Mendelian randomization; NCBI: National Center for Biotechnology Information; OR: odds ratio; PGC: Psychiatric Genomics Consortium; QC: quality control; Q–Q: quantile–quantile; RCT: randomized controlled trial; SNP: single-nucleotide polymorphism; TSV: tab-separated values; UI: user interface; VCF: variant call format.

## Ethics Statement

All test data in this study were derived from public GWAS summary-level data. Ethics approval was not required for the present study.

## Supplementary Material

giaf131_Authors_Response_To_Reviewer_Comments_Original_Submission

giaf131_GIGA-D-25-00094_Original_Submission

giaf131_GIGA-D-25-00094_Revision_1

giaf131_Reviewer_1_Report_Original_Submission Hongsheng Gui -- 5/18/2025

giaf131_Reviewer_1_Report_Revision_1 Hongsheng Gui -- 9/19/2025

giaf131_Reviewer_2_Report_Original_Submission Xia Shen -- 5/21/2025

## Data Availability

Publicly available datasets were analyzed in this study. The GWAS summary statistics can be found in the MRC IEU OpenGWAS project, the EMBL-EBI GWAS catalog, and the Edinburgh DataShare repository. The specific datasets used in this study are available under the following accession IDs: major depression (MDD): Edinburgh DataShare (doi:10.7488/ds/2458) and OpenGWAS (ieu-b-102), Howard et al. [25]; frailty index: GWAS Catalog (GCST90020053) and OpenGWAS (ebi-a-GCST90020053), Atkins et al. [24]; HDL cholesterol: OpenGWAS (ieu-a-299), Willer et al. [26]; LDL cholesterol: OpenGWAS (ieu-a-300), Willer et al. [26]; triglycerides: OpenGWAS (ieu-a-302), Willer et al. [26]; creatinine: OpenGWAS (met-d-Creatinine); coronary heart disease: OpenGWAS (ieu-a-7), Nikpay et al. [27]; feeling lonely: OpenGWAS (ebi-a-GCST006942), Nagel et al. [28]; MDD in trauma-unexposed individuals: OpenGWAS (ebi-a-GCST009981), Coleman et al. [29]; cigarettes smoked per day: OpenGWAS (ieu-b-142), Liu et al. [30]. The database search was completed on 15 August 2024.
