# Supplementary material for: MRanalysis: a comprehensive online platform for integrated, multimethod Mendelian randomization and associated post-GWAS analyses
Source: Gigascience. 2025 Oct 22;14:giaf131. doi: 10.1093/gigascience/giaf131 (PMC12616851; doi:10.1093/gigascience/giaf131)
Supplement: giaf131_GIGA-D-25-00094_Revision_1 [file giaf131_giga-d-25-00094_revision_1.pdf]

GigaScience

MRanalysis: A Comprehensive Online Platform for Integrated, Multi-Method Mendelian Randomization and Associated Post-GWAS Analyses

--Manuscript Draft--

**Manuscript Number:**	GIGA-D-25-00094R1
**Full Title:**	MRanalysis: A Comprehensive Online Platform for Integrated, Multi-Method Mendelian Randomization and Associated Post-GWAS Analyses
**Article Type:**	Research
**Funding Information:**	Macao Polytechnic University (RP/FCA-14/2023) — Prof. Kefeng Li<br>The Science and Technology Development Funds (0033/2023/RIB2) — Prof. Kefeng Li<br>Joint Research Funding Program between FDCT and the Department of Science and Technology of Guangdong Province (0009/2024/AGJ) — Prof. Kefeng Li
**Abstract:**	Background: Mendelian randomization (MR) is a powerful epidemiological method for inferring causal relationships between exposures and outcomes using genome-wide association study (GWAS) data. However, its adoption is limited by inconsistent data formats, lack of standardized workflows, and the need for programming expertise. To address these challenges, we developed MRanalysis, a user-friendly, web-based platform for integrated MR analysis, and GWASkit, a standalone tool for GWAS data preprocessing.<br>Results: MRanalysis provides a comprehensive, no-code workflow for MR analysis, including data quality assessment, power estimation, SNP-to-gene enrichment, and visualization. It supports univariable, multivariable, and mediation MR analyses through an intuitive interface. GWASkit facilitates rapid GWAS data preprocessing, such as rs ID conversion and format standardization, with significantly higher accuracy and efficiency than existing tools. Case studies demonstrate the utility and efficiency of both tools in real-world scenarios.<br>Conclusions: MRanalysis and GWASkit lower barriers to MR analysis, making it more accessible, reliable, and efficient. By democratizing MR, these tools can accelerate discoveries in genetic epidemiology, inform public health strategies, and guide targeted interventions. MRanalysis is freely available at https://mranalysis.cn, and GWASkit can be accessed at https://github.com/Li-OmicsLab-MPU/GWASkit. Together, they represent a significant advance in understanding the complex relationships between genes, exposures, and health outcomes.
**Corresponding Author:**	Kefeng Li<br>Macao Polytechnic University<br>Macao, MACAO
**Corresponding Author Secondary Information:**	
**Corresponding Author's Institution:**	Macao Polytechnic University
**Corresponding Author's Secondary Institution:**	
**First Author:**	Abao Xing
**First Author Secondary Information:**	
**Order of Authors:**	Abao Xing<br>Tiantian Cai<br>Haofan Du<br>Zhifan Li<br>Hoi Man Ng

Powered by Editorial Manager® and ProduXion Manager® from Aries Systems Corporation

	Junrong Li
	Guanmin Jiang
	Lijun Chen
	Kefeng Li
**Order of Authors Secondary Information:**	
**Response to Reviewers:**	Responses to the editor's comments (Please also see the attachment in the submission portal as a supplemental file)<br><br>We provided the "point-to-point" responses to the editor questions below (Please also see the attachment in the submission portal as a supplemental file):<br><br>Reviewer #1<br><br>In this study, Xing et al. presented an online platform for performing GWAS data preprocessing, Mendelian Randomization (MR) analyses, and post-GWAS annotation. Multiple standard tools have been implemented to ease MR steps and facilitate more robust genetic analyses to infer complex relationships between complex traits. While I see the value of developing integrated platform for MR users, I also have several major concerns regarding to its novelty, framework, and interpretation.<br><br>1. While GWASkit is a self-developed tool, all those 3 types of MR analyses and their R packages were previously developed. The novelty level for method part is not high. I am a little concerned about the copyright for implementing those existing tools and suggest adding some acknowledgement or evidence of agreement from original developers.<br><br>Response: We sincerely thank you for your thoughtful and constructive comments on our manuscript. We greatly appreciate your recognition of the potential value of our integrated platform for Mendelian randomization (MR) users, as well as your detailed feedback regarding its novelty, framework, and interpretation. Your insights are invaluable in helping us improve the quality and clarity of our work.<br>In response to your comments, we have taken proactive steps to ensure full compliance with copyright regulations and to acknowledge the contributions of original developers. Your feedback has been instrumental in guiding further improvements to our study. Please find our detailed response below:<br><br>(1)Regarding the copyright:<br><br>We have thoroughly reviewed the open-source licenses of the key R packages and tools utilized in our project. All of them are distributed under either the Unlicense, the MIT License, or the Apache License 2.0. Each of these licenses explicitly permits free use, reproduction, modification, and incorporation into other projects, especially for academic and non-commercial purposes. To further demonstrate our respect for the original developers, we have clearly acknowledged the use of these packages on our project homepage, cited them appropriately within the manuscript, and expressed our gratitude to the original authors.<br><br>(2)Enhancement and innovation in our tool beyond MR analysis:<br><br>To improve the functionality and usability of MRanalysis, we have introduced the following new features:<br>a). To address the challenge of GWAS data format heterogeneity and to improve analysis efficiency, we have developed an application that enables rapid and flexible conversion between mainstream GWAS data formats, including VCF, TSV, and CSV. Notably, our approach introduces an innovative method for parsing and processing VCF files. Traditionally, tools such as VariantAnnotation and gwasglue require more than ten minutes and substantial memory resources to load and parse large-scale GWAS files. In contrast, our optimized implementation achieves VCF parsing in approximately two to three minutes, with significantly reduced memory usage.

The address of this application: https://mranalysis.cn/plugins/FORMAT/

This improvement is achieved through a series of algorithmic and data structure optimizations tailored for GWAS summary data, allowing for seamless data integration and compatibility with downstream Mendelian randomization analyses. By providing a fast and user-friendly tool for GWAS format standardization, we facilitate reproducible and efficient MR workflows for users with varying technical backgrounds.

b). New interactive applications for colocalization analyses within MRanalysis, including support for COLOC.ABF, COLOC.SUSIE, and COLOC.eCAVIAR, expanding the scope of MRanalysis.
The address of this application: https://mranalysis.cn/plugins/COLOC/

This enhancement broadens the utility of MRanalysis, allowing users to seamlessly conduct comprehensive colocalization analyses alongside MR studies, and facilitating more reliable exploration of shared genetic signals between traits.

c). Upgraded SNP gene mapping and enrichment with comprehensive annotation and translational integration.
The address of this application: https://mranalysis.cn/plugins/MAGMA/

We have significantly enhanced the SNP gene mapping and enrichment application in MRanalysis. During SNP-to-gene annotation, the upgraded application now provides detailed gene information, including gene symbols, full gene names, and functional annotations are automatically displayed for each mapped gene, facilitating immediate biological interpretation.
To further support translational research, we have addressed the previous lack of integration with drug target and clinical trial resources. This application now incorporates direct links and annotation retrieval from widely used resources such as Open Targets, DrugBank, and ClinicalTrials.gov. For each gene, users can instantly access associated drug target data and ongoing or completed clinical trials via embedded hyperlinks to the relevant of official database entries. The seamless integration enables users to trace MR findings to known or emerging drug targets, evaluate pharmacological or clinical evidence, and more efficiently prioritize genes for follow-up functional or therapeutic investigation.

These enhancements collectively empower users to bridge genetic findings with actionable biological and clinical insights, greatly expanding the scope and translational utility of MRanalysis.

d). Open-source release and deployment optimization

GitHub: https://github.com/xingabao/MRanalysis
Docker Hub: https://hub.docker.com/r/xingabao/mranalysis

To promote transparency, reproducibility, and community engagement, we have fully open-sourced the MRanalysis codebase on GitHub. This allows users to inspect the complete workflow, report issues, and contribute to further development. The platform currently consists of over 60,000 lines of code, not only integrating numerous established packages and tools, but also implementing a wide range of self-developed features – including rs ID mapping, universal GWAS format conversion, fast VCF file parsing, and versatile data visualization applications. This comprehensive open-source release enables users to inspect the entire workflow, report issues, and contribute to further development.

Given the scale and complexity of the codebase, conventional installation can be time-consuming (often requiring 3 to 4 hours) and susceptible to software version inconsistencies. To address this, we provide an official Docker image for MRanalysis. With Docker, users can deploy the complete environment locally within 2 to 3 minutes, ensuring reproducibility and greatly lowering the barrier for ordinary users.
In addition, the Docker-based local deployment enables efficient processing of large-scale GWAS summary files and accelerates computationally intensive modules such as GWAS data quality control, MR analysis and GWAS format conversion, effectively overcoming previous limitations on data size and analysis speed.

Powered by Editorial Manager® and ProduXion Manager® from Aries Systems Corporation

In summary, we have ensured full compliance with copyright regulations, introduced multiple practical and innovative features, and provided open-source access with one-click deployment to maximize usability. Furthermore, we have duly acknowledged the developers of the original tools or packages in our project documentation and manuscript. We hope these efforts will enhance the scientific value and community impact of MRanalysis.

2. About mediation MR: the justification of defining exposure, mediator, and outcome is not clear. Without individual-level data, how valid is the estimated mediation proportion using sum data? In other words, does lonely-cigarettes-MDD result in case VI align well with traditional one-sample mediation result?

Response: Thank you for your thoughtful comments regarding the mediation MR analysis.

In case VI, we defined "feeling lonely" as the exposure, "cigarettes smoked per day" as the mediator, and "MDD" as the outcome. This pathway is justified based on established epidemiological and biological evidence: loneliness is a recognized psychosocial risk factor for mental health, and smoking is often used to cope with loneliness and stress, which in turn can influence depression risk.

Regarding the use of mediation MR with summary-level data, we fully acknowledge its limitations. Unlike traditional mediation analysis that requires individual-level data, mediation MR based on summary statistics typically adopts the product-of-coefficients method ($\beta 1\times \beta 2$) to estimate mediation effects. The method was originally proposed and developed by Burgess et al. (2015) [1] and has since been applied in several peer-reviewed studies [2,3]. However, we emphasize that this approach relies on the core MR assumptions and is subject to additional constraints inherent to the use of summary data. Direct comparison with traditional one-sample mediation analysis is not possible here due to the lack of large individual-level datasets that include all relevant variables.

Our implementation in MRanalysis is intended primarily as a methodological demonstration. We have clearly referenced the original methodology and cited relevant literature in our documentation. Users are encouraged to consider these methodological limitations when interpreting results and to consult the cited studies for further details and appropriate use cases. Ultimately, the choice to apply summary-based mediation MR should be made with caution and in the context of specific research questions.

1. Burgess, Stephen, et al. "Network Mendelian randomization: using genetic variants as instrumental variables to investigate mediation in causal pathways." International journal of epidemiology 44.2 (2015): 484-495.
2. Carter, Alice R., et al. "Mendelian randomisation for mediation analysis: current methods and challenges for implementation." European journal of epidemiology 36.5 (2021): 465-478.
3. Sanderson, Eleanor, et al. "Mendelian randomization." Nature reviews Methods primers 2.1 (2022): 6.

3. About post-MR: besides enrichment and visualization, other post-GWAS analysis (e.g., colocalization) that is supplementary to MR should be incorporated, as this will be essential for drug-target selection.

Response: Thank you very much for your valuable suggestion regarding the incorporation of additional post-GWAS analyses, such as colocalization, to supplement Mendelian randomization (MR) and enhance drug-target selection.

In response to your feedback, we have now integrated four widely used colocalization methods into our platform: 1) COLOC.ABF: Bayesian framework estimating the probability that two traits share a causal variant. 2) COLOC.SUSIE: Utilizes the Sum of Single Effects (SuSiE) model for complex multi-SNP colocalization analysis. 3)

	COLOC.PWCoCo: Hierarchical clustering-based approach to identify shared causal variants across traits. 4) COLOC.eCAVIAR: CAusal Variants Identification in Associated Regions for rigorous colocalization assessment.<br>These tools allow users to assess whether the genetic association signals for exposure and outcome are likely due to the same underlying causal variant, thereby strengthening the causal inference from MR and facilitating more robust drug-target prioritization.<br><br>We have updated the manuscript and user documentation to reflect this important addition. We appreciate your insightful suggestion, which has helped to significantly improve the scientific utility and translational relevance of our platform.<br><br>The address of this application: https://mranalysis.cn/plugins/COLOC/<br><br><br>4. Power and sample size calculation: I would suggest adding another case example for showing sample size needed for 3 types of MR analysis.<br><br>Response: Thank you for your constructive suggestion to include a case example demonstrating the minimum sample size requirements for the three types of Mendelian randomization (MR) analyses. In response, we have added Case VIII to both the manuscript (Table 3) and the MRanalysis platform.<br><br>We can utilize the integrated PowerCalculator and SampleSizeCalculator applications within the platform to estimate the statistical power and minimum required sample size for each MR analysis type. For every exposure–outcome pair in Case VIII, we reported the minimum sample size necessary to achieve 80% power at a two-sided significance level of 0.05, using the SampleSizeCalculator application and the methodology described by Burgess (2014).<br><br>The key parameters considered included the proportion of variance in the exposure explained by genetic instruments (R2), the anticipated causal effect size (odds ratio per standard deviation for binary outcomes or standard deviation change per standard deviation for continuous outcomes), the significance level (α), and, for binary outcomes, the case-control ratio. For two-sample MR analyses, the sample size was defined as the number of participants in the variant–outcome association dataset.<br><br>We believe these additions substantially enhance the comprehensiveness and practical utility of our resource. Thank you again for your valuable feedback.<br><br><br>5. About Discussion:<br>1) though it is important to ease MR analysis for all researchers, there is also a caution to avoid abusing MR for randomly selected cross-trait analysis. Need to discuss this when designing MR analysis.<br>Response: Thank you for your constructive comment regarding the potential risk of misusing Mendelian randomization (MR) for arbitrarily selected cross-trait analyses. We fully agree that, while improving accessibility to MR is important, careful consideration must be given to study design in order to avoid spurious or biologically implausible results.<br>In response, we have revised the Discussion section to explicitly address this point, emphasizing the need for clear biological rationale, robust hypothesis generation, and best-practice guidelines when selecting exposure-outcome pairs for MR analysis. We believe this addition will help users better understand the importance of proper study design and interpretation when using MRanalysis.<br>Furthermore, we have included prominent reminders on our website regarding the methodological rigor, evidence integration, and known limitations of MR, to further guide users in conducting reliable and valid analyses.<br><br>For your convenience, we also copied the revised DISCUSSION below:<br><br>2) the interpretation of MR results should come from combined evidence, for instance, combining univariable, multivariable, and mediation MR together, or combing MR with other types of evidence (fine mapping or raw-data analysis) together. Need to discuss

how to avoid overinterpretation of MR results alone.

Response: Thank you for your valuable suggestion regarding the interpretation of MR results. We agree that MR findings should not be interpreted in isolation.
Per your suggestion, in the revised Discussion, we have emphasized the importance of integrating evidence from multiple MR approaches (such as univariable, multivariable, and mediation MR) as well as from complementary sources (including fine mapping, colocalization, and raw data analyses) to strengthen causal inference and avoid overinterpretation based solely on MR results. This addition aims to encourage more robust and cautious interpretation of MR analyses conducted using our platform.
Furthermore, we have included prominent reminders on our website regarding the methodological rigor, evidence integration, and known limitations of MR, to further guide users in conducting reliable and valid analyses (also see above picture).
For your convenience, we also copied the revised DISCUSSION below:

3) Limitation of 2-sample MR should be also discussed.

Response: Thank you for your valuable comment regarding the limitations of two-sample Mendelian randomization (2SMR). We agree that it is important to acknowledge the key assumptions and potential limitations of this approach.
Per your advice, In the revised Discussion, we have added a dedicated paragraph outlining the main limitations of 2SMR, including issues related to sample overlap, heterogeneity between datasets, instrument strength, and potential bias due to horizontal pleiotropy. We believe this addition will provide a more balanced and comprehensive perspective for users of our platform.
Furthermore, we have included prominent reminders on our website regarding the methodological rigor, evidence integration, and known limitations of MR, to further guide users in conducting reliable and valid analyses (also see above picture).
For your convenience, we also copied the revised DISCUSSION below:

Reviewer #2

Review of the Manuscript: "MRanalysis: A Comprehensive Online Platform for Integrated, Multi-Method Mendelian Randomization and Associated Post-GWAS Analyses"
This manuscript introduces MRanalysis, a web-based platform designed to facilitate Mendelian Randomization (MR) analyses and associated post-GWAS workflows, supported by a companion tool, GWASkit, for formatting and validating GWAS summary statistics. The authors aim to address persistent challenges in the MR research landscape, including inconsistent summary data formats, lack of standard analytical workflows, and the technical barrier posed by programming requirements.
The platform is clearly user-oriented, providing a graphical interface that simplifies the process of performing MR analyses without coding. Features such as flexible column mapping, a variety of MR methods, and downstream utilities like gene enrichment and visualization contribute to a positive user experience.
However, while the tool is well-positioned as a front-end solution for MR analyses, several critical limitations must be addressed before the manuscript can be considered for publication.

Response: We appreciate the reviewer's positive comments and recognition for our MRanalysis tool. We have carefully revised our manuscript and the webtool based on your valuable suggestions.

1. Lack of Colocalization Analysis
The absence of integrated genetic colocalization analysis represents a fundamental weakness of the platform. In MR, particularly when used for biomarker discovery and therapeutic target identification, colocalization is essential to validate whether the genetic signal driving the exposure-outcome association is shared between traits or arises from distinct, but nearby, loci in linkage disequilibrium. Without colocalization support (e.g., COLOC, eCAVIAR, or fastENLOC), MR findings are prone to false positives and cannot be reliably interpreted as evidence of causality. This is especially problematic in highly pleiotropic genomic regions. The current version of the platform

thus risks encouraging misleading conclusions by presenting potentially spurious associations without necessary validation. Incorporating colocalization is not optional—it is a core requirement for robust causal inference.

Response: Thank you very much for your critical comment regarding the lack of integrated colocalization analysis within our platform. We fully agree that colocalization is essential for robust causal inference in Mendelian randomization (MR), particularly to distinguish whether the genetic association signals for exposure and outcome are driven by the same causal variant or by distinct, but nearby, loci in linkage disequilibrium. This is especially important for biomarker discovery and therapeutic target prioritization, as failure to account for colocalization can result in spurious associations and misleading conclusions.
In response to your feedback, we have now integrated four widely used colocalization methods into our platform: 1) COLOC.ABF: Bayesian framework estimating the probability that two traits share a causal variant. 2) COLOC.SUSIE: Utilizes the Sum of Single Effects (SuSiE) model for complex multi-SNP colocalization analysis. 3) COLOC.PWCoCo: Hierarchical clustering-based approach to identify shared causal variants across traits. 4) COLOC.eCAVIAR: CAusal Variants Identification in Associated Regions for rigorous colocalization assessment. These tools allow users to assess whether the genetic association signals for exposure and outcome are likely due to the same underlying causal variant, thereby strengthening the causal inference from MR and facilitating more robust drug-target prioritization.

We have updated the manuscript, the webtool and the corresponding user documentation to reflect this important addition (https://mranalysis.cn/plugins/COLOC/). We appreciate your insightful suggestion, which has helped to significantly improve the scientific utility and translational relevance of our platform.

2. Lack of Integration with Drug Target and Clinical Trial Databases
One of the most compelling applications of MR is in identifying and prioritizing potential drug targets. However, MRanalysis currently lacks integration with widely used drug target and trial resources such as Open Targets, DrugBank, or ClinicalTrials.gov. This omission limits the utility of the tool in translational research and biomarker evaluation. Users should be able to trace MR findings to known or emerging drug targets and assess whether implicated genes are supported by clinical or pharmacological evidence.

Response: Thank you for highlighting the importance of integrating drug target and clinical trial databases for translational research. In the latest version of MRanalysis, we have addressed this limitation by incorporating direct links to widely used resources such as Open Targets, DrugBank, and ClinicalTrials.gov. For each prioritized gene, the platform now displays drug target annotations and provides convenient hyperlinks to relevant entries in these databases. This allows users to rapidly trace MR findings to known or emerging drug targets and access supporting clinical or pharmacological evidence.
We believe these enhancements substantially strengthen MRanalysis as a tool for biomarker evaluation and translational research.

The address of this application: https://mranalysis.cn/plugins/MAGMA/

3. Limited Support for Large Datasets
At present, the platform can only process relatively small GWAS summary files, which is a practical limitation given the size of modern GWAS meta-analyses. Support for large-scale data, potentially via cloud integration or offline batch processing, is necessary for broad adoption.

Response: GitHub: https://github.com/xingabao/MRanalysis
Docker Hub: https://hub.docker.com/r/xingabao/mranalysis

Thank you very much for highlighting the current limitation regarding the processing of large GWAS summary data. We agree that the ability to efficiently handle large-scale

Powered by Editorial Manager® and ProduXion Manager® from Aries Systems Corporation

	datasets is essential for the broad adoption of our platform, especially given the increasing size of modern GWAS meta-analyses.<br><br>To address this, we have made several significant enhancements:<br>a). Docker-based deployment. We now provide a Docker image that allows users to easily deploy MRanalysis on their own computational infrastructure. This enables seamless and efficient processing of large datasets without the restrictions of online applications, allowing users to leverage their own hardware resources. With Docker, the entire environment can be set up locally within 2 to 3 minutes, greatly reducing installation time and avoiding software version inconsistencies that previously required 3 to 4 hours of manual setup. Comprehensive installation and usage instructions are included in the user documentation.<br>b). Open-source and extensible. The complete MRanalysis codebase is fully open-sourced on GitHub, comprising over 60,000 lines of code. Beyond integrating numerous established packages and tools, we have also developed a wide range of proprietary features – including rs ID mapping, universal GWAS format conversion, fast VCF file parsing, and versatile data visualization modules. This comprehensive release enables transparency, reproducibility, and community-driven improvement.<br>c). Enhanced GWAS data handling. A new data format conversion application has been introduced, allowing users to extract and covert common GWAS data formats (such as VCF, TSV, and CSV) into forms suitable for downstream analysis. This greatly improves the flexibility and usability of the platform for diverse large-scale GWAS summary datasets.<br>In future updates, we will continue to explore additional solutions, such as cloud-based processing and enhanced batch analysis, to further improve scalability. We sincerely appreciate your helpful suggestions, which have contributed to strengthening both the utility and accessibility of MRanalysis.<br><br><br>4. Performance Issues<br>Some computational modules, especially for multivariable MR, run slowly and may become impractical with larger datasets. Optimization of computational backends or asynchronous job handling would improve usability.<br><br>Response: Thank you for raising the important issue of computational performance, particularly regarding the speed of multivariable MR analyses on large datasets. We recognize that slow processing can hinder usability and limit the practical application of the platform as data sizes continue to grow.<br>To address this, we have implemented a series of optimizations in our computational workflow. Previously, analyses using VariantAnnotation and gwasglue to read local files could take 20 to 30 minutes to complete; with our recent improvements, processing speed has increased approximately tenfold, allowing most analyses to be completed within 2 to 3 minutes. In addition, the platform is now fully open source and supports local deployment via Docker, enabling users to utilize their own hardware for faster analysis of large-scale data (also see our response to comment #3).<br>Thank you again for your valuable feedback, which has been instrumental in guiding the ongoing development and optimization of our platform.<br><br><br>5. Gene Annotation Needs Improvement<br>In the current implementation, gene annotation results display only Gene IDs, which are not easily interpretable. Inclusion of gene symbols and functional annotations would enhance the accessibility and utility of the results.<br><br>Response: Thank you for your valuable suggestions regarding the interpretability of gene annotation results and the integration of translational resources. We fully agree that displaying only Gene IDs limits accessibility and practical utility for users. In response, we have significance enhanced the SNP-to-gene mapping and enrichment module in MRanalysis.<br>The annotation results now include not only gene symbols but also full gene names and functional annotations for each mapped gene, greatly facilitating immediate biological interpretation. To further support translational research, we have addressed the previous lack of integration with drug target and clinical trial databases. The updated application now provides direct links and annotation retrieval from widely used

	resources such as Open Targets, DrugBank, and ClinicalTrials.gov (also see our response to comment #2). For each gene, users can conveniently access relevant drug target information and ongoing or completed clinical trials via embedded hyperlinks to the official database entries.<br>These improvements make the results more user-friendly and valuable for downstream biological interpretation, enabling users to rapidly trace MR findings to known or emerging drug targets, evaluate pharmacological or clinical evidence, and prioritize genes for further functional or therapeutic investigation. We appreciate your insightful feedback, which has significantly contributed to enhancing the usability and scientific value of our platform.<br><br>The address of this application: https://mranalysis.cn/plugins/MAGMA/<br><br><br>6. The website design is acceptable, but the domain https://mranalysis.cn/ is considered insecure in several foreign institutions' networks. The authors should try to fix the issue.<br><br>Response:nThank you for bringing up the issue regarding website security and accessibility from certain institutional networks.<br>Per your suggestion, we have performed the following revisions:<br>(1)We have upgraded to a more secure and widely recognized SSL certificate. After multiple rounds of testing, we have not encountered any further warnings or issues indicating that the website is insecure.<br>(2)Additionally, to fully address any potential security concerns, we have open-sourced our application and now provide a Docker image for local deployment. This allows users to install and run MRanalysis entirely on their own infrastructure, eliminating any risks associated with web access. The open-source nature of the code also ensures full transparency, so users can verify that there are no hidden or insecure components.<br>We hope these measures will resolve the security concerns and further enhance user trust in our platform. Thank you again for your valuable feedback.
Additional Information:	
**Question**	**Response**
Are you submitting this manuscript to a special series or article collection?	No
**Experimental design and statistics**<br><br><br>Full details of the experimental design and statistical methods used should be given in the Methods section, as detailed in our Minimum Standards Reporting Checklist. Information essential to interpreting the data presented should be made available in the figure legends.<br><br><br>Have you included all the information requested in your manuscript?	No
If not, please give reasons for any omissions below.<br><br><br>as follow-up to "**Experimental design and statistics**	Our study is a workflow/application tool and does not involve experimental groups, sample collection, or health interventions. All statistical methods, algorithms, and workflows utilized in the tool are clearly described in the Methods section. Information essential to interpreting the results is included in the figure and table legends. Therefore, items such as randomization, blinding, and replication are not applicable to this study.

Powered by Editorial Manager® and ProduXion Manager® from Aries Systems Corporation

Full details of the experimental design and statistical methods used should be given in the Methods section, as detailed in our Minimum Standards Reporting Checklist. Information essential to interpreting the data presented should be made available in the figure legends.<br><br>Have you included all the information requested in your manuscript?<br><br>"	
**Resources**<br><br>A description of all resources used, including antibodies, cell lines, animals and software tools, with enough information to allow them to be uniquely identified, should be included in the Methods section. Authors are strongly encouraged to cite Research Resource Identifiers (RRIDs) for antibodies, model organisms and tools, where possible.<br><br>Have you included the information requested as detailed in our Minimum Standards Reporting Checklist?	No
If not, please give reasons for any omissions below.<br><br>as follow-up to "**Resources**<br><br>A description of all resources used, including antibodies, cell lines, animals and software tools, with enough information to allow them to be uniquely identified, should be included in the Methods section. Authors are strongly encouraged to cite Research Resource Identifiers (RRIDs) for antibodies, model organisms and tools, where possible.	Our study is a workflow/application tool that does not involve the use of antibodies, cell lines, animals, or model organisms. Instead, we provide a method for users to calculate their own sample size and perform analyses based on their data. All software tools, algorithms, and resources used in the workflow are clearly described in the Methods section to ensure reproducibility and transparency. Therefore, items such as RRIDs for antibodies or model organisms are not applicable to this study.

Have you included the information requested as detailed in our Minimum Standards Reporting Checklist?<br><br>"	
**Availability of data and materials**<br><br>All datasets and code on which the conclusions of the paper rely must be either included in your submission or deposited in publicly available repositories (where available and ethically appropriate), referencing such data using a unique identifier in the references and in the "Availability of Data and Materials" section of your manuscript.<br><br>Have you have met the above requirement as detailed in our Minimum Standards Reporting Checklist?	Yes
GigaScience has policies and guidelines in place for the use of generative AI-writing tools such as ChatGPT. If you have used such writing tools to assist with writing the manuscript this must be declared and cited in the text. Authors should not list AI-writing tools and other AI-assisted technologies as an author or co-author and should acknowledge that they are fully responsible for text generated or refined by AI-writing tools.<p><br><br>A summary of use (particularly in the introduction or among methods) needs to be included at the end of the paper, and the outputs should also be included as a supplementary file hosted in GigaDB or other open repositories. Please read our guidelines for more information. <p><br><br>By submitting to GigaScience, you are aware of the journal's AI-writing tools policy, and if you have declared use of such tools below, you have acknowledged this where appropriate in your manuscript and have made a summary of use and outputs available. <p><br><b>AI-assisted writing tools have been used in the preparation of this manuscript?	No

LaTeX - Main Document (TeX file)

Click here to access/download;LaTeX - Main Document (TeX file);main.tex

Placeholder for OUP logo oup.pdf

GigaScience, 2023, 1–17

doi: xx.xxxx/xxxx
Manuscript in Preparation
Paper

PAPER

MRanalysis: A Comprehensive Online Platform for Integrated, Multi-Method Mendelian Randomization and Associated Post-GWAS Analyses

Abao Xing[1,†], Tiantian Cai[2,†], Haofan Du[3,†], Zhifan Li[2], Hoiman Ng[4], Junrong Li[1], Guanmin Jiang[5], Lijun Chen[6,*] and Kefeng Li[1,*]

[1]Centre for Artificial Intelligence Driven Drug Discovery, Faculty of Applied Sciences, Macao Polytechnic University, Rua de Luís Gonzaga Gomes, Macao, 999078, Macao SR and [2]Big Data and Internet of Things Program, Faculty of Applied Sciences, Macao Polytechnic University, Rua de Luís Gonzaga Gomes, Macao, 999078, Macao SR and [3]School of Physics and Technology, Nanjing Normal University, Nanjing, Jiangsu, 210023, China and [4]Clinical laboratory, Kiang Wu Hospital, 999078, Macao SR and [5]Department of Clinical Laboratory, The Fifth Affiliated Hospital, Sun Yat-sen University, Zhuhai, Guangdong, 519000, China and [6]Department of Hematology and Rheumatology, Zhuhai People's Hospital (Zhuhai Hospital affiliated with Jinan University), Kangning Rord 97, Zhuhai, 519000, China

[*]kefengl@mpu.edu.mo; cljshizairenwei@163.com
[†]Contributed equally.

Abstract

Background: Mendelian randomization (MR) is a powerful epidemiological method for inferring causal relationships between exposures and outcomes using genome-wide association study (GWAS) data. However, its adoption is limited by inconsistent data formats, lack of standardized workflows, and the need for programming expertise. To address these challenges, we developed MRanalysis, a user-friendly, web-based platform for integrated MR analysis, and GWASkit, a standalone tool for GWAS data preprocessing.
Results: MRanalysis provides a comprehensive, no-code workflow for MR analysis, including data quality assessment, power estimation, SNP-to-gene enrichment, and visualization. It supports univariable, multivariable, and mediation MR analyses through an intuitive interface. GWASkit facilitates rapid GWAS data preprocessing, such as rs ID conversion and format standardization, with significantly higher accuracy and efficiency than existing tools. Case studies demonstrate the utility and efficiency of both tools in real-world scenarios.
Conclusions: MRanalysis and GWASkit lower barriers to MR analysis, making it more accessible, reliable, and efficient. By democratizing MR, these tools can accelerate discoveries in genetic epidemiology, inform public health strategies, and guide targeted interventions. MRanalysis is freely available at https://mranalysis.cn, and GWASkit can be accessed at https://github.com/Li-OmicsLab-MPU/GWASkit. Together, they represent a significant advance in understanding the complex relationships between genes, exposures, and health outcomes.

Key words: Mendelian randomization; GWAS; Online platform; MRanalysis; GWASkit; rs ID conversion; SNP-to-gene enrichement; Visualization

Compiled on: October 19, 2025.
Draft manuscript prepared by the author.

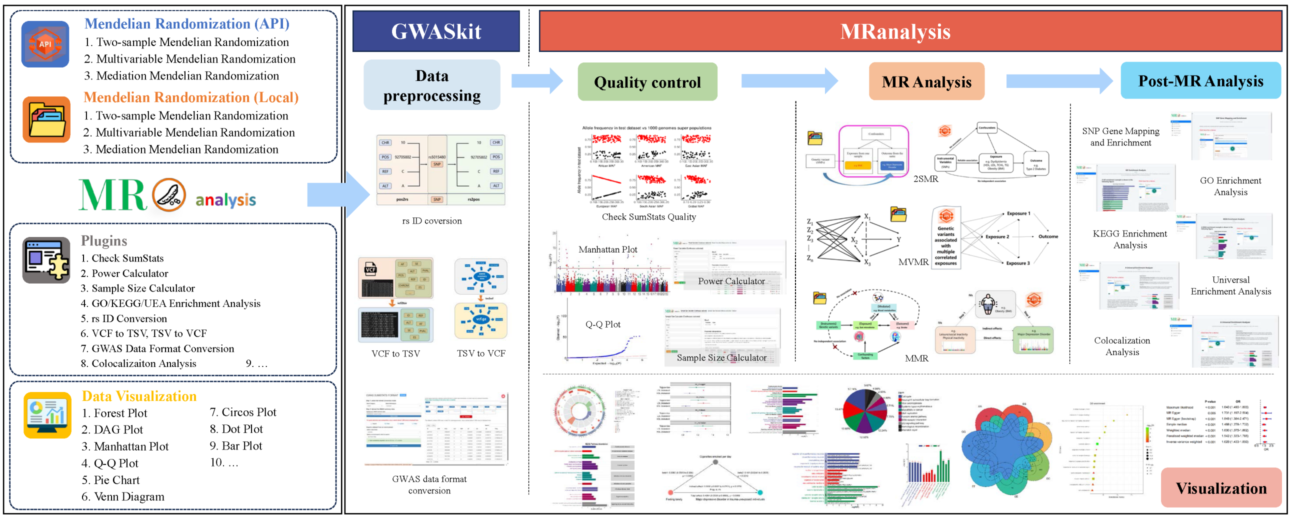

Key Points

- GWASkit Tool: A standalone, installation-free tool for rapid GWAS dataset preprocessing and format standardization, outperforming current existing tools (Such as ANNOVAR, and gwaslab) in both SNP rs ID conversion time and conversion accuracy.
- Versatility: Support various MR methodologies, including univariable, multivariable, and mediation MR analyses, catering to diverse research needs.
- Efficiency and Accuracy: Case studies demonstrate the utility, efficiency, and ease of use of both MRanalysis and GWASkit in real-world scenarios, highlighting their potential to accelerate MR research.
- Real-Time Code Generation: Generates and assembles code based on user-defined parameters, enhancing transparency and reproducibility.
- Visual Guidance: Detailed GIF tutorials for all applications, improving user experience.

Introduction

Mendelian randomization (MR) is a powerful research approach that uses genetic variants (usually SNP, single nucleotide polymorphism) as instrumental variables (IVs) to infer causal relationships between exposures and outcomes [1]. It is based on the stability of genes and Mendel's first and second laws of inheritance [2]. The way genes are allocated determines that the relationship between genes and outcomes is not affected by postnatal environmental, behavioral, socioeconomic, and other confounding factors. Therefore, the causal relationships derived from MR studies are more reasonable and reliable. MR has evolved significantly since its inception. Early MR studies were generally limited by small sample sizes and involved few IVs, resulting in relatively low statistical power. However, with the exponential growth in the number of genome-wide association studies (GWAS) conducted globally, the summary data of tens of millions of relationships between exposures, diseases, and genetic variants have been successively released, continuously increasing the power of MR studies and significantly improving their accuracy.

Compared with traditional observational studies and randomized controlled trials (RCTs), MR studies have more advantages [3]. Observational studies are generally used to assess the causal relationship between exposures and outcomes [4], and RCTs also provide high-level evidence for causal relationship testing [5]. However, due to the need for strict quality control, comprehensive design, long-term follow-up, multi-effect interventions, ethical issues, and compliance, observational studies or RCTs to elucidate disease outcomes are often not feasible. Observational studies or RCTs have difficulties in controlling all potential confounding factors, while MR studies can more effectively avoid these factors. Moreover, by utilizing the inherent characteristics of genetic variants, MR studies can address the common problem of reverse causality in observational studies and provide more reliable causal inference. For instance, MR suggests there is a causal relationship between gut microbiota and delirium [6]; Nahid et al. used MR method to study the association between cathepsins and lung cancer and indicate that elevated cathepsin H levels increase the overall risk of lung cancer, adenocarcinoma, and lung cancer among smokers [7]; Ye et al. performed a two-sample MR analysis to estimate the causal effect of mental well-being, and some mediators were identified [8]. In summary, MR has been instrumental in validating or refuting hypothesized causal relationships in various fields, including cardiovascular diseases [9, 10, 11, 12], metabolic disorders [13, 14], psychiatric conditions [15, 16, 17] and drug discovery [18, 19, 20].

MR has emerged as a powerful approach for investigating causal relationships between exposures and outcomes using GWAS summary data. However, conducting MR analyses can be challenging due to the inconsistency of GWAS data format, the complexity of different methods, the lack of standardized workflows, and the need for extensive coding experience to complete the entire process, which can lead to unreliable results. Meanwhile, the lack of standardization of workflows not only affects the reliability of the results, but also hinders the reproducibility of MR studies across different research groups. Existing tools for handling or visualizing GWAS datasets and conducting MR analysis are mostly implemented in R software and focus on specific functionalities, such as specific MR approaches, data munging, or plotting. The fragmentation of workflows further complicates the MR analysis process, as researchers must navigate multiple packages or tools and integrate them into a coherent workflow. These tools also often lack user-friendly interfaces, making them inaccessible to researchers without extensive programming skills. Furthermore, data preprocessing, a crucial and fundamental step in MR analysis, remains a significant hurdle, hindering the widespread adoption of MR in genetic epidemiology investigations. The lack of standardization in

GWAS summary data formats across different databases or consortiums complicates the usage of these data. Despite efforts to develop a standard GWAS format, the large number of existing unprocessed GWAS summary data remains a challenge for data sharing and efficient reuse. It is therefore vital that we can ensure consistency across these datasets to minimize the risk of analytical mistakes due to user error. One such inconsistency is the naming of the effect allele and non-effect allele in these datasets [21]. Besides, the missingness of certain information (such as rs ID) and certain value conversions like -log10 transformation of *P*-values can also hinder the direct reuse of these GWAS data, especially for beginners, and are error-prone during data and format conversions without careful reading manuals. Moreover, the conversion of SNP identifiers, specifically from CHR:POS:REF:ALT (chromosome, base pair location, non-effect allele, and effect allele) to rs IDs, is often time-consuming and inefficient with existing tools, and the need for strong programming skills to use command-line tools, coupled with the complex installation and deployment processes, further impedes the broader adoption of MR in genetic epidemiology studies.

To address these challenges in MR analyses and make them more accessible, reliable, and reproducible, we developed MRanalysis, an integrated, versatile and comprehensive web-based platform for MR as well as some post-GWAS/MR analyses, and GWASkit, a standalone tool for rapid GWAS data preprocessing, ensures seamless compatibility with a wide range of other post-GWAS tools and applications in our platform through functions such as rs ID conversion, data format standardization, data extraction. Our aim was to create an intuitive, efficient, and user-friendly toolset that streamlines the entire MR workflows while enhancing accessibility for researchers across disciplines. By incorporating the guidelines proposed by Burgess et al. [22], we standardized MR analyses and ensured adherence to best practices, improving the quality and reproducibility of MR studies. The combination of these tools provides a comprehensive solution for handling and visualizing GWAS data, performing various MR analyses, and lowering the barrier to perform MR analyses. By streamlining the MR workflows, promoting best practices, and supporting various MR methodologies, including univariable, multivariable, and mediation MR analyses, our platform has the potential to accelerate MR studies and contribute to a better understanding of causal relationships in complex biological systems.

MRanalysis and GWASkit, as a zero-code platform and tool, meet the needs of kinds of researchers with limited programming experience, lowering the barrier to performing MR analyses. Through the powerful interactive capabilities of the MRanalysis platform, users can perform personalized analyses and visualizations. The platform also provides code generation functionality, assembling code based on the current user-set parameters. This offers further customization and result reproduction for users with some coding ability, allowing them to understand all the details of the entire analysis. Simultaneously, the powerful functionalities of GWASkit facilitate a wide range of post-GWAS analyses, greatly enhancing genetic research using GWAS summary data. To demonstrate the utility, practicality, convenience and reproducibility of our platform and tool, we performed several real-world case studies. The development of MRanalysis and GWASkit represents a significant stride forward in genetic epidemiology research, facilitating more comprehensive investigations into relationships between genes and various phenotypes and accelerating discoveries in genetic epidemiology and drug discovery, ultimately leading to improved public health strategies and more targeted clinical interventions. Such a platform would serve as a bridge linking unprocessed GWAS summary data to post-GWAS/MR analysis tools seamlessly, enabling researchers with diverse backgrounds and varying levels of coding expertise to conduct MR studies with confidence. The increased accessibility and standardization of MR methods will not only enhance the reliability and reproducibility of MR studies but also foster collaboration and knowledge exchange among researchers from different disciplines.

Materials and Methods

Data sources

GWAS summary data regarding the data sources and sample sizes used in this study is outlined in Table 1. All cases utilized publicly available summary-level GWAS data from participants of European descent and no specific ethical approval was necessary for conducting this study. The GWAS summary data for major depression disorder (MDD) is a genome-wide summary statistics from a meta-analysis of 33 cohorts of Psychiatric Genomics Consortium (PGC) (excluding UK BioBank and 23andMe data) was described in Wray et al. [23]. The total number of individuals in this data is 500,199 (329,443 controls and 170,756 cases) with 8,483,301 variants analyzed. The frailty index (FI), derived from the cumulative defect model, served as a metric for assessing frailty severity [24] (sample size is 175,226). Each individual's FI was calculated by dividing the number of defects by the total 49 defects. Individuals received a score of 0 or 1 based on the presence of defects (0 indicating none. A higher FI value indicated greater frailty. Other GWAS data were obtained from IEU OpenGWAS directly. For more detailed information, please refer to Table 1.

rs ID mapping

We evaluated the performance of five locally installed tools, namely GWASkit v1.0.0, ANNOVAR v2020-06-07 (latest version), snpEff v5.2c, MungeSumstats v1.10.1, and gwaslab v3.4.48, along with two web-based tools, the NCBI dbSNP database and SNPnexus, for their ability to handle rs ID conversion tasks. To ensure a comprehensive and concrete assessment, we utilized a large-scale GWAS data (GCST90236305 [31]) downloaded from the EMBL-EBI GWAS Catalog, which contained 14,519,897 variants with complete information, including chromosome, base pair location, other allele (non-effect allele), effect allele, and rs ID, making it an ideal test data. During the evaluation process, we maintained the default parameters and employed the latest default reference data for each of the five local tools to ensure a fair comparison.

The primary testing environment is in a Linux® Ubuntu 20.04.4 LTS operating system, equipped with a 16-core Intel® (CPU), 128GB of RAM, and a 12TB hard drive. Besides, to assess the cross-platform compatibility and the performance of our GWASkit, we additionally tested it on a Windows operating system featuring 16GB of memory, and Intel® 4-core i7-8650U (CPU) with a 1TB hard drive.

GWAS summary data standardization

The GWAS summary data for major depression disorder (MDD) and frailty index (FI) were downloaded from DataShare and GWAS Catalog (above data sources session, Table 1), respectively. However, these two data were not in the standard VCF format, and the MDD GWAS data only contained rs ID without essential chromosome and position information. To address this issue, we utilized the GWASkit rs2pos (rs2pos -I PGC_UKB_depression_genome-wide.txt -O PGC_UKB_depression.tsv.gz –rs MarkerName –rsdb /rsdb/GRCh37 –SEP 1 –rm -V -Z) command to annotate the rs IDs with chromosome number, base-pair position, non-effect allele and effect allele information. Subsequently, we performed the tsv2vcf command (GWASkit tsv2vcf -I PGC_UKB_depression.tsv.gz -O PGC_UKB_depression.vcf.gz –TYPE GRCh37 –CHR CHR –POS POS –REF A2 –ALT A1 –RSID SNP –BETA LogOR –EAF Freq –SE StdErrLogOR –PVALUE P –SS 500199 -V) to standardize

Table 1. GWAS data sources included in the case study

Phenotype	ID[a]	Sample size (overall or case/control)	Consortium or Author	PMID
MDD[a]	ieu-b-102	170,756/329,443	PGC, UK Biobank	30718901, Howard et al. [25]
frailty index	ebi-a-GCST90020053	175,226	UK Biobank, TwinGene	34431594, Atkins et al. [24]
HDL cholesterol	ieu-a-299	187,167	GLGC	24097068, Gustafsson et al. [26]
LDL cholesterol	ieu-a-300	173,082	GLGC	24097068, Gustafsson et al. [26]
Triglyceride	ieu-a-302	177,861	GLGC	24097068, Gustafsson et al. [26]
Creatinine	met-d-Creatinine	110,058	Borges CM	-
coronary heart disease	ieu-a-7	60,801/123,504	CARDIoGRAMplusC4D	26343387, Nikpay et al. [27]
feeling lonely	ebi-a-GCST006942	376,352	Mats N	29500382, Nagel et al. [28]
MDD[c]	ebi-a-GCST009981	9,487/39,677	Jonathan R I C	31969693, Coleman et al. [29]
cigarettes smoked per day	ieu-b-142	249,752	GSCAN	30643251, Liu et al. [30]

PMID: PubMed ID; PGC: Psychiatric Genomics Consortium; HDL: high-density lipoprotein; LDL: low-density lipoprotein.
a. ID in IEU OpenGWAS; b. major depression disorder; c. major depressive disorder in trauma-unexposed individuals.

the GWAS statistics and generate VCF file in standard format, which can be directly uploaded to the MRanalysis online platform for performing MR analysis. In the case of the FI GWAS data, since it already contained all the necessary information for MR analysis, we only need to directly apply the tsv2vcf command for data standardization (GWASkit tsv2vcf -I 34431594-GCST90020053-EFO_0009885.h.tsv.gz –CHR chromosome –POS base_pair_location –REF other_allele –ALT effect_allele –RSID variant_id –BETA variant_id –EAF effect_allele_frequency –SE standard_error –PVALUE p_value –TYPE GRCh37 –SS 175226 -O GCST90020053.vcf.gz -V).

It is worth noting that all the above-mentioned annotation and standardization operations can also be performed using the Windows version of GWASkit. The detailed process for using GWASkit on Windows can be found in the GWASkit help documentation, which is available on GitHub. By leveraging the powerful features of GWASkit, researchers can convert different kinds of formats of GWAS summary statistics into a standard one, enabling seamless integration with the MRanalysis platform for conducting MR analysis. This streamlined workflow not only saves time and effort but also ensures the accuracy and reliability of the results obtained from the MR analysis.

GWAS summary data quality control

MRanalysis is also a convenient platform for processing quality control (QC) of GWAS statistics, integrating three main functionalities: CheckSumStats, Quantile-Quantile (Q-Q) plot, and Manhattan plot.

CheckSumStats is an R package developed by Haycock et al. [32] that provides a quality control pipeline to identify potential metadata errors, summary data issues, and other analytical problems in GWAS results. These errors and issues can introduce substantial bias into downstream analyses, such as two-sample MR studies. CheckSumStats leverages three groups of SNPs to perform its check: a 1000 Genomes reference set, GWAS catalog associations, and the test GWAS top hits. By extracting summary data for these SNP groups from the target GWAS, CheckSumStats can confirm the identity of the effect allele frequency and effect allele columns, identify errors or analytical issues in the summary dataset, and infer the study's ancestry. The package aims to enhance the integrity of collated summary data prior to analysis, thereby increasing the reliability of post-GWAS analyses. To make CheckSumStats more accessible to researchers, we developed a web-based application that allows users to directly upload their data for quality control analysis easily.

The Q-Q plot is a graphical representation of the deviation of the observed *P*-values from the null hypothesis: the observed *P*-values for each SNP are sorted from largest to smallest and plotted against expected values from a theoretical χ^2-distribution. Additionally,

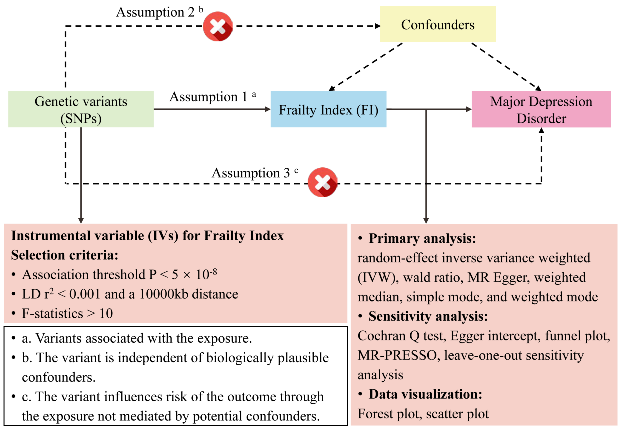

Figure 1. Workflow of the current two-sample Mendelian randomization (MR) study revealing causality from frailty index on major depression disorder. SNPs, single nucleotide polymorphisms; LD, linkage disequilibrium, MR-PRESSO, MR pleiotropy residual sum and outlier.

a Manhattan plot represents the *P*-values of the entire GWAS on a genomic scale, and it is normally used to check for consistency and to identify spurious associations. In a Manhattan plot, the *P*-values are represented in genomic order by chromosome and position on the chromosome (x-axis). The value on the y-axis represents the -log10 of the *P*-value. In this case, we also use Q-Q plot and Manhattan plot applications in MRanalysis to perform these two analyses directly.

Two-sample Mendelian randomization

To validate the accuracy of our platform, we replicated the findings of causality between major depression disorder (MDD) and frailty index (FI) from Wang et al. [33] using our online platform. To investigate the causal relationship between MDD and FI, we obtained GWAS summary statistics for MDD (n = 500,199) and FI (n = 175,226) from previously published studies, serving as exposure and outcome datasets, respectively (Table 1). The SNPs associated with the exposures at genome-wide significance ($P < 5 \times 10^{-8}$) were selected as instrumental variables (IVs) with F-statistics all greater than 10, satisfying MR assumptions (Figure 1). To ensure the independence of these IVs, we pruned them for linkage disequilibrium (LD) using LD clumping (r^2 < 0.001, distance = 10,000 kb).

In the MR analysis, we employed the random-effect inverse variance weighted (IVW) method as the primary analysis to study the causality between MDD and FI. We performed the Cochran's

Q test to assess the heterogeneity. To evaluate the robustness of the MR estimates, we compared the IVW approach with other MR methods, including Wald ratio, simple mode, MR Egger, weighted median, and weighted mode. We also utilized the intercept term derived from MR-Egger and MR-PRESSO to assess the horizontal pleiotropy. Leave-one-out analysis was also conducted to evaluate the sensitivity of MR results.

All the aforementioned steps of two-sample MR analysis can be performed online using our platform (both API and local modes) with default parameters. When removing confounding factors, we referred to the article by Wang et al. [33]. By leveraging our online platform, researchers can conveniently and without coding conduct two-sample MR analyses to explore the causal relationships between complex traits and diseases, providing important theoretical foundations and practical guidance for the development of disease prevention and treatment strategies.

All MR analyses in our platform were conducted using "TwoSampleMR" (version 0.6.8) and "MendelianRandomization" (version 0.9.0) packages in R software (version 4.4.1).

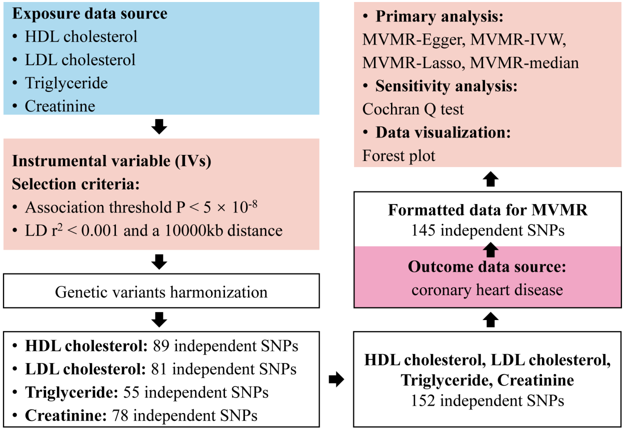

Figure 2. Workflow of the current Multivariable Mendelian randomization (MVMR) study revealing causality from HDL cholesterol, LDL cholesterol, Triglyceride, and Creatinine on coronary disease. SNPs, single nucleotide polymorphisms; LD, linkage disequilibrium; HDL, high-density lipoprotein; LDL, low-density lipoprotein; IVW, inverse variance weighted.

Multivariable Mendelian randomization

Mendelian randomization (MR) is a powerful approach for inferring causal relationships between exposures and outcomes using genetic variants (SNPs) as instrumental variables (IVs). It can be conducted using either individual-level data or summary data from GWAS, which provide the estimated effect of each SNP of exposure on the outcome. Multivariable Mendelian randomization (MVMR) extends the traditional MR framework by allowing for the estimation of causal effects of multiple exposures on the outcome, conditional on the other exposures included in the model. MVMR also can be used to evaluate mediating effects of an independent variable, to adjust for possible pleiotropy bias due to horizontal pleiotropy of a specific effect, or to adjust for potential confounding.

In this case, as shown in Figure 2, we performed a two-sample MVMR analysis using summary dataset from GWAS of HDL cholesterol, LDL cholesterol, Triglyceride, and Creatinine as exposures, and coronary heart disease (CHD) as outcome (Table 1). We selected SNPs that reached genome-wide significance ($P < 5 \times 10^{-8}$) in at least one of the exposure traits and pruned them for linkage disequilibrium (LD) using a pairwise r^2 threshold of 0.001 and 10,000kb distance. The resulting set of independent SNPs was then used as IVs in the MVMR analysis. We employed several MVMR methods, including multivariable MR-Egger, multivariable IVW, multivariable MR-Lasso, and multivariable median-based approaches, to estimate the causal effects of the exposures on the outcome. These analyses were conducted using the "MVMR" (version 0.4) and "MendelianRandomization" packages in R.

To assess the strength and validity of the IVs in the two-sample data, we used Cochran's Q statistical test to evaluate the robustness of results and ensure that the assumptions of MR were satisfied. By leveraging the power of MVMR and the wealth of summary data from large-scale GWAS, our applications provide valuable insights into the complex causal relationships between multiple exposures and the outcome of interest. The use of multiple MVMR methods and the assessment of IVs' strength and validity further strengthen the reliability of our tools.

Two-step or mediation Mendelian randomization

Mediation analysis is a powerful approach to study the underlying mechanisms through which an exposure affects an outcome. In the context of Mendelian randomization (MR), a two-step MR analysis can be employed to assess the potential role of a third variable (mediator) in the causal pathway between exposure and outcome. In this application the first step involves using genetic instrumental variables (IVs) associated with the exposure to determine the causal effect of the exposure on the potential mediator. The second step then utilizes IVs associated with the potential mediator, independent of those used in step one, to estimate the effect of the mediator on the outcome of interest. Methods such as the product-of-coefficients can be applied to quantify the extent of mediation. Importantly, the MR assumptions must be satisfied for both steps of the analysis: (i) exposure on mediator and (ii) mediator on outcome.

In this case, we conducted a four-step two-sample MR analysis to evaluate the complex relationships among feeling lonely, major depression disorder in trauma-unexposed individuals (MDD), and cigarettes smoked per day (Table 1). Step 1 involved an MR analysis of feeling lonely on MDD, while step 2 examined the reverse causal relationship between feeling lonely and MDD. These two steps were combined into a bidirectional MR analysis to explore the potential primary and reverse causal relationships between feeling lonely and MDD. Step 1, 3, and 4 were then integrated into a two-step MR mediation analysis to assess the potential mediating role of cigarettes smoked per day in the relationship between feeling lonely and MDD. Specifically, step 3 investigated the causal effect of feeling lonely on cigarettes smoked per day, and step 4 evaluated the causal effect of cigarettes smoked per day on MDD. In this mediation analysis, beta0 represents the total effect, while beta1 and beta2 represent the direct effects of feeling lonely on cigarettes smoked per day and cigarettes smoked per day on MDD, respectively (Figure 3). The specific calculation method (product of coefficients) for the mediating effect is also shown in Figure 3.

In the first step, we evaluated the causal relationship between exposure and mediation variables (beta1). Subsequently, in the second step, we estimated the causal effect of mediators on outcomes through multivariate MR (beta2). We then calculated the total effect (beta0) between exposure and outcome using two-sample MR analysis. When beta0, beta1, and beta2 were all significant, a causal relationship existed between the outcome and exposure, and the mediating variable played a partial mediational role in this causal relationship. The mediating effect was calculated using beta1*beta2, while the mediating proportion of the causal effect between exposure and outcome was calculated using (beta1*beta2)/beta0. Finally, we estimated the proportion of the mediation effect in the total effect using the delta method. We calculated the odds ratios (OR) and 95

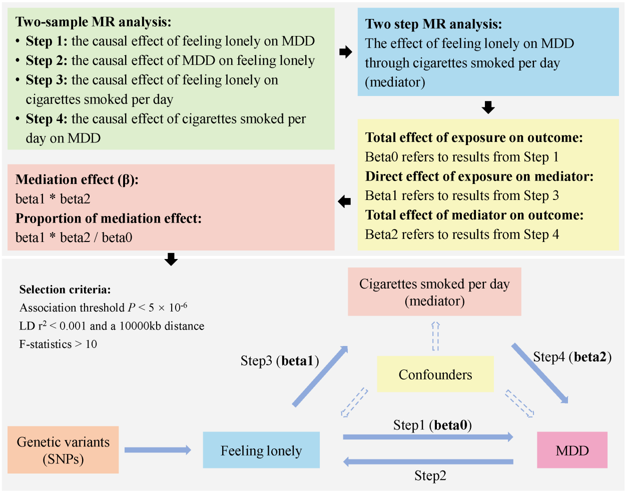

Figure 3. Flowchart of two-step Mendelian randomization analysis revealing the mediating effect of cigarettes smoked per day on the risk of MDD through feeling lonely. MR, Mendelian randomization; MDD, major depressive disorder in trauma-unexposed individuals; SNPs, single nucleotide polymorphisms.

SNP gene mapping and enrichment

Gene and gene-set analysis are powerful statistical approaches that enable researchers to investigate the combined effects of multiple genetic markers on complex, polygenic traits. These methods are particularly useful when individual markers have weak effects that are difficult to detect using traditional single-marker analysis. Furthermore, gene-set analysis can provide valuable insights into the functional and biological mechanisms underlying the genetic component of a trait. While several methods for gene and gene-set analysis are available, they often suffer from various statistical issues and can be computationally intensive. To address these challenges, De Leeuw et al. [34] have developed a new method called MAGMA, which offers improved statistical power and computational efficiency compared to existing methods. To make MAGMA more accessible and user-friendly, we have developed a Shiny application that allows users to directly upload their data for this analysis. This application streamlines the process of conducting gene and gene-set analysis, making it easier for researchers to explore the genetic basis of complex traits.

The genes identified through the aforementioned mapping process were subsequently analyzed using our MRanalysis platform. The platform conducted Gene Ontology (GO) and Kyoto Encyclopedia of Genes and Genomes (KEGG) enrichment analyses based on the clusterProfiler (v4.12.6) R package [35], with default parameters applied.

Power and sample size estimation

For power and sample size estimation, we used the PowerCalculator and SampleSizeCalculator applications integrated within the MRanalysis platform to determine statistical power and the minimum required sample size for three types of MR analyses: two-sample univariable MR (2SMR), multivariable MR (MVMR), and mediation MR (MMR). Because the tools do not directly support power and sample size calculations for MVMR and MMR, we decomposed each multivariable or mediation analysis into exposure–outcome components and estimated sample sizes separately. For each computation, only one of "sample size" or "power" was specified and the other was computed by the tool. In Case VIII, we applied the MR analyses described above and, for each exposure–outcome pair, computed the minimum required sample size individually. The minimum detectable effect size with the current samples, assuming 80% power and a two-sided significance level of 0.05, was obtained using the SampleSizeCalculator application, which implements code from Burgess [36]. Required inputs included the proportion of variance in the exposure explained by the selected genetic instruments (R^2), the anticipated causal effect size (odds ratio per standard deviation for binary outcomes or standard deviation change per standard deviation for continuous outcomes), the significance level (α), and, for binary outcomes, the case-to-control ratio. For two-sample analyses, the sample size was defined as the number of participants in the variant–outcome association dataset.

Results

Overall design and workflow of MRanalysis

MRanalysis is a comprehensive web-based platform designed to streamline and standardize Mendelian Randomization (MR) analysis and preprocess GWAS summary dataset. By leveraging the extensive interactive features of the R Shiny framework, MRanalysis provides a wide range of interactive functionalities, from handling various GWAS data file formats (e.g., CSV, TSV, VCF) to performing data extraction, format standardization, rs ID mapping, gene mapping, enrichment analysis, data visualization, and supporting several common MR approaches. To enhance user experience and facilitate easier adoption of our platform, we have prepared sample datasets for each application. These example datasets allow users to familiarize themselves with the platform's functionalities and test its features before using their own data. Moreover, we have created comprehensive animated GIFs for each application, visually illustrating the key steps of the process. These animated guides provide a clear, step-by-step visual representation of how to navigate the platform and utilize its various tools. The overall structure of our platform can be divided into three main components: the analysis section (containing common MR approaches), the plugin section (encompassing various post-GWAS/MR methods), and the visualization section. The analysis section is the core component of MRanalysis, where researchers can conveniently perform univariable, multivariable, and mediation MR in both local and API modes.

When users want to perform an MR analysis, the overall pipeline can be divided into 5 main stages: data preprocessing, quality control (QC), MR analysis, post-MR analysis and data visualization (Figure 4). Taking local two-sample MR analysis (univariable) as an example, (1) in the data preprocessing stage, users can utilize the GWASkit standalone tool to efficiently handle tasks such as format conversion (e.g., VCF to TSV, or TSV to VCF), rs ID mapping (converting chromosome, base pair location, effect and non-effect allele information to SNP IDs), and GWAS data format standardization. GWASkit's "pos2rs" and "rs2pos" subcommands specifically cater to mapping between rs IDs and their coordinates, offering advantages of being pre-installed, multi-platform, efficient, highly accurate, and fast compared to other existing tools. (2) The QC stage is crucial before conducting MR analyses. Our platform provides three key functions: CheckSumStats for identifying allele frequency conflicts and metadata errors in GWAS datasets, Manhattan plot for visualizing genetic associations and significance levels for multiple SNPs, and Q-Q plot for assessing the distribution and overall characteristics of GWAS data. These features ensure the quality and reliability of the data before proceeding with MR analyses. (3) In the analysis stage, MRanalysis offers three mainstream MR approaches, including Two-Sample univariable MR, Multivariable MR, and Mediation MR, through its "Analysis" module and associated plugins. Users can easily conduct these analyses using either API mode for searching GWAS data online or local mode for uploading their own GWAS data. (4) After MR analysis, we can annotate instrumental variables and conduct gene enrichment analyses, including GO and KEGG. This process enhances biological interpretation of MR results, potentially revealing functional pathways underlying causal

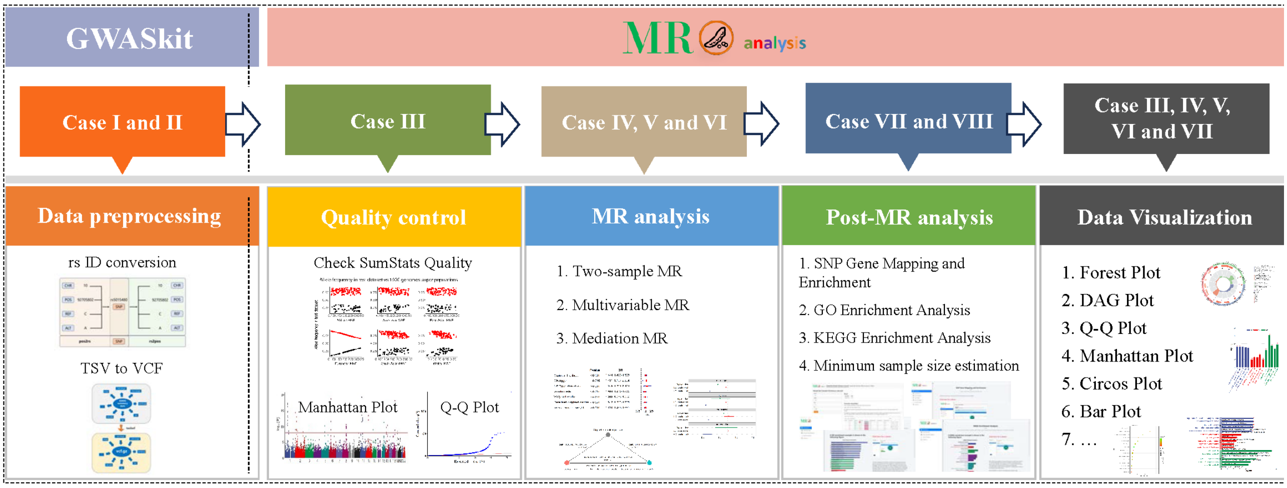

Figure 4. Overview of MRanalysis design.

relationships. (5) At last, the results can be visualized using our plot applications, such as, forest plot, DAGs (Directed Acyclic Graphs), bar plot, dot plot and circos plot to facilitate intuitive interpretation and presentation of the findings.

To further enhance the robustness and reliability of MR analyses, MRanalysis also provides several additional utilities, including a Power Calculator application for estimating the statistical power of MR studies and a Sample Size Calculator application for determining the minimum sample size required for a given power [37]. The power of an MR study is determined by the sample size and the strength of the association between the proposed instruments and the risk factor [38]. Users can also perform SNP to gene annotation and enrichment analysis using the corresponding plugins (e.g., MAGMA application [34] within our platform directly.

In summary, MRanalysis coupled with the GWASkit, presents a complete and user-centric solution for researchers to efficiently handle GWAS data preprocessing, QC, MR analyses and visualization, as well as perform enrichment analyses. The platform's modular structure and extensive capabilities render it a crucial resource for researchers, particularly those with limited coding skills, to address the intricacies of MR analysis and elucidate causal relationships between genetic variants and phenotypes of interest. It is worth noting that each application within the platform provides complete code generation capabilities, enabling local reproduction of results. Through this code, users can clearly understand the entire analysis process and support further personalized analyses. The seamless integration of the three main sections within MRanalysis highlights its value and utility in MR, providing an accessible, versatile, and potent platform for scientists to overcome the challenges associated with GWAS data processing and MR studies.

Case I: rs ID mapping

Single nucleotide polymorphisms (SNPs) are genomic locations known to vary between individuals. The rs ID number is a unique identifier ("rs" followed by a number, e.g. rs12306) used by researchers and databases to designate a specific SNP. This naming convention, which stands for Reference SNP cluster ID, is widely used for most SNPs. When researchers identify a SNP, they send a report containing the sequence surrounding the SNP to the dbSNP database. Submitted variants are categorized, organized, and annotated, with duplicate variants being consolidated. Unlike the CHR-POS identifier, which changes with different reference genome versions while rs ID remains consistent across versions. This consistency provides a stable method for representing variants, making it more suitable for large-scale studies in population genetics or precision medicine.

GWASkit pos2rs provides functions to convert CHR-POS-REF-ALT (chromosome, base pair locations, non-effect (other or reference) allele and effect allele) to rs ID using reference files downloaded from the NCBI dbSNP database, or self-prepared files for rapid conversion.

To evaluate the performance of rs ID conversion by GWASkit and other existing tools, we used a GWAS summary data downloaded from the GWAS Catalog as test data. Figure 5A illustrates the run time for rs ID conversion using GWASkit and six other existing tools (ANNOVAR [39], snpEff [40], MungeSumstats [41], gwaslab [42], NCBI dbSNP database and SNPnexus [43]). In comparison, GWASkit required the shortest time to complete the rs ID conversion, taking only 0.24 hours (14.40 minutes), while ANNOVAR, MungeSumstats, and gwaslab require 0.42, 0.38, and 8.01 hours, respectively (Figure 5A). Meanwhile, snpEff required the longest time, 20.67 hours. It is important to note that snpEff is a genetic variant annotation and functional effect prediction toolbox, which simultaneously annotates many other pieces of information, resulting in a more time-consuming process. In terms of RAM usage, ANNOVAR and snpEff required similar amounts of memory consumption, but much less than MungeSumstats (Figure 5B). The high memory requirement of MungeSumstats is likely due to the R compiler's tendency to use more memory. In comparison, GWASkit uses a moderate amount of memory, 22.00GB, primarily to achieve better performance. We also tested GWASkit on a Windows computer with 8GB of available memory, and it worked normally, although the runtime was extended by approximately 1 hour.

When comparing different tools from various perspectives (Table 2), we find that the choice of tool should be based on specific usage scenarios. For batch processing needs, local tools are more recommended due to their efficient processing capabilities; conversely, if only several SNPs need to be annotated, direct use of some online tools, such as the NCBI dbSNP database or SNPnexus, is sufficiently convenient. Considering that GWAS summary data is usually very large, often reaching hundreds of megabytes (MB) or even 1 or 2 gigabytes (GB), the advantages of local tools for processing such large data are particularly evident (Figure 5). It is worth noting that most of these tools are developed based on the Linux operating system or require certain programming skills, posing some requirements for users. From the user's perspective, accuracy is the primary indicator for evaluating tool performance, while the simplicity of tool installation and ease of use are also important, as they directly set a threshold that limits more researchers from using it. Regarding accuracy, our tool GWASkit significantly outperforms other tools with its extremely high accuracy rate (99.999993%, close to 100%, Table 2), followed by ANNOVAR and snpEff with

Table 2. Feature comparisons of currently available rs id conversion tools (local and web tools)

Tool	Type	Platforms	Standalone	User Interface	Batch	Accuracy(%)
GWASkit	local	Linux, Windows	Yes	UI for Windows	Yes	100.0
ANNOVAR	local	Linux	Perl Scripts	No	Yes	97.25
snpEff	local	Linux	JAR file	No	Yes	98.87
MungeSumstats	local	Linux, Windows	R package	No	Yes	59.67
gwaslab	local	Linux, Windows	Python module	No	Yes	98.35
NCBI dbSNP	online	-	-	Yes	No	-
SNPnexus	online	-	-	Yes	Yes[a]	-

a. SNPnexus limits the maximum number of variants in a single batch query to 10,000. UI: user interface.

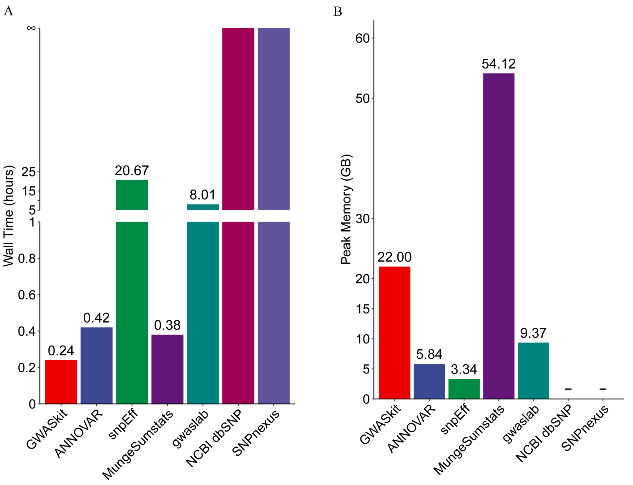

Figure 5. Run time (A) and peak memory consumption (B) by different tools. Test data: a large-scale GWAS summary data (GCST90236305) downloaded from the EMBL-EBI GWAS Catalog database, which contained 14,519,897 variants with complete information, including chromosome, location, other allele, effect allele, and rs ID details. Test environment: Linux® Ubuntu 20.04.4 LTS operating system, equipped with a 16-core Intel® (CPU), 128GB of RAM, and a 12TB hard drive.

accuracy rates of 97.25% and 98.87%, respectively. In contrast, MungeSumstats has the lowest accuracy rate of only 59.67%, which somewhat limits its application in high-accuracy scenarios.

Overall, GWASkit not only stands out with its excellent accuracy but is also particularly suitable for researchers with limited or even no programming skills. GWASkit is a standalone binary executable file that avoids complex installation steps and supports multiple operating systems (Table 2). Especially with its Windows version, which features a simple user interface (UI), users can complete tasks that typically require programming skills and complex installation by just clicking and entering necessary information. This greatly lowers the usage threshold and enhances efficiency. Additionally, using GWASkit for rs ID conversion can be done with just a single command.

Case II: GWAS summary data standardization

The VCF (Variant Call Format) is a standard text file format widely used in bioinformatics for storing gene sequence variations. VCF files facilitate the integration of GWAS summary data with other genomic datasets by providing metrics and filters that ensure only reliable variants are considered. The standardized format allows them to be used across different tools and platforms, making VCF files a versatile choice for researchers. GWASkit provides functions for standardizing the format of GWAS summary data, ensuring that datasets from a wide range of sources are as interoperable as possible. Most importantly, for local MR analysis applications (e.g, Two-sample MR analysis), reading a large-scale GWAS summary data with R scripts typically requires a significant amount of memory and is time-consuming. For example, processing a 500MB GWAS summary data locally might use 8 to 10GB of memory and take about 20 minutes. To address this, MRanalysis has optimized the process by using standard VCF format files for local analysis, reducing the time to just 2 to 3 minutes and requiring only 1 to 2 GB of memory.

To illustrate the versatility of GWASkit in converting GWAS statistics from various formats into the standardized VCF format, we provided a comprehensive walkthrough using the MDD and FI data from Table 1. The MDD data posed a challenge, as it only included rs ID information, lacking some crucial details such as chromosome number and base pair location. To address this issue, we employed the "rs2pos" subcommand of GWASkit, which efficiently filled the missing information. The process took approximately 12.93 minutes and had a peak memory usage of 17.79GB. Upon completion of the data augmentation, we proceeded to utilize the 'tsv2vcf' subcommand to perform a standardization of the data. This step required 3.27 minutes and had a peak memory usage of 6.18GB. In contrast, the FI dataset already contained all the necessary information, allowing us to directly apply the "tsv2vcf" for standardization, which also took about 3 minutes and had a peak memory usage of 7.33GB. By successfully converting the MDD and FI GWAS data into standardized VCF format, we prepared them for subsequent MR analysis.

GWASkit's robust data processing and format conversion capabilities offer researchers an efficient and user-friendly tool to tackle GWAS data in various formats from various databases. The streamlined workflow provided by GWSAkit, from data augmentation to standardization, not only saves time and computational resources but also ensures data consistency and compatibility across different platforms. This standardization is particularly crucial for large-scale meta-analysis and collaborative research efforts, where data from multiple sources need to be integrated and analyzed together. Moreover, the reduced memory usage and processing time achieved by MRanalysis using standardized VCF files significantly enhance the accessibility and feasibility of local MR analyses. This optimization enables researchers with limited computational resources to perform complex analyses on their own machines.

Case III: GWAS summary data quality control

The comparison of allele frequencies between the frailty index (FI) GWAS dataset and the 1000 Genomes reference population data allows for a systematic examination of the accuracy and consistency of the genetic data, establishing a solid foundation for subsequent genetic statistical analyses. In this case, we used the CheckSumStats application to intuitively present the distribution patterns of SNP allele frequencies in the two datasets. As shown in Figure 6A, black points represent SNP loci with consistent frequencies, while red points represent SNP loci with frequency conflicts. The vast majority of SNPs have frequencies less than 0.5 and are consistent between the two datasets, suggesting that the reported effect allele

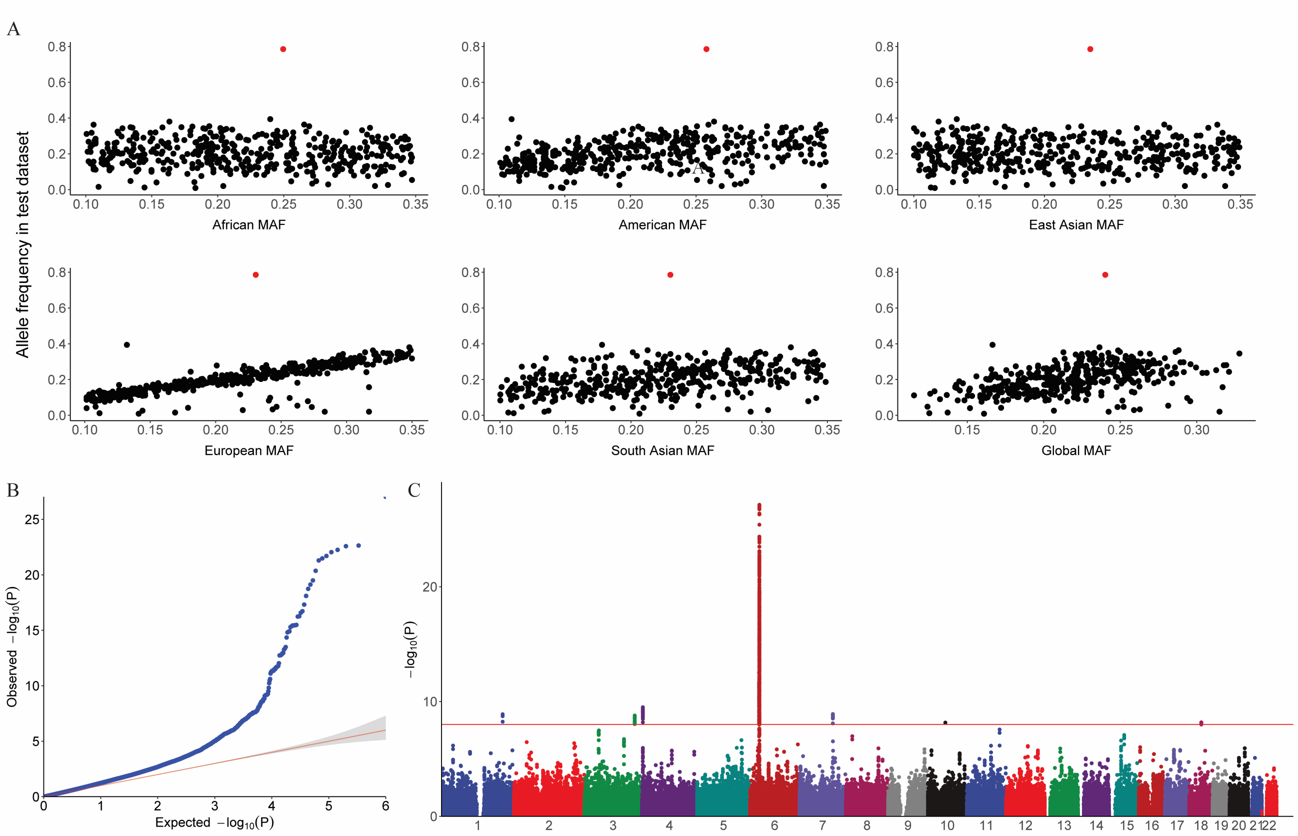

Figure 6. Quality control of GWAS summary data of frailty index. (A) Allele frequency in test dataset (frailty index) vs 1000 genomes super populations. Allele frequencies are expected to be < 0.50 (black points). A high allele frequency conflict is defined as an allele frequency of > 0.58 (red points) in the test data or if the allele frequency differs by > 10 points between the test and reference data sets. Moderate allele frequency conflicts are allele frequencies of > 0.50 but $\leq$ 0.58; (B) Q-Q plot of the GWAS dataset. The observed results deviate significantly from the expected results, suggesting that the effects of these loci exceed random effects and may be significantly associated with the phenotype; (C) Manhattan plot of the GWAS dataset. The horizontal axis represents the genomic locations of all the tested SNP in physical order. The vertical axis shows -10log *P* values for individual variant association with frailty index. Red lines indicate the threshold for genome-wide significance at 5×10^{-8}.

frequencies in this case dataset can accurately correspond to the effect alleles themselves. However, a considerable proportion of SNPs exhibit frequency conflicts, and the speculated reason may be that the effect allele frequency column confuses effect alleles and non-effect alleles, actually recording the minor allele frequency. The identification of allele frequency conflicts underscores the importance of careful data curation and quality control in genetic association studies. Misclassification of effect and non-effect alleles can introduce biases and lead to erroneous conclusions. Therefore, it is crucial to implement robust data cleaning and validation procedures to ensure the accuracy and reliability of the dataset before conducting downstream analyses.

The Q-Q plot (Figure 6B) showed that the observed results deviated significantly from the expected results at lower *P*-values, suggesting that the effect of these loci exceeds random effects and might be significantly associated with the phenotype. This deviation from the expected distribution indicates the presence of true associations between the genetic variants and the FI, warranting further investigation into the biological mechanism underlying these associations. The Manhattan plot (Figure 6C) revealed that a SNP cluster on chromosome 6 showed the most significant association with the FI. This finding highlights the potential importance of genetic variants in this region in influencing an individual's susceptibility to frailty. In conclusion, the CheckSumStats, in conjunction with the Q-Q plot and Manhattan plot, provides a comprehensive assessment of the GWAS datasets.

Case IV: Two-sample Mendelian randomization

In this case, the findings of causality between major depression disorder (MDD) and frailty index (FI) of Wang et al. [33] were replicated using our web platform. Two approaches were employed: (1) using the local mode of two-sample MR analysis with standardized GWAS dataset (Case I, II and III); (2) directly utilizing the API mode application to access online data from the IEU OpenGWAS project. Remarkably, the results obtained from both approaches were nearly identical, demonstrating the robustness and reproducibility of the findings. Some minor discrepancies observed between the two approaches can likely be attributed to the personalized processing and curation steps implemented by IEU OpenGWAS during their data cleaning and preprocessing pipeline.

In this replication study, a total of 3,008 genetic variants associated with major depression disorder (MDD) reached genome-wide significance ($P < 5 \times 10^{-8}$) (Figure 7A). Of these, 50 SNPs were selected as the instrumental variables (IVs). The F-statistics for the IVs ranged from 29.7519 to 78.4487, all exceeding the threshold of 10, indicating that the IVs were not biased by weak instruments. This ensures the validity of the selected genetic variants as robust IVs of MDD in the Mendelian randomization (MR) analysis. The inverse variance weighted (IVW) analysis showed that the genetic changes in the MDD were statistically associated with an increased risk of frailty index (FI) (local mode: OR = 1.256, 95% CI: 1.192-1.323, *P* < 0.001, Figure 7B; API mode: OR = 1.256, 95% CI: 1.192-1.324, *P* < 0.001, Figure 7D), with some heterogeneities was observed among IVs (Q = 73.3864 and 74.5128 in local and API mode, respectively, and *P* < 0.001 both in two modes). The causality between MDD and FI was also further confirmed by other MR methods, including the MR

Egger, weight median, simple mode and weight mode (Figure 7B and D). The scatter plot and trend line showed the consistent trend of causal relationship between MDD and FI for all five MR methods (Figure 7C and E). To assess the presence of horizontal pleiotropy, which can bias the MR estimates, the MR Egger intercept test and MR-PRESSO distortion test were performed. Both tests showed no indication of horizontal pleiotropy (all P values greater than 0.05), supporting the validity of the MR assumptions and the reliability of the causal estimates.

The consistency of results across different data sources and processing methods highlights the reliability and validity of the MRanalysis platform in conducting MR analyses. By offering both local and API-based data integration options. MRanalysis provides researchers with the flexibility to choose the most suitable approach based on their specific requirements and data availability. This versatility ensures that researchers can conduct MR analyses using the most appropriate and up-to-date data sources while maintaining the integrity and comparability of the results.

Case V: Multivariable Mendelian randomization

In this case, we investigated the potential causal relationship between creatinine and coronary heart disease (CHD), considering the possibility that instrumental variables (IVs) for creatinine could act through lipid species like triglycerides to influence the risk of CHD. To account for this potential mediation, we constructed multivariable Mendelian randomization (MVMR) models that estimate the creatinine to CHD relationship conditioned on HDL cholesterol, LDL cholesterol, and triglycerides.

To further investigate the independent effects of creatinine and major lipid species on CHD, we screened 145 SNPs as IVs for the MVMR. The MVMR-IVW method showed that triglycerides (OR = 1.16, 95% CI: 1.04-1.30, P = 0.010) and LDL cholesterol (OR = 1.33, 95% CI: 1.21-1.45, P < 0.001) were significantly associated with the risk of CHD. In contrast, HDL cholesterol (OR = 0.93, 95% CI: 0.85-1.02, P = 0.135) and creatinine (OR = 1.06, 95% CI: 0.85-1.32, P = 0.600) were not significantly associated with the risk of CHD (Figure 8). These results suggest that higher levels of triglycerides and LDL cholesterol may causally contribute to the development of CHD, independent of the effects of creatinine and HDL cholesterol. The consistency of the results across the remaining four MVMR methods further strengthens the reliability of these findings. The agreement between different MR methods, each with its own assumptions and robustness properties, provides additional confidence in the conclusions drawn from the analysis.

Case VI: Two-step or mediation Mendelian randomization

This case employs a four-step two-sample Mendelian randomization (MR) approach to investigate the complex relationships between feeling lonely, major depressive disorder in trauma-unexposed individuals (MDD), and cigarette smoked per day. The bidirectional MR analysis provides insights into the potential causal directionality between feeling lonely and MDD, while the two-step mediation MR analysis explores the potential role of cigarettes smoked per day as mediator in this relationship. The result of inverse variance weighted (IVW) method shows that the genetic changes in the feeling lonely were statistically associated with the risk of MDD (OR = 1.67, 95% CI: 1.06-2.63, P = 0.026, Figure 9A), however the SNPs change in the MDD were not significantly associated with feeling lonely (OR = 0.99, 95% CI: 0.98-1.00, P = 0.355, Figure 9A), indicating that the causal relationship between feeling lonely and MDD may be unidirectional. In the analysis of the exposure-mediator relationship, the IVW results indicate a positive causal relationship between feeling lonely and cigarettes smoked per day (OR = 1.31, 95% CI: 1.12-1.52, P < 0.001, Figure 9A). This finding suggests that individuals who feel lonely may be more likely to engage in smoking behavior, potentially as a coping mechanism or due to shared underlying factors. Furthermore, for mediator to outcome relationship, IVW results suggest that cigarettes smoked per day significantly increase the risk of MDD (OR = 1.18, 95% CI: 1.03-1.34, P = 0.016, Figure 9A).

Based on the findings of step 1 (feeling lonely to MDD), step 3 (feeling lonely to cigarettes smoked per day) and step 4 (cigarettes smoked per day to MDD), the mediating role of cigarettes smoked per day in the relationship between feeling lonely and MDD has been demonstrated (Figure 9B). The mediating effect of cigarettes smoked per day in increasing the risk of MDD through feeling lonely was found to be statistically significant (β = 0.044; 95% CI: 0.0003-0.0868, P = 0.049). The mediation proportion, indicating the proportion of the total effect of feeling lonely on MDD that is mediated by cigarettes smoked per day, was estimated to be 8.47

Case VII: SNP gene mapping and enrichment

In case V, a multivariable Mendelian randomization analysis was used to study the potential causal effects of creatinine and major lipid species on CHD. To further study the functional implications of the genetic variants associated with CHD, the MAGMA plugin was employed to annotate the 146 instrumental variables (IVs) to their corresponding genes. This annotate resulted in a set of 99 genes. MAGMA uses MSigDB by default for enrichment analysis, while its extensibility allows seamless integration with external bioinformatics tools to further explore the functional impact of identified genes. In this case study, we leveraged MAGMA's capabilities to map SNPs to their corresponding genes. This crucial step bridges the gap between genetic variants and their potential functional impacts at the gene level. Following this mapping process, we conducted enrichment analyses using applications of GO and KEGG enrichment analysis. To enhance the interpretability and visual representation of our results, we imported the enrichment analysis outputs into the visualization module of MRanalysis. This visualization module offers a suite of powerful visualization tools, including circos plots, bar plots, and dot plots (Figure 10). These enrichment analyses provide valuable insights into the biological processes (BP), molecular functions (MF), and cellular components (CC) associated with the identified genes, as well as their involvement in various biological pathways in CHD.

MAGMA plugin is a powerful application that combines robust statistical methodology with an intuitive user interface, enabling researchers to uncover novel insights into the genetic architecture of complex traits. By integrating genetic association data with functional genomic annotations, MAGMA/GO/KEGG applications facilitate the identification of biologically meaningful gene sets and pathways, ultimately advancing our understanding of the underlying biological mechanisms. By integrating genetic association data with functional genomic annotations and enabling seamless integration with external bioinformatics tools, enrichment analysis empowers users to uncover novel insights into the underlying biological mechanisms.

Case VIII: Minimum sample size estimation

Using the MRanalysis SampleSizeCalculator, we estimated the minimum total sample size required to achieve 80% power at α = 0.05 for each exposure–outcome pair and compared these thresholds with the available GWAS outcome sample sizes. Five of nine analyses met or exceeded the predicted minimum and were therefore adequately powered at the observed effect sizes, while four were underpowered. Specifically, MDD - frailty index (N_available = 175,226 vs N_required = 52,600; 3.3×), feeling lonely - MDD (500,199 vs 203,700; 2.5×), MDD - feeling lonely (376,352 vs 41,900; 9.0×), LDL cholesterol - coronary heart disease (184,305 vs 4,200; 44×), and triglycerides → coronary heart disease (184,305 vs 25,100; 7.3×)

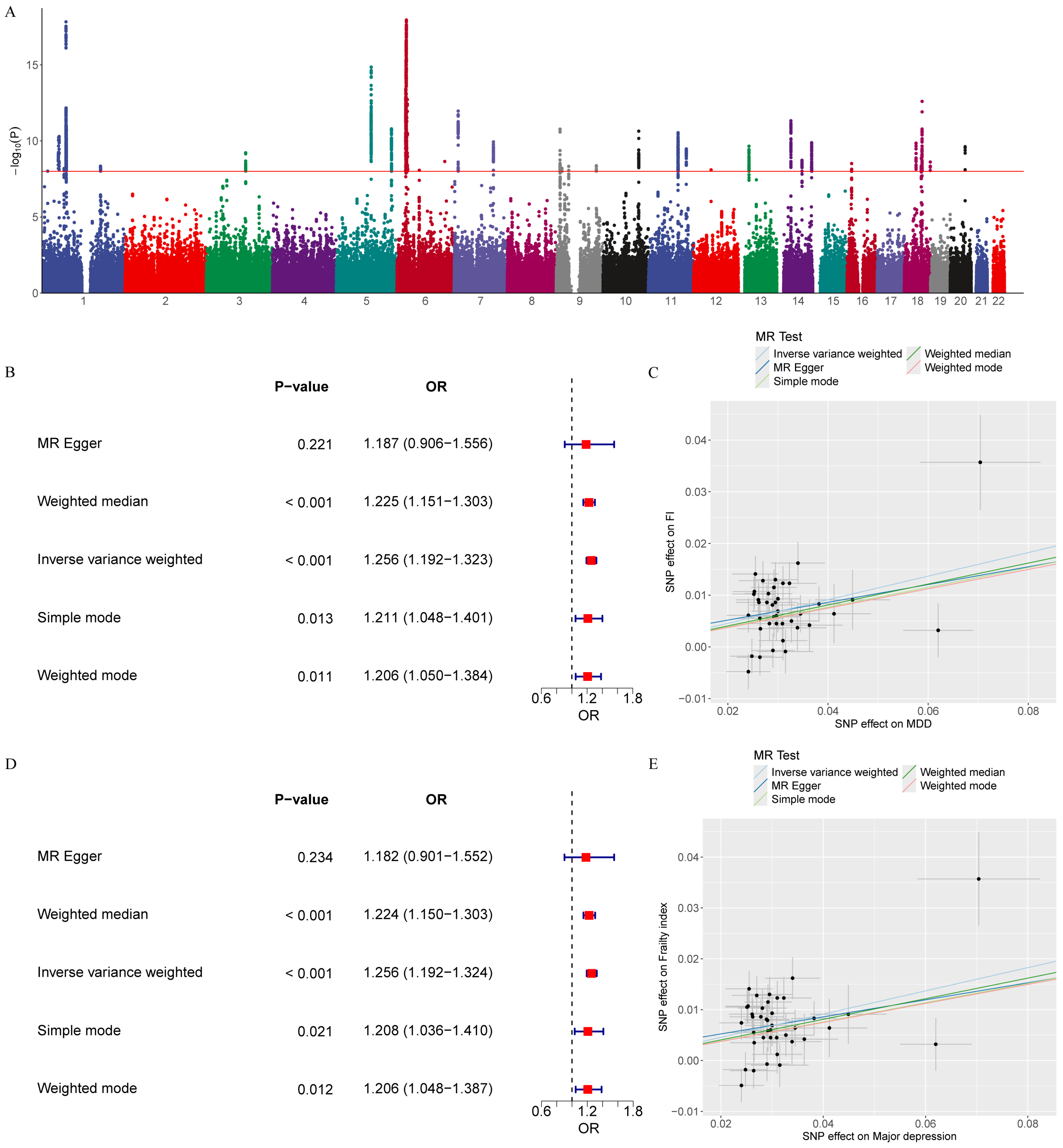

Figure 7. The results of 2SMR analysis. (A) Manhattan plot showing distribution of *P*-values from genome-wide association study of MDD. (B) Forest plot of MR analysis of the MDD and FI using local mode. (C) Individual estimates about the effect of MDD on FI using local mode. The X-axis shows the SNP effect and SE on each of the instrumental variables of MDD. The Y-axis shows the SNP effect and SE on FI. Analyses were conducted by using MR Egger, weighted median, inverse variance weighted, simple mode and weighted mode. The slope of each line corresponds to the estimated MR effect per method. (D) Forest plot of MR analysis of MDD (ieu-b-102) and FI (ebi-a-GCST90020053) using API mode. (E) Individual estimates about the effect of MDD on FI using API mode. 2SMR, two-sample Mendelian randomization; MDD, major depression disorder; FI, frailty index; SNP, single nucleotide polymorphism; API, application programming interface; OR, odd ratio; SE, standard error.

were all adequately powered. In contrast, feeling lonely - cigarettes smoked per day (249,752 vs 3,250,800; 0.08×), cigarettes smoked per day - MDD (500,199 vs 1,241,241,600; 0.0004×), HDL cholesterol - coronary heart disease (184,305 vs 538,500; 0.34×), and creatinine - coronary heart disease (184,305 vs 2,513,800; 0.07×) were underpowered, with the smoking-to-MDD analysis particularly constrained by an effect estimate near the null (log OR $\approx$ 0.0014; OR $\approx$ 1.0014), implying an unrealistically large required sample size, (Table 3).

These comparisons indicate that for adequately powered pairs, the minimum detectable effect size given the available sample size was smaller than the estimated causal effect, whereas for underpowered pairs, it exceeded the observed magnitude. For binary outcomes, calculations accounted for the case–control composition of the corresponding GWAS (case fractions 0.49–0.52, as reported). In two-sample analyses, the outcome sample size was defined as the number of participants contributing to the variant–outcome association estimates, in accordance with the calculator's conventions.

Table 3. Feature comparisons of currently available rs id conversion tools (local and web tools)

Exposure	Outcome	Outcome Type	rsq[a]	b1[b]	OR[c]	ratio[d]	Actual[e]	Estimated[f]
ieu-b-102	ebi-a-GCST90020053	continuous	0.0038	0.1976	1.2185	-	175226	52600
ebi-a-GCST006942	ieu-b-102	binary	0.0036	0.2187	1.2445	0.5183	500199	203700
ieu-b-102	ebi-a-GCST006942	continuous	0.0097	0.1393	1.1494	-	376352	41900
ebi-a-GCST006942	ieu-b-142	continuous	0.0037	0.0255	1.0258	-	249752	3250800
ieu-b-142	ieu-b-102	binary	0.0139	0.0014	1.0014	0.5183	500199	1241241600
ieu-a-299	ieu-a-7	binary	0.0537	0.0350	1.0357	0.4923	184305	538500
ieu-a-300	ieu-a-7	binary	0.0503	0.4110	1.5084	0.4923	184305	4200
ieu-a-302	ieu-a-7	binary	0.0473	0.1731	1.1890	0.4923	184305	25100
met-d-Creatinine	ieu-a-7	binary	0.0358	-0.0199	0.9803	0.4923	184305	2513800

a. coefficient of determination (R^2) of exposure on genetic variants; b.causal effect; c. Odds Ratio; d. ratio of case:controls; e. actual sample size; f. estimated minimum sample size.

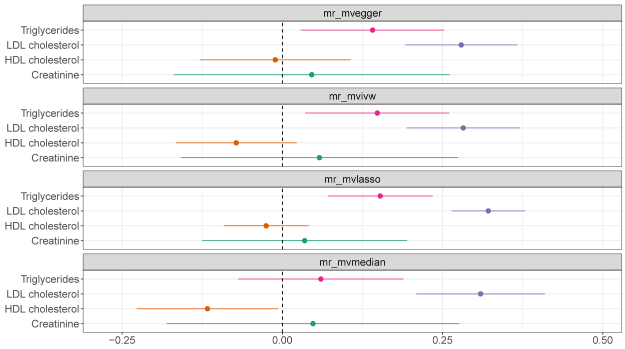

Figure 8. Multivariable Mendelian randomization (MVMR) models investigating the effect of creatinine and major lipid species on coronary heart disease. Each panel represents the results from a different MVMR model (each with different underlying assumptions). MR, Mendelian randomization; LDL cholesterol, low-density lipoprotein cholesterol; HDL cholesterol, high-density lipoprotein cholesterol; IVW, inverse variance weighted.

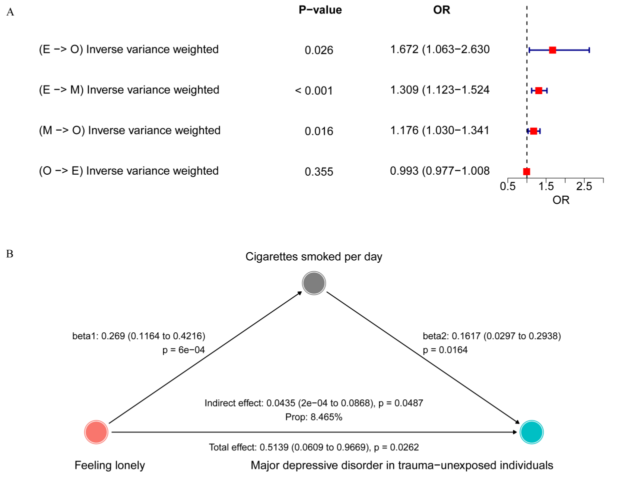

Figure 9. The results of mediation Mendelian randomization (MMR) analysis. (A) Forest plot of four-step two-sample MR approaches; (B) Directed acyclic graph (DAG) for the causal mediation analyses for the complex relationships between feeling lonely, major depressive disorder in trauma-unexposed individuals (MDD), and cigarettes smoked per day. E, exposure; O, outcome; M, mediation; MR, Mendelian randomization; DAG, directed acyclic graph; MDD, major depressive disorder in trauma-unexposed individuals; OR, odd ratio.

Discussion

MRanalysis and GWASkit provide a comprehensive and user-friendly platform for MR analyses and processing GWAS summary data. The development of our platform addresses several key challenges in the field, such as the complexity of different MR methods, the lack of standardized workflows, and the need for extensive coding experience. By integrating data preprocessing, QC, MR analysis and visualization, MRanalysis streamlines the entire MR workflows, making it more accessible to researchers with diverse backgrounds and varying levels of coding expertise (all applications in MRanalysis include test data and complete operation GIFs are recorded).

While MRanalysis democratizes MR methodology for researchers across diverse backgrounds, we acknowledge the critical importance of preventing methodological misuse. Increased accessibility raises legitimate concerns about atheoretical applications—particularly conducting cross-trait analyses without biological justification or examining arbitrarily selected exposure-outcome pairs. MR's validity as a causal inference framework fundamentally depends on satisfying core assumptions and establishing biologically plausible relationships. Users must ground analyses in well-defined, evidence-based hypotheses and adhere to established best-practice guidelines [22]. MRanalysis enforces comprehensive quality control standards including instrument strength evaluation (F-statistics), heterogeneity assessment (Cochran's Q), horizontal pleiotropy detection (MR-Egger intercept, MR-PRESSO), and mandatory power calculations. Violation of fundamental assumptions—relevance, independence, and exclusion restriction—particularly when selecting exposure-outcome pairs without considering biological plausibility or pleiotropic pathways, invariably yields spurious causal inferences. To facilitate methodologically sound research, MRanalysis incorporates automated quality checks, sample size calculators, transparent assumption reporting, and real-time analytical warnings. This integrated framework balances accessibility with methodological rigor, fostering responsible implementation while emphasizing that biological plausibility and methodological scrutiny remain paramount for valid causal inference in genetic epidemiology.

One of the major strengths of MRanalysis is the modular structure, which allows users to perform a wide range of analyses, from univariable and multivariable to mediation MR. Our platform also offers additional utilities, such as power and sample size calculators, SNP to gene annotation, and enrichment analysis, further enhancing the robustness and reliability of MR studies. The incorporation of best practices and guideline proposed by Burgess et al. [22] ensures the standardization of MR analyses and improves the quality and reproducibility of the results. Interpretation of MR results requires comprehensive evidence synthesis rather than reliance on isolated analyses. MRanalysis promotes a robust causal inference framework that integrates multiple analytical strategies, combining univariable MR for establishing basic causal relationships, multivariable MR for controlling confounding exposures, and mediation MR for elucidating causal pathways. Our platform facilitates robust validation through the integration of complementary analyt-

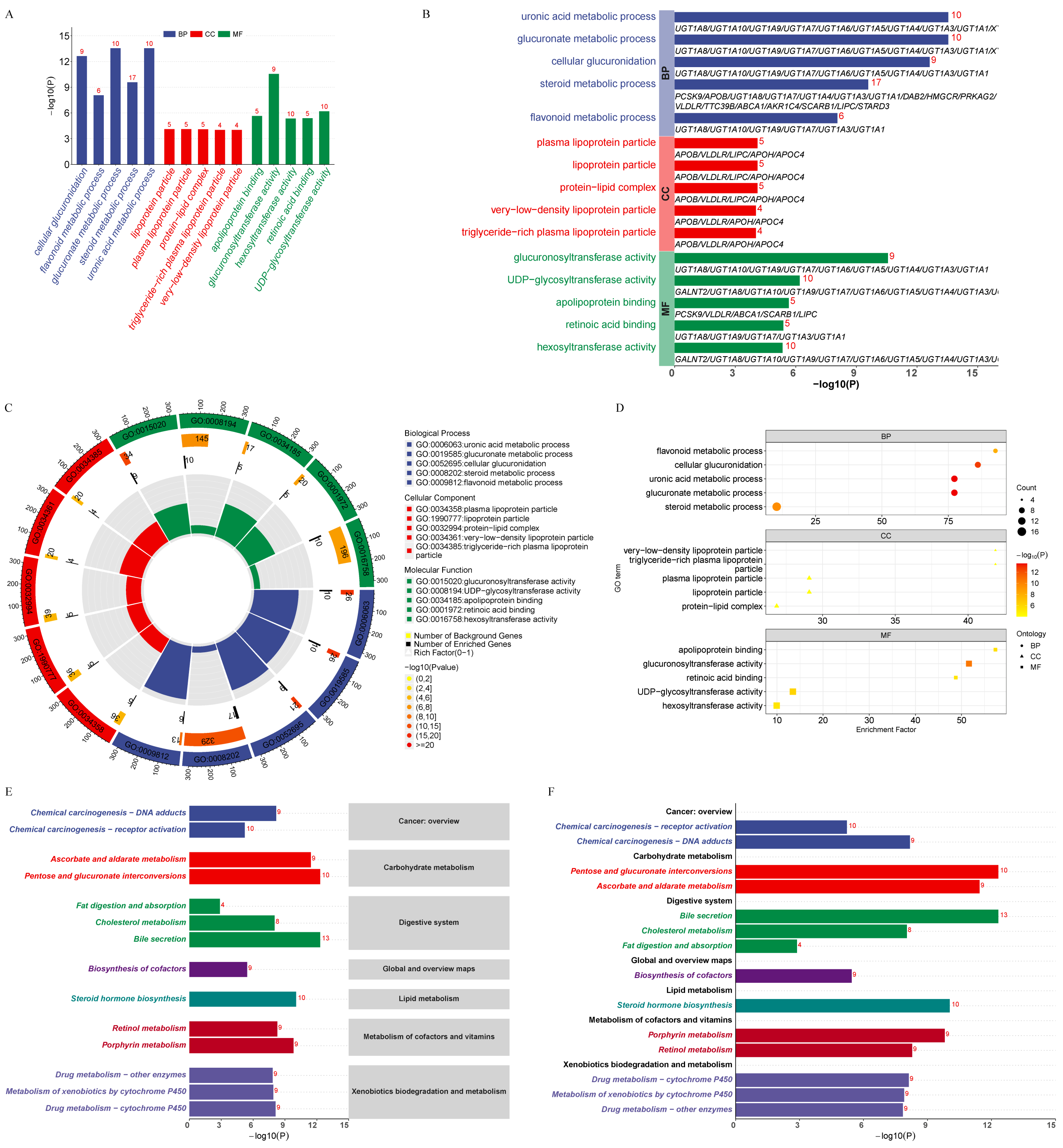

Figure 10. The results of GO/KEGG enrichment analyses. A. a vertical bar chart displaying GO enrichment; B. a horizontal bar chart displaying GO enrichment; C. a circos plot displaying GO enrichment; D. a dot plot displaying GO enrichment; E. a hierarchical horizontal bar chart displaying KEGG enrichment (Style I); F. a hierarchical horizontal bar chart displaying KEGG enrichment (Style II). GO, gene ontology; BP, biological process; CC, cellular component; MF, molecular function.

ical approaches, encompassing rigorous quality control procedures, colocalization analyses, fine-mapping studies, and pathway enrichment analyses where feasible. We emphasize that MR findings should be contextualized within broader evidence bases, including experimental validation, replication across diverse populations, and consistency with observational epidemiological studies. This framework treats MR results as one line of evidence for causality, not definitive proof, helping prevent overinterpretation that could mislead clinical decisions.

GWASkit, as a standalone tool and bridge, complements MRanalysis by facilitating the preprocessing of kinds of GWAS summary datasets. It is efficient handling of tasks such as rs ID mapping, format conversion, and data standardization enables seamless compatibility with a wide range of post-GWAS tools and applications within the MRanalysis platform. These case studies demonstrated the versatility, convenience and robustness of our platform. In particular, the case study on rs ID conversion shows that our GWASkit outperformed other existing tools, exhibiting high accuracy, fast processing speed, and moderate memory usage. In addition, the availability of GWASkit on multiple operating systems and its user-friendly interface makes it accessible to researchers with limited programming skills.

Currently, there are several online platforms for MR analysis (Table 4), with MR-Base [44] being one of the earliest, appearing in 2018. MR-Base is an analytical platform for MR developed by the MRC Integrative Epidemiology Unit (IEU) at the University

of Bristol. This team has also developed the OpenGWAS database [50], which is a database of genetic associations from GWAS summary dataset, available for online querying and download. Since the launch of the MR-Base and OpenGWAS database, other online tools have emerged, each with a slightly different focus. But most of these tools primarily support the two-sample MR approach and do not offer functionality for multivariable and mediation MR. Furthermore, these tools often lack the necessary steps for preprocessing and QC of GWAS datasets. In contrast to the existing platforms, MRanalysis is specially designed for MR analyses and related GWAS data processing. We aim to establish a comprehensive, standardized, and user-friendly open platform that caters to the needs of users conducting MR studies. By providing a complete workflow that includes data preprocessing, QC, and various MR approaches, our platform seeks to address the limitations of current platforms and facilitate more robust and reproducible MR analyses.

Another key strengths of MRanalysis lies in its transparency and reproducibility. By providing full access to the underlying code, we empower researchers to not only understand the intricacies of their analyses but also to modify and extend them as needed. This approach fosters a deeper engagement with the analytical process and promotes methodological rigor in MR studies. By integrating best practices and standardized workflows, we strive to ensure the reliability and consistency of MR analyses conducted using our platform. A crucial feature of MRanalysis is the real-time generation of complete code for all applications based on user-customized parameters. This allows users to gain a deeper understanding of the analytical steps involved. Simultaneously, those with extensive programming experience may prefer more flexible and customizable options. Leveraging this feature, users can generate code from the applications and then perform further personalized analyses locally. This capability bridges the gap between user-friendly interfaces and the need for advanced customization, catering to researchers with varying levels of technical expertise.

However, it's important to acknowledge some limitations and future directions for MR analysis and GWASkit. Several methodological limitations inherent to two-sample Mendelian randomization necessitate careful consideration during result interpretation. The fundamental assumption of shared genetic architecture and linkage disequilibrium patterns between exposure and outcome GWAS cohorts may be compromised when integrating studies across diverse ancestral populations or geographic regions. Differential population stratification between samples can introduce systematic bias, thereby undermining the validity of causal estimates. Furthermore, two-sample MR quantifies lifelong genetic effects, which may not accurately reflect the therapeutic potential of short-term interventions or late-onset exposures of clinical relevance. The methodology remains vulnerable to horizontal pleiotropy, wherein genetic instruments influence outcomes through biological pathways independent of the target exposure. Detecting such pleiotropic effects using summary-level statistics presents considerable analytical challenges. Statistical power is contingent upon instrument strength and GWAS sample dimensions, with weak instrument bias persisting despite apparently adequate F-statistics. Additional methodological concerns include collider bias, and the inability to model time-varying associations or non-linear dose-response relationships, all of which may confound causal inference. Thus, investigators should interpret two-sample MR findings as contributory evidence toward causal inference rather than definitive proof of causation, particularly when considering clinical translation. These methodological constraints underscore the critical importance of our platform's emphasis on comprehensive quality control procedures and multi-method validation for responsible causal inference in biomedical research. As the field of MR continues to evolve, the platform will require regular updates to incorporate new methods and address emerging challenges. Future developments may focus on expanding the platform's compatibility with a broader range of data formats and integrating more advanced visualization tech-

Table 4. Feature comparisons of currently available Mendelian randomization analysis platform.

Platform	Input	Data preprocessing	Free	QC	2SMR	MVMR	MMR	Colocalization Analysis	Visualization	Year	Cite
**MRanalysis**	**BI, UP**	**Yes**	**Yes**	**Yes**	**Yes**	**Yes**	**Yes**	**Yes**	**Yes**	**2025**[a]	-
MR-base	BI, UP[b]	No	Yes	No	Yes	No	No	No	No	2018	Hemani et al. [44]
MetaboAnalyst	BI[c]	No	Yes	No	Yes	No	No	No	Yes	2024	Pang et al. [45]
HiOmics	BI, UP	No	No	No	Yes	No	No	No	Yes	2023	Li et al. [46]
SUMMER	BI[d]	No	Yes	No	Yes	No	No	No	Yes	2019	Xin et al. [47]
MRbrowse	BI[e]	No	Yes	No	Yes	No	No	No	Yes	2018	-
ExPheWas	BI[f]	No	Yes	No	Yes	No	No	No	Yes	2021	Legault et al. [48]
MRAD	BI[g]	No	Yes	No	Yes	No	No	No	No	2024	Zhao et al. [49]

MR: Mendelian randomization; QC, quality control; 2SMR, two-sample MR analysis; MVMR: multivariable MR; MMR: Mediation MR; BI: built-in; UP: upload.
a. Last update time. b. Only the exposure data can be uploaded by users, while the outcome data is not supported for user uploads; c. the exposure data primarily focuses on metabolites; d. the outcome data primarily focuses on cancers; e. preperformed Mendelian Randomization (MR) analyses of 100s of proteins against 100s of clinical outcomes using genetic data; f. The platform reports on genetic associations between genes and phenotypes; g. The MRAD application was created to identify the risk of protective factors for Alzheimer's disease.

niques to facilitate result interpretation.

Conclusion

In summary, MRanalysis and GWASkit offer a comprehensive, efficient and user-centric solution for conducting MR analyses and handling GWAS summary data. By providing a unified and standardized platform that integrates diverse functionalities and promotes best practices, this platform has the potential to accelerate discoveries in genetic epidemiology, ultimately leading to improved understanding of complex diseases and more targeted interventions. As the user base of MRanalysis continues to grow, it is poised to become an essential resource for the genetic epidemiology community, empowering researchers to unravel causal relationships and advance our understanding of human health and disease.

Availability of source code and requirements

- Project name: MRanalysis/GWASkit
- Project home page: https://github.com/xingabao/MRanalysis; https://github.com/Li-OmicsLab-MPU/GWASkit
- Operating system(s): Windows, MacOS, Linux
- Programming language: R, Python, CSS, HTML, Dockerfile
- Other requirements: N/A
- License: The MRanalysis/GWASkit codebase is licensed with a CC0 1.0 license (dataset) and the MIT license.
- Bio.tools id: biotools:mranalysis (https://bio.tools/mranalysis)
- RRID:SCR_027338
- WorkflowHub: 10.48546/WORKFLOWHUB.WORKFLOW.1872.1

Abbreviations

API: application programming interface; BP: biological processes; CC: cellular components; CHD: coronary heart disease; CPU: central processing unit; DAG: Directed Acyclic Graph; FI: frailty index; GO: Gene Ontology; GWAS: genome-wide association study; HDL cholesterol: high-density lipoprotein cholesterol; IV: instrulmental variable; IVW: inverse variance weighted; KEGG: Kyoto Encyclopedia of Genes and Genomes; LD: linkage disequilibrium; LDL cholesterol: low-density lipoprotein cholesterol; LTS: long-term support ; MDD: major depression disorder; MF: molecular functions; MR: Mendelian randomization; MVMR: multivariable Mendelian randomization; OR: odds ratios; PGC: Psychiatric Genomics Consortium; QC: quality control; RCT: randomized controlled trail; SNP: single nucleotide polymorphism; UI: user interface; VCF: variant call format

Data Availability

Publicly available datasets were analyzed in this study. The GWAS summary statistics can be found in the MRC IEU OpenGWAS project, the EMBL-EBI GWAS Catalog, and the Edinburgh DataShare repository. The specific datasets used in this study are available under the following accession IDs: Major depression (MDD): Edinburgh DataShare (doi:10.7488/ds/2458) and OpenGWAS (ieu-b-102), Howard et al. [25]; frailty index: GWAS Catalog (GCST90020053) and OpenGWAS (ebi-a-GCST90020053), Atkins et al. [24]; HDL cholesterol: OpenGWAS (ieu-a-299), Gustafsson et al. [26]; LDL cholesterol: OpenGWAS (ieu-a-300), Gustafsson et al. [26]; Triglycerides: OpenGWAS (ieu-a-302), Gustafsson et al. [26]; Creatinine: OpenGWAS (met-d-Creatinine); Coronary heart disease: OpenGWAS (ieu-a-7), Nikpay et al. [27]; Feeling lonely: OpenGWAS (ebi-a-GCST006942), Nagel et al. [28]; Major depressive disorder in trauma-unexposed individuals (MDD): OpenGWAS (ebi-a-GCST009981), Coleman et al. [29]; Cigarettes smoked per day: OpenGWAS (ieu-b-142), Liu et al. [30]. The database search was completed on August 15, 2024.

Acknowledgements

We gratefully acknowledge the invaluable contributions of all data generators and consortiums whose publicly available GWAS summary statistics made this work possible, including but not limited to the Psychiatric Genomics Consortium, UK Biobank, EMBL-EBI GWAS Catalog, and the MRC IEU OpenGWAS project. We also thank the developers and maintainers of key open-source resources and analytical tools utilized in this study, including dbSNP (NCBI), TwoSampleMR, MVMR, MAGMA, CheckSumStats, clusterProfiler, and other essential R and Python packages.

We sincerely appreciate the broader R, Python, and bioinformatics communities, whose continued efforts have provided the foundations for the development of MRanalysis and GWASkit.

Author Contributions

Abao Xing: Investigation, Conceptualization, Formal analysis, Methodology, Validation, Writing—original draft & editing. Tiantian Cai: Investigation, Data curation, Formal analysis, Visualization, Writing—review & editing. Haofan Du Investigation, Formal analysis, Visualization, Writing—review & editing. Zhifan Li: Methodology, Visualization. HoiMan Ng: Supervision, Validation. Junrong Li: Methodology, Formal analysis. Guanmin Jiang: Supervision, Validation. Lijun Chen: Conceptualization, Supervision, Methodology, Validation. Kefeng Li: Supervision, Project administration, Funding acquisition, Writing – review & editing.

Funding

This work was supported by the fund from Macao Polytechnic University (RP/FCA-14/2023), The Science and Technology Development Funds(FDCT) of Macao (0033/2023/RIB2), and the Joint Research Funding Program between FDCT and the Department of Science and Technology of Guangdong Province (FDCT-GDST) (0009/2024/AGJ) with the submission approval IDfca.f852.focf,2].

Competing Interests

The authors declare that they have no competing interests.

Ethics Statement

All test data in this study were derived from public GWAS summary-level data, ethics approval was not required for the present study.

References

1. Hu X, Zhao J, Lin Z, Wang Y, Peng H, Zhao H, et al. Mendelian randomization for causal inference accounting for pleiotropy and sample structure using genome-wide summary statistics. Proceedings of the National Academy of Sciences 2022;119(28):e2106858119. https://doi.org/10.1073/pnas.2106858119.
2. Boehm FJ, Zhou X. Statistical methods for Mendelian randomization in genome-wide association studies: a review. Computational and structural biotechnology journal 2022;20:2338–2351. https://doi.org/10.1016/j.csbj.2022.05.015.
3. Ference BA, Holmes MV, Smith GD. Using Mendelian randomization to improve the design of randomized trials. Cold

Spring Harbor perspectives in medicine 2021;11(7):a040980. https://doi.org/10.1101/cshperspect.a040980.
4. Hernán MA, Wang W, Leaf DE. Target trial emulation: a framework for causal inference from observational data. JAMA 2022;328(24):2446–2447. https://doi.org/10.1001/jama.2022.21383.
5. Broglio K. Randomization in clinical trials: permuted blocks and stratification. JAMA 2018;319(21):2223–2224. https://doi.org/10.1001/jama.2018.6360.
6. Yu H, Wan X, Yang M, Xie J, Xu K, Wang J, et al. A large-scale causal analysis of gut microbiota and delirium: a Mendelian randomization study. Journal of affective disorders 2023;329:64–71. https://doi.org/10.1016/j.jad.2023.02.078.
7. Li J, Tang M, Gao X, Tian S, Liu W. Mendelian randomization analyses explore the relationship between cathepsins and lung cancer. Communications biology 2023;6(1):1019. https://doi.org/10.1038/s42003-023-05408-7.
8. Ye CJ, Liu D, Chen ML, Kong LJ, Dou C, Wang YY, et al. Mendelian randomization evidence for the causal effect of mental well-being on healthy aging. Nature Human Behaviour 2024;8(9):1798–1809. https://doi.org/10.1038/s41562-024-01905-9.
9. Pozarickij A, Gan W, Lin K, Clarke R, Fairhurst-Hunter Z, Koido M, et al. Causal relevance of different blood pressure traits on risk of cardiovascular diseases: GWAS and Mendelian randomisation in 100,000 Chinese adults. Nature Communications 2024;15(1):6265. https://doi.org/10.1038/s41467-024-50297-x.
10. Cronjé HT, Karhunen V, Hovingh GK, Coppieters K, Lagerstedt JO, Nyberg M, et al. Genetic evidence implicating natriuretic peptide receptor-3 in cardiovascular disease risk: a Mendelian randomization study. BMC medicine 2023;21(1):158. https://doi.org/10.1186/s12916-023-02867-x.
11. Larsson SC, Butterworth AS, Burgess S. Mendelian randomization for cardiovascular diseases: principles and applications. European heart journal 2023;44(47):4913–4924. https://doi.org/10.1093/eurheartj/ehad736.
12. Levin MG, Burgess S. Mendelian randomization as a tool for cardiovascular research: a review. JAMA cardiology 2024;9(1):79–89. https://doi.org/10.1001/jamacardio.2023.4115.
13. Wang Z, Chen J, Zhu L, Jiao S, Chen Y, Sun Y. Metabolic disorders and risk of cardiovascular diseases: a two-sample mendelian randomization study. BMC Cardiovascular Disorders 2023;23(1):529. https://doi.org/10.1186/s12872-023-03567-3.
14. Du Zx, Ren Yy, Wang Jl, Li Sx, Hu Yf, Wang L, et al. The potential association between metabolic disorders and pulmonary tuberculosis: a Mendelian randomization study. European Journal of Medical Research 2024;29(1):277. https://doi.org/10.1186/s40001-024-01845-0.
15. Mu C, Dang X, Luo XJ. Mendelian randomization analyses reveal causal relationships between brain functional networks and risk of psychiatric disorders. Nature human behaviour 2024;8(7):1417–1428. https://doi.org/10.1038/s41562-024-01879-8.
16. Gao X, Qin Y, Jiao S, Hao J, Zhao J, Wang J, et al. Genetic evidence for the causal relations between metabolic syndrome and psychiatric disorders: a Mendelian randomization study. Translational Psychiatry 2024;14(1):46. https://doi.org/10.1038/s41398-024-02759-5.
17. Yu Y, Hou L, Wu Y, Yu Y, Liu X, Wu S, et al. Causal associations between female reproductive behaviors and psychiatric disorders: a lifecourse Mendelian randomization study. BMC psychiatry 2023;23(1):799. https://doi.org/10.1186/s12888-023-05203-y.
18. Wang W, Li W, Zhang D, Mi Y, Zhang J, He G. The causal relationship between PCSK9 inhibitors and malignant tumors: a mendelian randomization study based on drug targeting. Genes 2024;15(1):132. https://doi.org/10.3390/genes15010132.
19. Burgess S, Mason AM, Grant AJ, Slob EA, Gkatzionis A, Zuber V, et al. Using genetic association data to guide drug discovery and development: review of methods and applications. The American Journal of Human Genetics 2023;110(2):195–214. https://doi.org/10.1016/j.ajhg.2022.12.017.
20. Duan QQ, Wang H, Su WM, Gu XJ, Shen XF, Jiang Z, et al. TBK1, a prioritized drug repurposing target for amyotrophic lateral sclerosis: evidence from druggable genome Mendelian randomization and pharmacological verification in vitro. BMC medicine 2024;22(1):96. https://doi.org/10.1186/s12916-024-03314-1.
21. Wootton RE, Sallis HM. Let's call it the effect allele: a suggestion for GWAS naming conventions. International journal of epidemiology 2020;49(5):1734–1735. https://doi.org/10.1093/ije/dyaa149.
22. Burgess S, Smith GD, Davies NM, Dudbridge F, Gill D, Glymour MM, et al. Guidelines for performing Mendelian randomization investigations: update for summer 2023. Wellcome open research 2023;4:186. https://doi.org/10.12688/wellcomeopenres.15555.3.
23. Wray NR, Ripke S, Mattheisen M, Trzaskowski M, Byrne EM, Abdellaoui A, et al. Genome-wide association analyses identify 44 risk variants and refine the genetic architecture of major depression. Nature genetics 2018;50(5):668–681. https://doi.org/10.1038/s41588-018-0090-3.
24. Atkins JL, Jylhävä J, Pedersen NL, Magnusson PK, Lu Y, Wang Y, et al. A genome-wide association study of the frailty index highlights brain pathways in ageing. Aging cell 2021;20(9):e13459. https://doi.org/10.1111/acel.13459.
25. Howard DM, Adams MJ, Clarke TK, Hafferty JD, Gibson J, Shirali M, et al. Genome-wide meta-analysis of depression identifies 102 independent variants and highlights the importance of the prefrontal brain regions. Nature neuroscience 2019;22(3):343–352. https://doi.org/10.1038/s41593-018-0326-7.
26. Gustafsson SK, Ganna A, et al. Discovery and refinement of loci associated with lipid levels. Nature Genetics 2013;45(11):1274–1283. https://doi.org/10.1038/ng.2797.
27. Nikpay M, Goel A, Won H, Hall L, Willenborg C, Kanoni S, et al. A comprehensive 1000 Genomes–based genome-wide association meta-analysis of coronary artery disease. Nature genetics 2015;47(10):1121–1130. https://doi.org/10.1038/ng.3396.
28. Nagel M, Watanabe K, Stringer S, Posthuma D, Van Der Sluis S. Item-level analyses reveal genetic heterogeneity in neuroticism. Nature communications 2018;9(1):905. https://doi.org/10.1038/s41467-018-03242-8.
29. Coleman JR, Peyrot WJ, Purves KL, Davis KA, Rayner C, Choi SW, et al. Genome-wide gene-environment analyses of major depressive disorder and reported lifetime traumatic experiences in UK Biobank. Molecular psychiatry 2020;25(7):1430–1446. https://doi.org/10.1038/s41380-019-0546-6.
30. Liu M, Jiang Y, Wedow R, Li Y, Brazel DM, Chen F, et al. Association studies of up to 1.2 million individuals yield new insights into the genetic etiology of tobacco and alcohol use. Nature genetics 2019;51(2):237–244. https://doi.org/10.1038/s41588-018-0307-5.
31. Surapaneni A, Schlosser P, Zhou L, Liu C, Chatterjee N, Arking DE, et al. Identification of 969 protein quantitative trait loci in an African American population with kidney disease attributed to hypertension. Kidney international 2022;102(5):1167–1177. https://doi.org/10.1016/j.kint.2022.07.005.
32. Haycock PC, Borges MC, Burrows K, Lemaitre RN, Harrison S, Burgess S, et al. Design and quality control of large-scale two-sample Mendelian randomization studies. International journal of epidemiology 2023;52(5):1498–1521. https://doi.org/10.1093/ije/dyad018.

33. Wang Z, Dou Y, Chen L, Feng W, Zou Y, Xiao J, et al. Mendelian randomization identifies causal effects of major depressive disorder on accelerated aging. Journal of Affective Disorders 2024;358:422–431. https://doi.org/10.1016/j.jad.2024.05.056.
34. De Leeuw CA, Mooij JM, Heskes T, Posthuma D. MAGMA: generalized gene-set analysis of GWAS data. PLoS computational biology 2015;11(4):e1004219. https://doi.org/10.1371/journal.pcbi.1004219.
35. Xu S, Hu E, Cai Y, Xie Z, Luo X, Zhan L, et al. Using clusterProfiler to characterize multiomics data. Nature protocols 2024;19(11):3292–3320. https://doi.org/10.1038/s41596-024-01020-z.
36. Burgess S. Sample size and power calculations in Mendelian randomization with a single instrumental variable and a binary outcome. International journal of epidemiology 2014;43(3):922–929. https://doi.org/10.1093/ije/dyu005.
37. Brion MJA, Shakhbazov K, Visscher PM. Calculating statistical power in Mendelian randomization studies. International journal of epidemiology 2013;42(5):1497–1501. https://doi.org/10.1093/ije/dyt179.
38. Davies NM, Holmes MV, Smith GD. Reading Mendelian randomisation studies: a guide, glossary, and checklist for clinicians. bmj 2018;362. https://doi.org/10.1136/bmj.k601.
39. Wang K, Li M, Hakonarson H. ANNOVAR: functional annotation of genetic variants from high-throughput sequencing data. Nucleic acids research 2010;38(16):e164–e164. https://doi.org/10.1093/nar/gkq603.
40. Cingolani P, Platts A, Wang LL, Coon M, Nguyen T, Wang L, et al. A program for annotating and predicting the effects of single nucleotide polymorphisms, SnpEff: SNPs in the genome of Drosophila melanogaster strain w1118; iso-2; iso-3. fly 2012;6(2):80–92. https://doi.org/10.4161/fly.19695.
41. Murphy AE, Schilder BM, Skene NG. MungeSumstats: a Bioconductor package for the standardization and quality control of many GWAS summary statistics. Bioinformatics 2021;37(23):4593–4596. https://doi.org/10.1093/bioinformatics/btab665.
42. He Y, Koido M, Shimmori Y, Kamatani Y. GWASLab: a Python package for processing and visualizing GWAS summary statistics. Jxiv 2023;https://doi.org/10.51094/jxiv.370.
43. Oscanoa J, Sivapalan L, Gadaleta E, Dayem Ullah AZ, Lemoine NR, Chelala C. SNPnexus: a web server for functional annotation of human genome sequence variation (2020 update). Nucleic acids research 2020;48(W1):W185–W192. https://doi.org/10.1093/nar/gkaa420.
44. Hemani G, Zheng J, Elsworth B, Wade KH, Haberland V, Baird D, et al. The MR-Base platform supports systematic causal inference across the human phenome. elife 2018;7:e34408. https://doi.org/10.7554/eLife.34408.
45. Pang Z, Lu Y, Zhou G, Hui F, Xu L, Viau C, et al. MetaboAnalyst 6.0: towards a unified platform for metabolomics data processing, analysis and interpretation. Nucleic acids research 2024;52(W1):W398–W406. https://doi.org/10.1093/nar/gkae253.
46. Li W, Zhang Z, Xie B, He Y, He K, Qiu H, et al. HiOmics: A cloud-based one-stop platform for the comprehensive analysis of large-scale omics data. Computational and Structural Biotechnology Journal 2024;23:659–668. https://doi.org/10.1016/j.csbj.2024.01.002.
47. Xin J, Gu D, Chen S, Ben S, Li H, Zhang Z, et al. SUMMER: a Mendelian randomization interactive server to systematically evaluate the causal effects of risk factors and circulating biomarkers on pan-cancer survival. Nucleic acids research 2023;51(D1):D1160–D1167. https://doi.org/10.1093/nar/gkac677.
48. Legault MA, Perreault LPL, Dubé MP. ExPheWas: a browser for gene-based pheWAS associations. Nucleic acids research 2022;50(W1):W305––W31. https://doi.org/10.1093/nar/gkac289.
49. Zhao T, Li H, Zhang M, Xu Y, Zhang M, Chen L. Systematic evaluation of multifactorial causal associations for Alzheimer's disease and an interactive platform MRAD developed based on Mendelian randomization analysis. Elife 2024;13:RP96224. https://doi.org/10.7554/eLife.96224.
50. Elsworth B, Lyon M, Alexander T, Liu Y, Matthews P, Hallett J, et al. The MRC IEU OpenGWAS data infrastructure. BioRxiv 2020;p. 2020–08. https://doi.org/10.1101/2020.08.10.244293.

Manuscript

Click here to access/download;Manuscript;MRanalysis_GigaScience.pdf

Placeholder for OUP logo oup.pdf

GigaScience, 2023, 1–17

doi: xx.xxxx/xxxx
Manuscript in Preparation
Paper

PAPER

MRanalysis: A Comprehensive Online Platform for Integrated, Multi-Method Mendelian Randomization and Associated Post-GWAS Analyses

Abao Xing[1,†], Tiantian Cai[2,†], Haofan Du[3,†], Zhifan Li[2], Hoiman Ng[4], Junrong Li[1], Guanmin Jiang[5], Lijun Chen[6,*] and Kefeng Li[1,*]

[1]Centre for Artificial Intelligence Driven Drug Discovery, Faculty of Applied Sciences, Macao Polytechnic University, Rua de Luís Gonzaga Gomes, Macao, 999078, Macao SR and [2]Big Data and Internet of Things Program, Faculty of Applied Sciences, Macao Polytechnic University, Rua de Luís Gonzaga Gomes, Macao, 999078, Macao SR and [3]School of Physics and Technology, Nanjing Normal University, Nanjing, Jiangsu, 210023, China and [4]Clinical laboratory, Kiang Wu Hospital, 999078, Macao SR and [5]Department of Clinical Laboratory, The Fifth Affiliated Hospital, Sun Yat-sen University, Zhuhai, Guangdong, 519000, China and [6]Department of Hematology and Rheumatology, Zhuhai People's Hospital (Zhuhai Hospital affiliated with Jinan University), Kangning Rord 97, Zhuhai, 519000, China

*kefengl@mpu.edu.mo; cljshizairenwei@163.com
†Contributed equally.

Abstract

Background: Mendelian randomization (MR) is a powerful epidemiological method for inferring causal relationships between exposures and outcomes using genome-wide association study (GWAS) data. However, its adoption is limited by inconsistent data formats, lack of standardized workflows, and the need for programming expertise. To address these challenges, we developed MRanalysis, a user-friendly, web-based platform for integrated MR analysis, and GWASkit, a standalone tool for GWAS data preprocessing.
Results: MRanalysis provides a comprehensive, no-code workflow for MR analysis, including data quality assessment, power estimation, SNP-to-gene enrichment, and visualization. It supports univariable, multivariable, and mediation MR analyses through an intuitive interface. GWASkit facilitates rapid GWAS data preprocessing, such as rs ID conversion and format standardization, with significantly higher accuracy and efficiency than existing tools. Case studies demonstrate the utility and efficiency of both tools in real-world scenarios.
Conclusions: MRanalysis and GWASkit lower barriers to MR analysis, making it more accessible, reliable, and efficient. By democratizing MR, these tools can accelerate discoveries in genetic epidemiology, inform public health strategies, and guide targeted interventions. MRanalysis is freely available at `https://mranalysis.cn`, and GWASkit can be accessed at `https://github.com/Li-OmicsLab-MPU/GWASkit`. Together, they represent a significant advance in understanding the complex relationships between genes, exposures, and health outcomes.

Key words: Mendelian randomization; GWAS; Online platform; MRanalysis; GWASkit; rs ID conversion; SNP-to-gene enrichement; Visualization

Compiled on: October 12, 2025.
Draft manuscript prepared by the author.

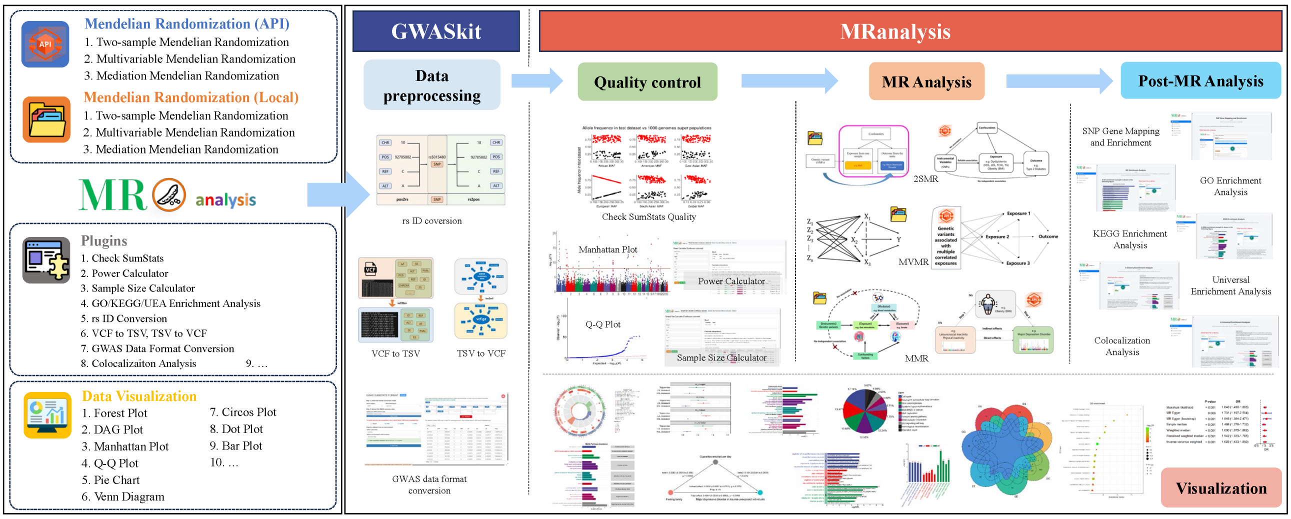

Key Points

- GWASkit Tool: A standalone, installation-free tool for rapid GWAS dataset preprocessing and format standardization, outperforming current existing tools (Such as ANNOVAR, and gwaslab) in both SNP rs ID conversion time and conversion accuracy.
- Versatility: Support various MR methodologies, including univariable, multivariable, and mediation MR analyses, catering to diverse research needs.
- Efficiency and Accuracy: Case studies demonstrate the utility, efficiency, and ease of use of both MRanalysis and GWASkit in real-world scenarios, highlighting their potential to accelerate MR research.
- Real-Time Code Generation: Generates and assembles code based on user-defined parameters, enhancing transparency and reproducibility.
- Visual Guidance: Detailed GIF tutorials for all applications, improving user experience.

Introduction

Mendelian randomization (MR) is a powerful research approach that uses genetic variants (usually SNP, single nucleotide polymorphism) as instrumental variables (IVs) to infer causal relationships between exposures and outcomes [1]. It is based on the stability of genes and Mendel's first and second laws of inheritance [2]. The way genes are allocated determines that the relationship between genes and outcomes is not affected by postnatal environmental, behavioral, socioeconomic, and other confounding factors. Therefore, the causal relationships derived from MR studies are more reasonable and reliable. MR has evolved significantly since its inception. Early MR studies were generally limited by small sample sizes and involved few IVs, resulting in relatively low statistical power. However, with the exponential growth in the number of genome-wide association studies (GWAS) conducted globally, the summary data of tens of millions of relationships between exposures, diseases, and genetic variants have been successively released, continuously increasing the power of MR studies and significantly improving their accuracy.

Compared with traditional observational studies and randomized controlled trials (RCTs), MR studies have more advantages [3]. Observational studies are generally used to assess the causal relationship between exposures and outcomes [4], and RCTs also provide high-level evidence for causal relationship testing [5]. However, due to the need for strict quality control, comprehensive design, long-term follow-up, multi-effect interventions, ethical issues, and compliance, observational studies or RCTs to elucidate disease outcomes are often not feasible. Observational studies or RCTs have difficulties in controlling all potential confounding factors, while MR studies can more effectively avoid these factors. Moreover, by utilizing the inherent characteristics of genetic variants, MR studies can address the common problem of reverse causality in observational studies and provide more reliable causal inference. For instance, MR suggests there is a causal relationship between gut microbiota and delirium [6]; Nahid et al. used MR method to study the association between cathepsins and lung cancer and indicate that elevated cathepsin H levels increase the overall risk of lung cancer, adenocarcinoma, and lung cancer among smokers [7]; Ye et al. performed a two-sample MR analysis to estimate the causal effect of mental well-being, and some mediators were identified [8]. In summary, MR has been instrumental in validating or refuting hypothesized causal relationships in various fields, including cardiovascular diseases [9, 10, 11, 12], metabolic disorders [13, 14], psychiatric conditions [15, 16, 17] and drug discovery [18, 19, 20].

MR has emerged as a powerful approach for investigating causal relationships between exposures and outcomes using GWAS summary data. However, conducting MR analyses can be challenging due to the inconsistency of GWAS data format, the complexity of different methods, the lack of standardized workflows, and the need for extensive coding experience to complete the entire process, which can lead to unreliable results. Meanwhile, the lack of standardization of workflows not only affects the reliability of the results, but also hinders the reproducibility of MR studies across different research groups. Existing tools for handling or visualizing GWAS datasets and conducting MR analysis are mostly implemented in R software and focus on specific functionalities, such as specific MR approaches, data munging, or plotting. The fragmentation of workflows further complicates the MR analysis process, as researchers must navigate multiple packages or tools and integrate them into a coherent workflow. These tools also often lack user-friendly interfaces, making them inaccessible to researchers without extensive programming skills. Furthermore, data preprocessing, a crucial and fundamental step in MR analysis, remains a significant hurdle, hindering the widespread adoption of MR in genetic epidemiology investigations. The lack of standardization in

GWAS summary data formats across different databases or consortiums complicates the usage of these data. Despite efforts to develop a standard GWAS format, the large number of existing unprocessed GWAS summary data remains a challenge for data sharing and efficient reuse. It is therefore vital that we can ensure consistency across these datasets to minimize the risk of analytical mistakes due to user error. One such inconsistency is the naming of the effect allele and non-effect allele in these datasets [21]. Besides, the missingness of certain information (such as rs ID) and certain value conversions like -log10 transformation of *P*-values can also hinder the direct reuse of these GWAS data, especially for beginners, and are error-prone during data and format conversions without careful reading manuals. Moreover, the conversion of SNP identifiers, specifically from CHR:POS:REF:ALT (chromosome, base pair location, non-effect allele, and effect allele) to rs IDs, is often time-consuming and inefficient with existing tools, and the need for strong programming skills to use command-line tools, coupled with the complex installation and deployment processes, further impedes the broader adoption of MR in genetic epidemiology studies.

To address these challenges in MR analyses and make them more accessible, reliable, and reproducible, we developed MRanalysis, an integrated, versatile and comprehensive web-based platform for MR as well as some post-GWAS/MR analyses, and GWASkit, a standalone tool for rapid GWAS data preprocessing, ensures seamless compatibility with a wide range of other post-GWAS tools and applications in our platform through functions such as rs ID conversion, data format standardization, data extraction. Our aim was to create an intuitive, efficient, and user-friendly toolset that streamlines the entire MR workflows while enhancing accessibility for researchers across disciplines. By incorporating the guidelines proposed by Burgess et al. [22], we standardized MR analyses and ensured adherence to best practices, improving the quality and reproducibility of MR studies. The combination of these tools provides a comprehensive solution for handling and visualizing GWAS data, performing various MR analyses, and lowering the barrier to perform MR analyses. By streamlining the MR workflows, promoting best practices, and supporting various MR methodologies, including univariable, multivariable, and mediation MR analyses, our platform has the potential to accelerate MR studies and contribute to a better understanding of causal relationships in complex biological systems.

MRanalysis and GWASkit, as a zero-code platform and tool, meet the needs of kinds of researchers with limited programming experience, lowering the barrier to performing MR analyses. Through the powerful interactive capabilities of the MRanalysis platform, users can perform personalized analyses and visualizations. The platform also provides code generation functionality, assembling code based on the current user-set parameters. This offers further customization and result reproduction for users with some coding ability, allowing them to understand all the details of the entire analysis. Simultaneously, the powerful functionalities of GWASkit facilitate a wide range of post-GWAS analyses, greatly enhancing genetic research using GWAS summary data. To demonstrate the utility, practicality, convenience and reproducibility of our platform and tool, we performed several real-world case studies. The development of MRanalysis and GWASkit represents a significant stride forward in genetic epidemiology research, facilitating more comprehensive investigations into relationships between genes and various phenotypes and accelerating discoveries in genetic epidemiology and drug discovery, ultimately leading to improved public health strategies and more targeted clinical interventions. Such a platform would serve as a bridge linking unprocessed GWAS summary data to post-GWAS/MR analysis tools seamlessly, enabling researchers with diverse backgrounds and varying levels of coding expertise to conduct MR studies with confidence. The increased accessibility and standardization of MR methods will not only enhance the reliability and reproducibility of MR studies but also foster collaboration and knowledge exchange among researchers from different disciplines.

Materials and Methods

Data sources

GWAS summary data regarding the data sources and sample sizes used in this study is outlined in Table 1. All cases utilized publicly available summary-level GWAS data from participants of European descent and no specific ethical approval was necessary for conducting this study. The GWAS summary data for major depression disorder (MDD) is a genome-wide summary statistics from a meta-analysis of 33 cohorts of Psychiatric Genomics Consortium (PGC) (excluding UK BioBank and 23andMe data) was described in Wray et al. [23]. The total number of individuals in this data is 500,199 (329,443 controls and 170,756 cases) with 8,483,301 variants analyzed. The frailty index (FI), derived from the cumulative defect model, served as a metric for assessing frailty severity [24] (sample size is 175,226). Each individual's FI was calculated by dividing the number of defects by the total 49 defects. Individuals received a score of 0 or 1 based on the presence of defects (0 indicating none. A higher FI value indicated greater frailty. Other GWAS data were obtained from IEU OpenGWAS directly. For more detailed information, please refer to Table 1.

rs ID mapping

We evaluated the performance of five locally installed tools, namely GWASkit v1.0.0, ANNOVAR v2020-06-07 (latest version), snpEff v5.2c, MungeSumstats v1.10.1, and gwaslab v3.4.48, along with two web-based tools, the NCBI dbSNP database and SNPnexus, for their ability to handle rs ID conversion tasks. To ensure a comprehensive and concrete assessment, we utilized a large-scale GWAS data (GCST90236305 [31]) downloaded from the EMBL-EBI GWAS Catalog, which contained 14,519,897 variants with complete information, including chromosome, base pair location, other allele (non-effect allele), effect allele, and rs ID, making it an ideal test data. During the evaluation process, we maintained the default parameters and employed the latest default reference data for each of the five local tools to ensure a fair comparison.

The primary testing environment is in a Linux® Ubuntu 20.04.4 LTS operating system, equipped with a 16-core Intel® (CPU), 128GB of RAM, and a 12TB hard drive. Besides, to assess the cross-platform compatibility and the performance of our GWASkit, we additionally tested it on a Windows operating system featuring 16GB of memory, and Intel® 4-core i7-8650U (CPU) with a 1TB hard drive.

GWAS summary data standardization

The GWAS summary data for major depression disorder (MDD) and frailty index (FI) were downloaded from DataShare and GWAS Catalog (above data sources session, Table 1), respectively. However, these two data were not in the standard VCF format, and the MDD GWAS data only contained rs ID without essential chromosome and position information. To address this issue, we utilized the GWASkit rs2pos (rs2pos -I PGC_UKB_depression_genome-wide.txt -O PGC_UKB_depression.tsv.gz –rs MarkerName –rsdb /rsdb/GRCh37 –SEP 1 –rm -V -Z) command to annotate the rs IDs with chromosome number, base-pair position, non-effect allele and effect allele information. Subsequently, we performed the tsv2vcf command (GWASkit tsv2vcf -I PGC_UKB_depression.tsv.gz -O PGC_UKB_depression.vcf.gz –TYPE GRCh37 –CHR CHR –POS POS –REF A2 –ALT A1 –RSID SNP –BETA LogOR –EAF Freq –SE StdErrLogOR –PVALUE P –SS 500199 -V) to standardize

Table 1. GWAS data sources included in the case study

Phenotype	ID[a]	Sample size (overall or case/control)	Consortium or Author	PMID
MDD[a]	ieu-b-102	170,756/329,443	PGC, UK Biobank	30718901, Howard et al. [25]
frailty index	ebi-a-GCST90020053	175,226	UK Biobank, TwinGene	34431594, Atkins et al. [24]
HDL cholesterol	ieu-a-299	187,167	GLGC	24097068, Gustafsson et al. [26]
LDL cholesterol	ieu-a-300	173,082	GLGC	24097068, Gustafsson et al. [26]
Triglyceride	ieu-a-302	177,861	GLGC	24097068, Gustafsson et al. [26]
Creatinine	met-d-Creatinine	110,058	Borges CM	-
coronary heart disease	ieu-a-7	60,801/123,504	CARDIoGRAMplusC4D	26343387, Nikpay et al. [27]
feeling lonely	ebi-a-GCST006942	376,352	Mats N	29500382, Nagel et al. [28]
MDD[c]	ebi-a-GCST009981	9,487/39,677	Jonathan R I C	31969693, Coleman et al. [29]
cigarettes smoked per day	ieu-b-142	249,752	GSCAN	30643251, Liu et al. [30]

PMID: PubMed ID; PGC: Psychiatric Genomics Consortium; HDL: high-density lipoprotein; LDL: low-density lipoprotein.
a. ID in IEU OpenGWAS; b. major depression disorder; c. major depressive disorder in trauma-unexposed individuals.

the GWAS statistics and generate VCF file in standard format, which can be directly uploaded to the MRanalysis online platform for performing MR analysis. In the case of the FI GWAS data, since it already contained all the necessary information for MR analysis, we only need to directly apply the tsv2vcf command for data standardization (GWASkit tsv2vcf -I 34431594-GCST90020053-EFO_0009885.h.tsv.gz –CHR chromosome –POS base_pair_location –REF other_allele –ALT effect_allele –RSID variant_id –BETA variant_id –EAF effect_allele_frequency –SE standard_error –PVALUE p_value –TYPE GRCh37 –SS 175226 -O GCST90020053.vcf.gz -V).

It is worth noting that all the above-mentioned annotation and standardization operations can also be performed using the Windows version of GWASkit. The detailed process for using GWASkit on Windows can be found in the GWASkit help documentation, which is available on GitHub. By leveraging the powerful features of GWASkit, researchers can convert different kinds of formats of GWAS summary statistics into a standard one, enabling seamless integration with the MRanalysis platform for conducting MR analysis. This streamlined workflow not only saves time and effort but also ensures the accuracy and reliability of the results obtained from the MR analysis.

GWAS summary data quality control

MRanalysis is also a convenient platform for processing quality control (QC) of GWAS statistics, integrating three main functionalities: CheckSumStats, Quantile-Quantile (Q-Q) plot, and Manhattan plot.

CheckSumStats is an R package developed by Haycock et al. [32] that provides a quality control pipeline to identify potential metadata errors, summary data issues, and other analytical problems in GWAS results. These errors and issues can introduce substantial bias into downstream analyses, such as two-sample MR studies. CheckSumStats leverages three groups of SNPs to perform its check: a 1000 Genomes reference set, GWAS catalog associations, and the test GWAS top hits. By extracting summary data for these SNP groups from the target GWAS, CheckSumStats can confirm the identity of the effect allele frequency and effect allele columns, identify errors or analytical issues in the summary dataset, and infer the study's ancestry. The package aims to enhance the integrity of collated summary data prior to analysis, thereby increasing the reliability of post-GWAS analyses. To make CheckSumStats more accessible to researchers, we developed a web-based application that allows users to directly upload their data for quality control analysis easily.

The Q-Q plot is a graphical representation of the deviation of the observed *P*-values from the null hypothesis: the observed *P*-values for each SNP are sorted from largest to smallest and plotted against expected values from a theoretical χ^2-distribution. Additionally,

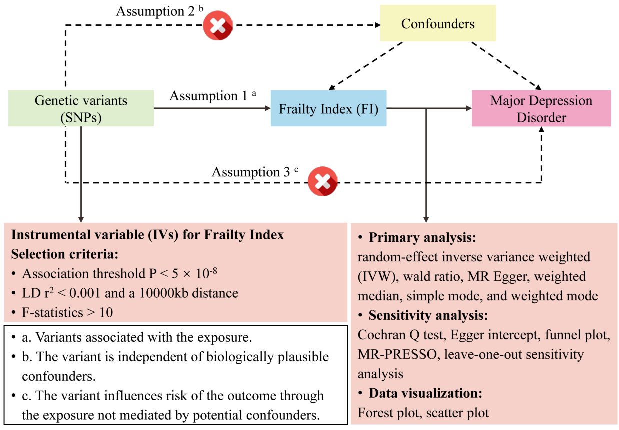

Figure 1. Workflow of the current two-sample Mendelian randomization (MR) study revealing causality from frailty index on major depression disorder. SNPs, single nucleotide polymorphisms; LD, linkage disequilibrium, MR-PRESSO, MR pleiotropy residual sum and outlier.

a Manhattan plot represents the *P*-values of the entire GWAS on a genomic scale, and it is normally used to check for consistency and to identify spurious associations. In a Manhattan plot, the *P*-values are represented in genomic order by chromosome and position on the chromosome (x-axis). The value on the y-axis represents the -log10 of the *P*-value. In this case, we also use Q-Q plot and Manhattan plot applications in MRanalysis to perform these two analyses directly.

Two-sample Mendelian randomization

To validate the accuracy of our platform, we replicated the findings of causality between major depression disorder (MDD) and frailty index (FI) from Wang et al. [33] using our online platform. To investigate the causal relationship between MDD and FI, we obtained GWAS summary statistics for MDD (n = 500,199) and FI (n = 175,226) from previously published studies, serving as exposure and outcome datasets, respectively (Table 1). The SNPs associated with the exposures at genome-wide significance ($P < 5 \times 10^{-8}$) were selected as instrumental variables (IVs) with F-statistics all greater than 10, satisfying MR assumptions (Figure 1). To ensure the independence of these IVs, we pruned them for linkage disequilibrium (LD) using LD clumping (r^2 < 0.001, distance = 10,000 kb).

In the MR analysis, we employed the random-effect inverse variance weighted (IVW) method as the primary analysis to study the causality between MDD and FI. We performed the Cochran's

Q test to assess the heterogeneity. To evaluate the robustness of the MR estimates, we compared the IVW approach with other MR methods, including Wald ratio, simple mode, MR Egger, weighted median, and weighted mode. We also utilized the intercept term derived from MR-Egger and MR-PRESSO to assess the horizontal pleiotropy. Leave-one-out analysis was also conducted to evaluate the sensitivity of MR results.

All the aforementioned steps of two-sample MR analysis can be performed online using our platform (both API and local modes) with default parameters. When removing confounding factors, we referred to the article by Wang et al. [33]. By leveraging our online platform, researchers can conveniently and without coding conduct two-sample MR analyses to explore the causal relationships between complex traits and diseases, providing important theoretical foundations and practical guidance for the development of disease prevention and treatment strategies.

All MR analyses in our platform were conducted using "TwoSampleMR" (version 0.6.8) and "MendelianRandomization" (version 0.9.0) packages in R software (version 4.4.1).

Multivariable Mendelian randomization

Mendelian randomization (MR) is a powerful approach for inferring causal relationships between exposures and outcomes using genetic variants (SNPs) as instrumental variables (IVs). It can be conducted using either individual-level data or summary data from GWAS, which provide the estimated effect of each SNP of exposure on the outcome. Multivariable Mendelian randomization (MVMR) extends the traditional MR framework by allowing for the estimation of causal effects of multiple exposures on the outcome, conditional on the other exposures included in the model. MVMR also can be used to evaluate mediating effects of an independent variable, to adjust for possible pleiotropy bias due to horizontal pleiotropy of a specific effect, or to adjust for potential confounding.

In this case, as shown in Figure 2, we performed a two-sample MVMR analysis using summary dataset from GWAS of HDL cholesterol, LDL cholesterol, Triglyceride, and Creatinine as exposures, and coronary heart disease (CHD) as outcome (Table 1). We selected SNPs that reached genome-wide significance ($P < 5 \times 10^{-8}$) in at least one of the exposure traits and pruned them for linkage disequilibrium (LD) using a pairwise r^2 threshold of 0.001 and 10,000kb distance. The resulting set of independent SNPs was then used as IVs in the MVMR analysis. We employed several MVMR methods, including multivariable MR-Egger, multivariable IVW, multivariable MR-Lasso, and multivariable median-based approaches, to estimate the causal effects of the exposures on the outcome. These analyses were conducted using the "MVMR" (version 0.4) and "MendelianRandomization" packages in R.

To assess the strength and validity of the IVs in the two-sample data, we used Cochran's Q statistical test to evaluate the robustness of results and ensure that the assumptions of MR were satisfied. By leveraging the power of MVMR and the wealth of summary data from large-scale GWAS, our applications provide valuable insights into the complex causal relationships between multiple exposures and the outcome of interest. The use of multiple MVMR methods and the assessment of IVs' strength and validity further strengthen the reliability of our tools.

Two-step or mediation Mendelian randomization

Mediation analysis is a powerful approach to study the underlying mechanisms through which an exposure affects an outcome. In the context of Mendelian randomization (MR), a two-step MR analysis can be employed to assess the potential role of a third variable (mediator) in the causal pathway between exposure and outcome. In this application the first step involves using genetic instrumental variables (IVs) associated with the exposure to determine the

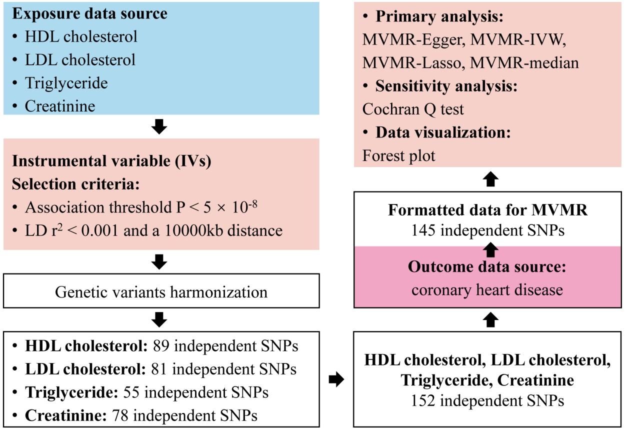

Figure 2. Workflow of the current Multivariable Mendelian randomization (MVMR) study revealing causality from HDL cholesterol, LDL cholesterol, Triglyceride, and Creatinine on coronary disease. SNPs, single nucleotide polymorphisms; LD, linkage disequilibrium; HDL, high-density lipoprotein; LDL, low-density lipoprotein; IVW, inverse variance weighted.

causal effect of the exposure on the potential mediator. The second step then utilizes IVs associated with the potential mediator, independent of those used in step one, to estimate the effect of the mediator on the outcome of interest. Methods such as the product-of-coefficients can be applied to quantify the extent of mediation. Importantly, the MR assumptions must be satisfied for both steps of the analysis: (i) exposure on mediator and (ii) mediator on outcome.

In this case, we conducted a four-step two-sample MR analysis to evaluate the complex relationships among feeling lonely, major depression disorder in trauma-unexposed individuals (MDD), and cigarettes smoked per day (Table 1). Step 1 involved an MR analysis of feeling lonely on MDD, while step 2 examined the reverse causal relationship between feeling lonely and MDD. These two steps were combined into a bidirectional MR analysis to explore the potential primary and reverse causal relationships between feeling lonely and MDD. Step 1, 3, and 4 were then integrated into a two-step MR mediation analysis to assess the potential mediating role of cigarettes smoked per day in the relationship between feeling lonely and MDD. Specifically, step 3 investigated the causal effect of feeling lonely on cigarettes smoked per day, and step 4 evaluated the causal effect of cigarettes smoked per day on MDD. In this mediation analysis, beta0 represents the total effect, while beta1 and beta2 represent the direct effects of feeling lonely on cigarettes smoked per day and cigarettes smoked per day on MDD, respectively (Figure 3). The specific calculation method (product of coefficients) for the mediating effect is also shown in Figure 3.

In the first step, we evaluated the causal relationship between exposure and mediation variables (beta1). Subsequently, in the second step, we estimated the causal effect of mediators on outcomes through multivariate MR (beta2). We then calculated the total effect (beta0) between exposure and outcome using two-sample MR analysis. When beta0, beta1, and beta2 were all significant, a causal relationship existed between the outcome and exposure, and the mediating variable played a partial mediational role in this causal relationship. The mediating effect was calculated using beta1*beta2, while the mediating proportion of the causal effect between exposure and outcome was calculated using (beta1*beta2)/beta0. Finally, we estimated the proportion of the mediation effect in the total effect using the delta method. We calculated the odds ratios (OR) and 95

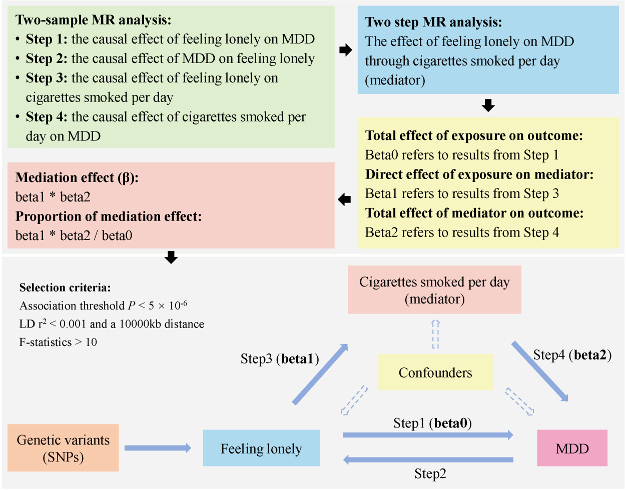

Figure 3. Flowchart of two-step Mendelian randomization analysis revealing the mediating effect of cigarettes smoked per day on the risk of MDD through feeling lonely. MR, Mendelian randomization; MDD, major depressive disorder in trauma-unexposed individuals; SNPs, single nucleotide polymorphisms.

SNP gene mapping and enrichment

Gene and gene-set analysis are powerful statistical approaches that enable researchers to investigate the combined effects of multiple genetic markers on complex, polygenic traits. These methods are particularly useful when individual markers have weak effects that are difficult to detect using traditional single-marker analysis. Furthermore, gene-set analysis can provide valuable insights into the functional and biological mechanisms underlying the genetic component of a trait. While several methods for gene and gene-set analysis are available, they often suffer from various statistical issues and can be computationally intensive. To address these challenges, De Leeuw et al. [34] have developed a new method called MAGMA, which offers improved statistical power and computational efficiency compared to existing methods. To make MAGMA more accessible and user-friendly, we have developed a Shiny application that allows users to directly upload their data for this analysis. This application streamlines the process of conducting gene and gene-set analysis, making it easier for researchers to explore the genetic basis of complex traits.

The genes identified through the aforementioned mapping process were subsequently analyzed using our MRanalysis platform. The platform conducted Gene Ontology (GO) and Kyoto Encyclopedia of Genes and Genomes (KEGG) enrichment analyses based on the clusterProfiler (v4.12.6) R package [35], with default parameters applied.

Power and sample size estimation

For power and sample size estimation, we used the PowerCalculator and SampleSizeCalculator applications integrated within the MRanalysis platform to determine statistical power and the minimum required sample size for three types of MR analyses: two-sample univariable MR (2SMR), multivariable MR (MVMR), and mediation MR (MMR). Because the tools do not directly support power and sample size calculations for MVMR and MMR, we decomposed each multivariable or mediation analysis into exposure–outcome components and estimated sample sizes separately. For each computation, only one of "sample size" or "power" was specified and the other was computed by the tool. In Case VIII, we applied the MR analyses described above and, for each exposure–outcome pair, computed the minimum required sample size individually. The minimum detectable effect size with the current samples, assuming 80% power and a two-sided significance level of 0.05, was obtained using the SampleSizeCalculator application, which implements code from Burgess [36]. Required inputs included the proportion of variance in the exposure explained by the selected genetic instruments (R^2), the anticipated causal effect size (odds ratio per standard deviation for binary outcomes or standard deviation change per standard deviation for continuous outcomes), the significance level (α), and, for binary outcomes, the case-to-control ratio. For two-sample analyses, the sample size was defined as the number of participants in the variant–outcome association dataset.

Results

Overall design and workflow of MRanalysis

MRanalysis is a comprehensive web-based platform designed to streamline and standardize Mendelian Randomization (MR) analysis and preprocess GWAS summary dataset. By leveraging the extensive interactive features of the R Shiny framework, MRanalysis provides a wide range of interactive functionalities, from handling various GWAS data file formats (e.g., CSV, TSV, VCF) to performing data extraction, format standardization, rs ID mapping, gene mapping, enrichment analysis, data visualization, and supporting several common MR approaches. To enhance user experience and facilitate easier adoption of our platform, we have prepared sample datasets for each application. These example datasets allow users to familiarize themselves with the platform's functionalities and test its features before using their own data. Moreover, we have created comprehensive animated GIFs for each application, visually illustrating the key steps of the process. These animated guides provide a clear, step-by-step visual representation of how to navigate the platform and utilize its various tools. The overall structure of our platform can be divided into three main components: the analysis section (containing common MR approaches), the plugin section (encompassing various post-GWAS/MR methods), and the visualization section. The analysis section is the core component of MRanalysis, where researchers can conveniently perform univariable, multivariable, and mediation MR in both local and API modes.

When users want to perform an MR analysis, the overall pipeline can be divided into 5 main stages: data preprocessing, quality control (QC), MR analysis, post-MR analysis and data visualization (Figure 4). Taking local two-sample MR analysis (univariable) as an example, (1) in the data preprocessing stage, users can utilize the GWASkit standalone tool to efficiently handle tasks such as format conversion (e.g., VCF to TSV, or TSV to VCF), rs ID mapping (converting chromosome, base pair location, effect and non-effect allele information to SNP IDs), and GWAS data format standardization. GWASkit's "pos2rs" and "rs2pos" subcommands specifically cater to mapping between rs IDs and their coordinates, offering advantages of being pre-installed, multi-platform, efficient, highly accurate, and fast compared to other existing tools. (2) The QC stage is crucial before conducting MR analyses. Our platform provides three key functions: CheckSumStats for identifying allele frequency conflicts and metadata errors in GWAS datasets, Manhattan plot for visualizing genetic associations and significance levels for multiple SNPs, and Q-Q plot for assessing the distribution and overall characteristics of GWAS data. These features ensure the quality and reliability of the data before proceeding with MR analyses. (3) In the analysis stage, MRanalysis offers three mainstream MR approaches, including Two-Sample univariable MR, Multivariable MR, and Mediation MR, through its "Analysis" module and associated plugins. Users can easily conduct these analyses using either API mode for searching GWAS data online or local mode for uploading their own GWAS data. (4) After MR analysis, we can annotate instrumental variables and conduct gene enrichment analyses, including GO and KEGG. This process enhances biological interpretation of MR results, potentially revealing functional pathways underlying causal

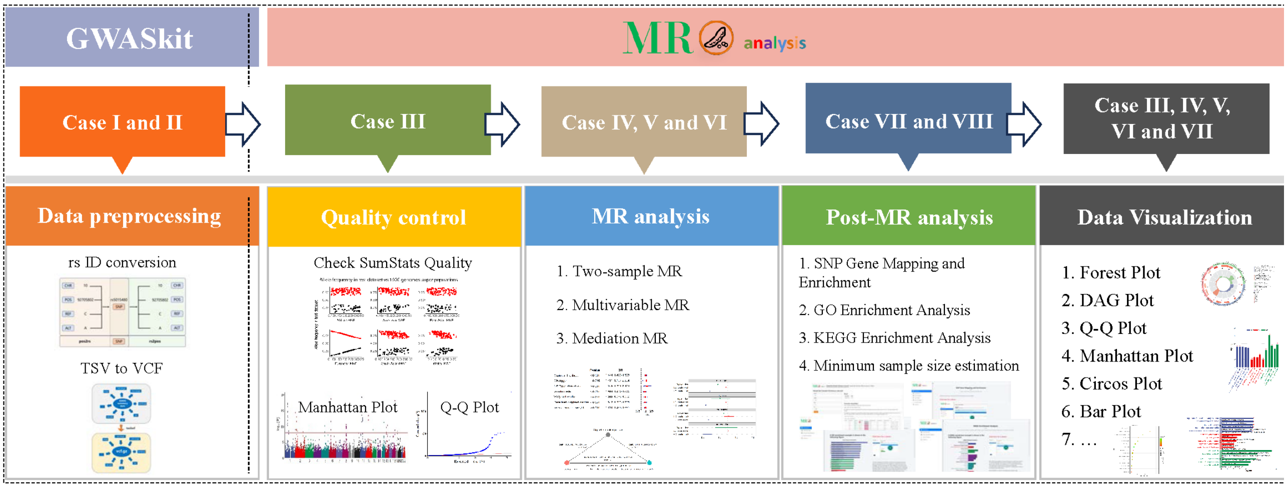

Figure 4. Overview of MRanalysis design.

relationships. (5) At last, the results can be visualized using our plot applications, such as, forest plot, DAGs (Directed Acyclic Graphs), bar plot, dot plot and circos plot to facilitate intuitive interpretation and presentation of the findings.

To further enhance the robustness and reliability of MR analyses, MRanalysis also provides several additional utilities, including a Power Calculator application for estimating the statistical power of MR studies and a Sample Size Calculator application for determining the minimum sample size required for a given power [37]. The power of an MR study is determined by the sample size and the strength of the association between the proposed instruments and the risk factor [38]. Users can also perform SNP to gene annotation and enrichment analysis using the corresponding plugins (e.g., MAGMA application [34] within our platform directly.

In summary, MRanalysis coupled with the GWASkit, presents a complete and user-centric solution for researchers to efficiently handle GWAS data preprocessing, QC, MR analyses and visualization, as well as perform enrichment analyses. The platform's modular structure and extensive capabilities render it a crucial resource for researchers, particularly those with limited coding skills, to address the intricacies of MR analysis and elucidate causal relationships between genetic variants and phenotypes of interest. It is worth noting that each application within the platform provides complete code generation capabilities, enabling local reproduction of results. Through this code, users can clearly understand the entire analysis process and support further personalized analyses. The seamless integration of the three main sections within MRanalysis highlights its value and utility in MR, providing an accessible, versatile, and potent platform for scientists to overcome the challenges associated with GWAS data processing and MR studies.

Case I: rs ID mapping

Single nucleotide polymorphisms (SNPs) are genomic locations known to vary between individuals. The rs ID number is a unique identifier ("rs" followed by a number, e.g. rs12306) used by researchers and databases to designate a specific SNP. This naming convention, which stands for Reference SNP cluster ID, is widely used for most SNPs. When researchers identify a SNP, they send a report containing the sequence surrounding the SNP to the dbSNP database. Submitted variants are categorized, organized, and annotated, with duplicate variants being consolidated. Unlike the CHR-POS identifier, which changes with different reference genome versions while rs ID remains consistent across versions. This consistency provides a stable method for representing variants, making it more suitable for large-scale studies in population genetics or precision medicine.

GWASkit pos2rs provides functions to convert CHR-POS-REF-ALT (chromosome, base pair locations, non-effect (other or reference) allele and effect allele) to rs ID using reference files downloaded from the NCBI dbSNP database, or self-prepared files for rapid conversion.

To evaluate the performance of rs ID conversion by GWASkit and other existing tools, we used a GWAS summary data downloaded from the GWAS Catalog as test data. Figure 5A illustrates the run time for rs ID conversion using GWASkit and six other existing tools (ANNOVAR [39], snpEff [40], MungeSumstats [41], gwaslab [42], NCBI dbSNP database and SNPnexus [43]). In comparison, GWASkit required the shortest time to complete the rs ID conversion, taking only 0.24 hours (14.40 minutes), while ANNOVAR, MungeSumstats, and gwaslab require 0.42, 0.38, and 8.01 hours, respectively (Figure 5A). Meanwhile, snpEff required the longest time, 20.67 hours. It is important to note that snpEff is a genetic variant annotation and functional effect prediction toolbox, which simultaneously annotates many other pieces of information, resulting in a more time-consuming process. In terms of RAM usage, ANNOVAR and snpEff required similar amounts of memory consumption, but much less than MungeSumstats (Figure 5B). The high memory requirement of MungeSumstats is likely due to the R compiler's tendency to use more memory. In comparison, GWASkit uses a moderate amount of memory, 22.00GB, primarily to achieve better performance. We also tested GWASkit on a Windows computer with 8GB of available memory, and it worked normally, although the runtime was extended by approximately 1 hour.

When comparing different tools from various perspectives (Table 2), we find that the choice of tool should be based on specific usage scenarios. For batch processing needs, local tools are more recommended due to their efficient processing capabilities; conversely, if only several SNPs need to be annotated, direct use of some online tools, such as the NCBI dbSNP database or SNPnexus, is sufficiently convenient. Considering that GWAS summary data is usually very large, often reaching hundreds of megabytes (MB) or even 1 or 2 gigabytes (GB), the advantages of local tools for processing such large data are particularly evident (Figure 5). It is worth noting that most of these tools are developed based on the Linux operating system or require certain programming skills, posing some requirements for users. From the user's perspective, accuracy is the primary indicator for evaluating tool performance, while the simplicity of tool installation and ease of use are also important, as they directly set a threshold that limits more researchers from using it. Regarding accuracy, our tool GWASkit significantly outperforms other tools with its extremely high accuracy rate (99.999993%, close to 100%, Table 2), followed by ANNOVAR and snpEff with

Table 2. Feature comparisons of currently available rs id conversion tools (local and web tools)

Tool	Type	Platforms	Standalone	User Interface	Batch	Accuracy(%)
GWASkit	local	Linux, Windows	Yes	UI for Windows	Yes	100.0
ANNOVAR	local	Linux	Perl Scripts	No	Yes	97.25
snpEff	local	Linux	JAR file	No	Yes	98.87
MungeSumstats	local	Linux, Windows	R package	No	Yes	59.67
gwaslab	local	Linux, Windows	Python module	No	Yes	98.35
NCBI dbSNP	online	-	-	Yes	No	-
SNPnexus	online	-	-	Yes	Yes[a]	-

a. SNPnexus limits the maximum number of variants in a single batch query to 10,000. UI: user interface.

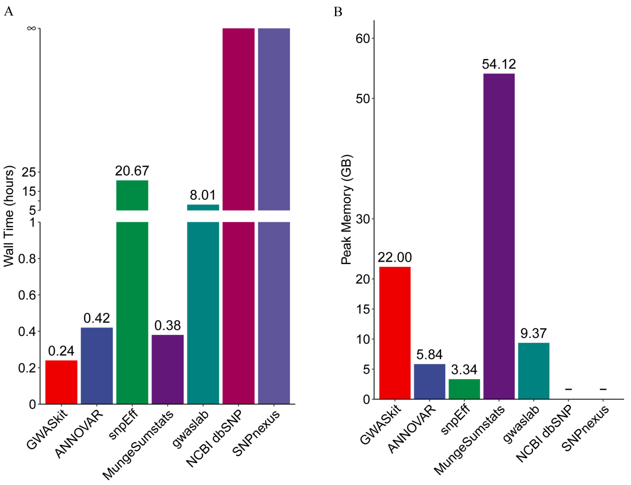

Figure 5. Run time (A) and peak memory consumption (B) by different tools. Test data: a large-scale GWAS summary data (GCST90236305) downloaded from the EMBL-EBI GWAS Catalog database, which contained 14,519,897 variants with complete information, including chromosome, location, other allele, effect allele, and rs ID details. Test environment: Linux® Ubuntu 20.04.4 LTS operating system, equipped with a 16-core Intel® (CPU), 128GB of RAM, and a 12TB hard drive.

accuracy rates of 97.25% and 98.87%, respectively. In contrast, MungeSumstats has the lowest accuracy rate of only 59.67%, which somewhat limits its application in high-accuracy scenarios.

Overall, GWASkit not only stands out with its excellent accuracy but is also particularly suitable for researchers with limited or even no programming skills. GWASkit is a standalone binary executable file that avoids complex installation steps and supports multiple operating systems (Table 2). Especially with its Windows version, which features a simple user interface (UI), users can complete tasks that typically require programming skills and complex installation by just clicking and entering necessary information. This greatly lowers the usage threshold and enhances efficiency. Additionally, using GWASkit for rs ID conversion can be done with just a single command.

Case II: GWAS summary data standardization

The VCF (Variant Call Format) is a standard text file format widely used in bioinformatics for storing gene sequence variations. VCF files facilitate the integration of GWAS summary data with other genomic datasets by providing metrics and filters that ensure only reliable variants are considered. The standardized format allows them to be used across different tools and platforms, making VCF files a versatile choice for researchers. GWASkit provides functions for standardizing the format of GWAS summary data, ensuring that datasets from a wide range of sources are as interoperable as possible. Most importantly, for local MR analysis applications (e.g, Two-sample MR analysis), reading a large-scale GWAS summary data with R scripts typically requires a significant amount of memory and is time-consuming. For example, processing a 500MB GWAS summary data locally might use 8 to 10GB of memory and take about 20 minutes. To address this, MRanalysis has optimized the process by using standard VCF format files for local analysis, reducing the time to just 2 to 3 minutes and requiring only 1 to 2 GB of memory.

To illustrate the versatility of GWASkit in converting GWAS statistics from various formats into the standardized VCF format, we provided a comprehensive walkthrough using the MDD and FI data from Table 1. The MDD data posed a challenge, as it only included rs ID information, lacking some crucial details such as chromosome number and base pair location. To address this issue, we employed the "rs2pos" subcommand of GWASkit, which efficiently filled the missing information. The process took approximately 12.93 minutes and had a peak memory usage of 17.79GB. Upon completion of the data augmentation, we proceeded to utilize the 'tsv2vcf' subcommand to perform a standardization of the data. This step required 3.27 minutes and had a peak memory usage of 6.18GB. In contrast, the FI dataset already contained all the necessary information, allowing us to directly apply the "tsv2vcf" for standardization, which also took about 3 minutes and had a peak memory usage of 7.33GB. By successfully converting the MDD and FI GWAS data into standardized VCF format, we prepared them for subsequent MR analysis.

GWASkit's robust data processing and format conversion capabilities offer researchers an efficient and user-friendly tool to tackle GWAS data in various formats from various databases. The streamlined workflow provided by GWSAkit, from data augmentation to standardization, not only saves time and computational resources but also ensures data consistency and compatibility across different platforms. This standardization is particularly crucial for large-scale meta-analysis and collaborative research efforts, where data from multiple sources need to be integrated and analyzed together. Moreover, the reduced memory usage and processing time achieved by MRanalysis using standardized VCF files significantly enhance the accessibility and feasibility of local MR analyses. This optimization enables researchers with limited computational resources to perform complex analyses on their own machines.

Case III: GWAS summary data quality control

The comparison of allele frequencies between the frailty index (FI) GWAS dataset and the 1000 Genomes reference population data allows for a systematic examination of the accuracy and consistency of the genetic data, establishing a solid foundation for subsequent genetic statistical analyses. In this case, we used the CheckSumStats application to intuitively present the distribution patterns of SNP allele frequencies in the two datasets. As shown in Figure 6A, black points represent SNP loci with consistent frequencies, while red points represent SNP loci with frequency conflicts. The vast majority of SNPs have frequencies less than 0.5 and are consistent between the two datasets, suggesting that the reported effect allele

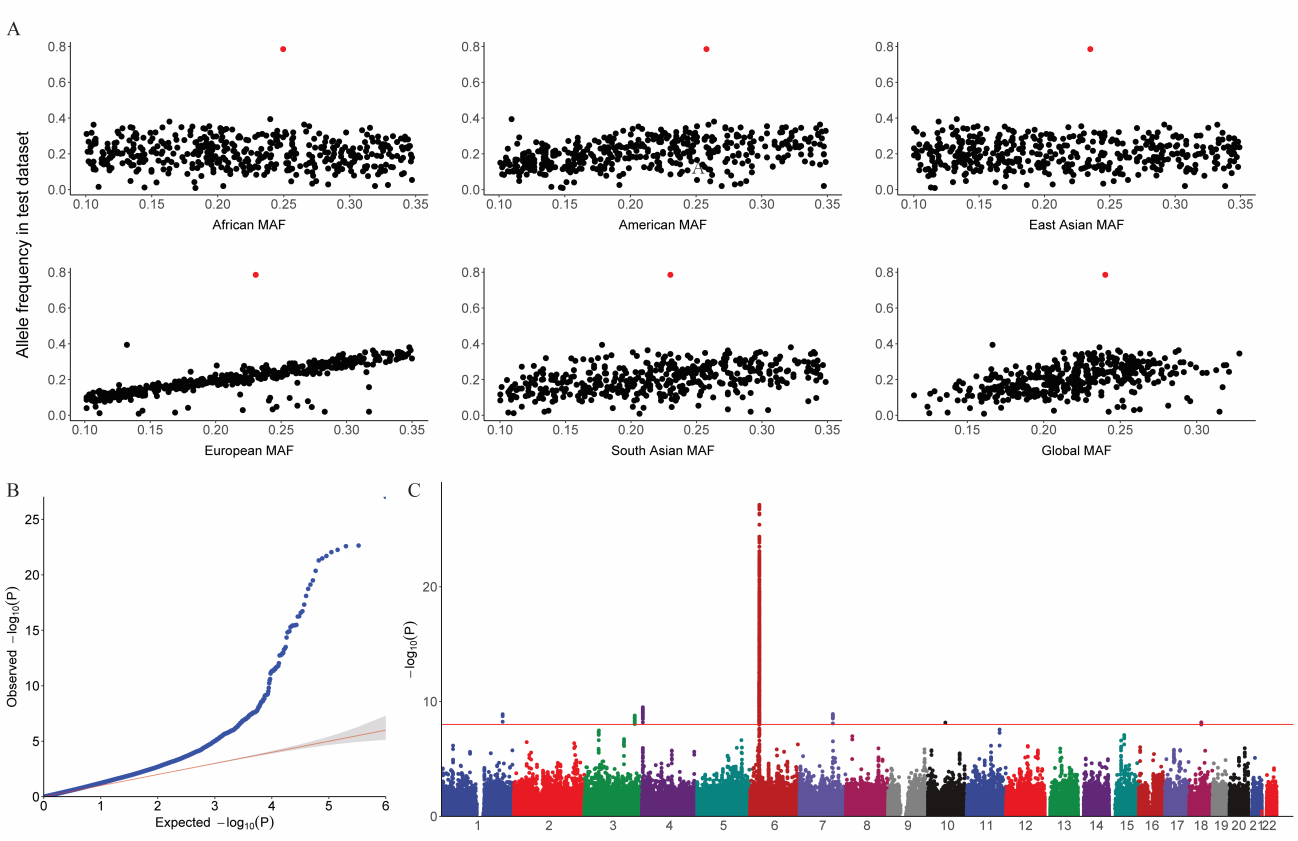

Figure 6. Quality control of GWAS summary data of frailty index. (A) Allele frequency in test dataset (frailty index) vs 1000 genomes super populations. Allele frequencies are expected to be < 0.50 (black points). A high allele frequency conflict is defined as an allele frequency of > 0.58 (red points) in the test data or if the allele frequency differs by > 10 points between the test and reference data sets. Moderate allele frequency conflicts are allele frequencies of > 0.50 but ≤ 0.58; (B) Q-Q plot of the GWAS dataset. The observed results deviate significantly from the expected results, suggesting that the effects of these loci exceed random effects and may be significantly associated with the phenotype; (C) Manhattan plot of the GWAS dataset. The horizontal axis represents the genomic locations of all the tested SNP in physical order. The vertical axis shows -10log *P* values for individual variant association with frailty index. Red lines indicate the threshold for genome-wide significance at 5×10^{-8}.

frequencies in this case dataset can accurately correspond to the effect alleles themselves. However, a considerable proportion of SNPs exhibit frequency conflicts, and the speculated reason may be that the effect allele frequency column confuses effect alleles and non-effect alleles, actually recording the minor allele frequency. The identification of allele frequency conflicts underscores the importance of careful data curation and quality control in genetic association studies. Misclassification of effect and non-effect alleles can introduce biases and lead to erroneous conclusions. Therefore, it is crucial to implement robust data cleaning and validation procedures to ensure the accuracy and reliability of the dataset before conducting downstream analyses.

The Q-Q plot (Figure 6B) showed that the observed results deviated significantly from the expected results at lower *P*-values, suggesting that the effect of these loci exceeds random effects and might be significantly associated with the phenotype. This deviation from the expected distribution indicates the presence of true associations between the genetic variants and the FI, warranting further investigation into the biological mechanism underlying these associations. The Manhattan plot (Figure 6C) revealed that a SNP cluster on chromosome 6 showed the most significant association with the FI. This finding highlights the potential importance of genetic variants in this region in influencing an individual's susceptibility to frailty. In conclusion, the CheckSumStats, in conjunction with the Q-Q plot and Manhattan plot, provides a comprehensive assessment of the GWAS datasets.

Case IV: Two-sample Mendelian randomization

In this case, the findings of causality between major depression disorder (MDD) and frailty index (FI) of Wang et al. [33] were replicated using our web platform. Two approaches were employed: (1) using the local mode of two-sample MR analysis with standardized GWAS dataset (Case I, II and III); (2) directly utilizing the API mode application to access online data from the IEU OpenGWAS project. Remarkably, the results obtained from both approaches were nearly identical, demonstrating the robustness and reproducibility of the findings. Some minor discrepancies observed between the two approaches can likely be attributed to the personalized processing and curation steps implemented by IEU OpenGWAS during their data cleaning and preprocessing pipeline.

In this replication study, a total of 3,008 genetic variants associated with major depression disorder (MDD) reached genome-wide significance ($P < 5 \times 10^{-8}$) (Figure 7A). Of these, 50 SNPs were selected as the instrumental variables (IVs). The F-statistics for the IVs ranged from 29.7519 to 78.4487, all exceeding the threshold of 10, indicating that the IVs were not biased by weak instruments. This ensures the validity of the selected genetic variants as robust IVs of MDD in the Mendelian randomization (MR) analysis. The inverse variance weighted (IVW) analysis showed that the genetic changes in the MDD were statistically associated with an increased risk of frailty index (FI) (local mode: OR = 1.256, 95% CI: 1.192-1.323, $P < 0.001$, Figure 7B; API mode: OR = 1.256, 95% CI: 1.192-1.324, $P < 0.001$, Figure 7D), with some heterogeneities was observed among IVs (Q = 73.3864 and 74.5128 in local and API mode, respectively, and $P < 0.001$ both in two modes). The causality between MDD and FI was also further confirmed by other MR methods, including the MR

Egger, weight median, simple mode and weight mode (Figure 7B and D). The scatter plot and trend line showed the consistent trend of causal relationship between MDD and FI for all five MR methods (Figure 7C and E). To assess the presence of horizontal pleiotropy, which can bias the MR estimates, the MR Egger intercept test and MR-PRESSO distortion test were performed. Both tests showed no indication of horizontal pleiotropy (all *P* values greater than 0.05), supporting the validity of the MR assumptions and the reliability of the causal estimates.

The consistency of results across different data sources and processing methods highlights the reliability and validity of the MRanalysis platform in conducting MR analyses. By offering both local and API-based data integration options. MRanalysis provides researchers with the flexibility to choose the most suitable approach based on their specific requirements and data availability. This versatility ensures that researchers can conduct MR analyses using the most appropriate and up-to-date data sources while maintaining the integrity and comparability of the results.

Case V: Multivariable Mendelian randomization

In this case, we investigated the potential causal relationship between creatinine and coronary heart disease (CHD), considering the possibility that instrumental variables (IVs) for creatinine could act through lipid species like triglycerides to influence the risk of CHD. To account for this potential mediation, we constructed multivariable Mendelian randomization (MVMR) models that estimate the creatinine to CHD relationship conditioned on HDL cholesterol, LDL cholesterol, and triglycerides.

To further investigate the independent effects of creatinine and major lipid species on CHD, we screened 145 SNPs as IVs for the MVMR. The MVMR-IVW method showed that triglycerides (OR = 1.16, 95% CI: 1.04-1.30, $P = 0.010$) and LDL cholesterol (OR = 1.33, 95% CI: 1.21-1.45, $P < 0.001$) were significantly associated with the risk of CHD. In contrast, HDL cholesterol (OR = 0.93, 95% CI: 0.85-1.02, $P = 0.135$) and creatinine (OR = 1.06, 95% CI: 0.85-1.32, $P = 0.600$) were not significantly associated with the risk of CHD (Figure 8). These results suggest that higher levels of triglycerides and LDL cholesterol may causally contribute to the development of CHD, independent of the effects of creatinine and HDL cholesterol. The consistency of the results across the remaining four MVMR methods further strengthens the reliability of these findings. The agreement between different MR methods, each with its own assumptions and robustness properties, provides additional confidence in the conclusions drawn from the analysis.

Case VI: Two-step or mediation Mendelian randomization

This case employs a four-step two-sample Mendelian randomization (MR) approach to investigate the complex relationships between feeling lonely, major depressive disorder in trauma-unexposed individuals (MDD), and cigarette smoked per day. The bidirectional MR analysis provides insights into the potential causal directionality between feeling lonely and MDD, while the two-step mediation MR analysis explores the potential role of cigarettes smoked per day as mediator in this relationship. The result of inverse variance weighted (IVW) method shows that the genetic changes in the feeling lonely were statistically associated with the risk of MDD (OR = 1.67, 95% CI: 1.06-2.63, $P = 0.026$, Figure 9A), however the SNPs change in the MDD were not significantly associated with feeling lonely (OR = 0.99, 95% CI: 0.98-1.00, $P = 0.355$, Figure 9A), indicating that the causal relationship between feeling lonely and MDD may be unidirectional. In the analysis of the exposure-mediator relationship, the IVW results indicate a positive causal relationship between feeling lonely and cigarettes smoked per day (OR = 1.31, 95% CI: 1.12-1.52, $P < 0.001$, Figure 9A). This finding suggests that individuals who feel lonely may be more likely to engage in smoking behavior, potentially as a coping mechanism or due to shared underlying factors. Furthermore, for mediator to outcome relationship, IVW results suggest that cigarettes smoked per day significantly increase the risk of MDD (OR = 1.18, 95% CI: 1.03-1.34, $P = 0.016$, Figure 9A).

Based on the findings of step 1 (feeling lonely to MDD), step 3 (feeling lonely to cigarettes smoked per day) and step 4 (cigarettes smoked per day to MDD), the mediating role of cigarettes smoked per day in the relationship between feeling lonely and MDD has been demonstrated (Figure 9B). The mediating effect of cigarettes smoked per day in increasing the risk of MDD through feeling lonely was found to be statistically significant ($\beta = 0.044$; 95% CI: 0.0003-0.0868, $P = 0.049$). The mediation proportion, indicating the proportion of the total effect of feeling lonely on MDD that is mediated by cigarettes smoked per day, was estimated to be 8.47

Case VII: SNP gene mapping and enrichment

In case V, a multivariable Mendelian randomization analysis was used to study the potential causal effects of creatinine and major lipid species on CHD. To further study the functional implications of the genetic variants associated with CHD, the MAGMA plugin was employed to annotate the 146 instrumental variables (IVs) to their corresponding genes. This annotate resulted in a set of 99 genes. MAGMA uses MSigDB by default for enrichment analysis, while its extensibility allows seamless integration with external bioinformatics tools to further explore the functional impact of identified genes. In this case study, we leveraged MAGMA's capabilities to map SNPs to their corresponding genes. This crucial step bridges the gap between genetic variants and their potential functional impacts at the gene level. Following this mapping process, we conducted enrichment analyses using applications of GO and KEGG enrichment analysis. To enhance the interpretability and visual representation of our results, we imported the enrichment analysis outputs into the visualization module of MRanalysis. This visualization module offers a suite of powerful visualization tools, including circos plots, bar plots, and dot plots (Figure 10). These enrichment analyses provide valuable insights into the biological processes (BP), molecular functions (MF), and cellular components (CC) associated with the identified genes, as well as their involvement in various biological pathways in CHD.

MAGMA plugin is a powerful application that combines robust statistical methodology with an intuitive user interface, enabling researchers to uncover novel insights into the genetic architecture of complex traits. By integrating genetic association data with functional genomic annotations, MAGMA/GO/KEGG applications facilitate the identification of biologically meaningful gene sets and pathways, ultimately advancing our understanding of the underlying biological mechanisms. By integrating genetic association data with functional genomic annotations and enabling seamless integration with external bioinformatics tools, enrichment analysis empowers users to uncover novel insights into the underlying biological mechanisms.

Case VIII: Minimum sample size estimation

Using the MRanalysis SampleSizeCalculator, we estimated the minimum total sample size required to achieve 80% power at $\alpha = 0.05$ for each exposure–outcome pair and compared these thresholds with the available GWAS outcome sample sizes. Five of nine analyses met or exceeded the predicted minimum and were therefore adequately powered at the observed effect sizes, while four were underpowered. Specifically, MDD - frailty index (N_available = 175,226 vs N_required = 52,600; 3.3×), feeling lonely - MDD (500,199 vs 203,700; 2.5×), MDD - feeling lonely (376,352 vs 41,900; 9.0×), LDL cholesterol - coronary heart disease (184,305 vs 4,200; 44×), and triglycerides → coronary heart disease (184,305 vs 25,100; 7.3×)

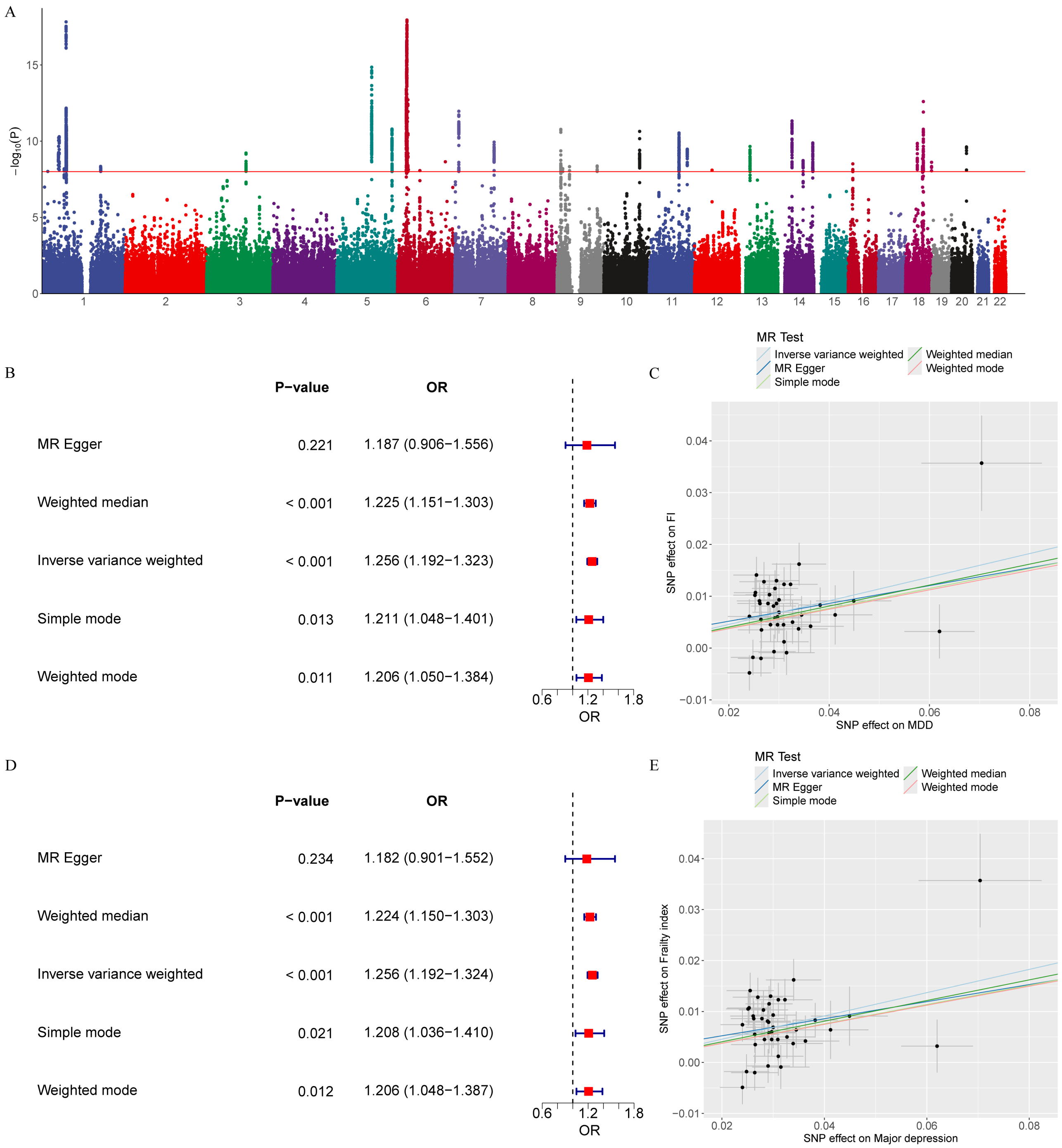

Figure 7. The results of 2SMR analysis. (A) Manhattan plot showing distribution of *P*-values from genome-wide association study of MDD. (B) Forest plot of MR analysis of the MDD and FI using local mode. (C) Individual estimates about the effect of MDD on FI using local mode. The X-axis shows the SNP effect and SE on each of the instrumental variables of MDD. The Y-axis shows the SNP effect and SE on FI. Analyses were conducted by using MR Egger, weighted median, inverse variance weighted, simple mode and weighted mode. The slope of each line corresponds to the estimated MR effect per method. (D) Forest plot of MR analysis of MDD (ieu-b-102) and FI (ebi-a-GCST90020053) using API mode. (E) Individual estimates about the effect of MDD on FI using API mode. 2SMR, two-sample Mendelian randomization; MDD, major depression disorder; FI, frailty index; SNP, single nucleotide polymorphism; API, application programming interface; OR, odd ratio; SE, standard error.

were all adequately powered. In contrast, feeling lonely - cigarettes smoked per day (249,752 vs 3,250,800; 0.08×), cigarettes smoked per day - MDD (500,199 vs 1,241,241,600; 0.0004×), HDL cholesterol - coronary heart disease (184,305 vs 538,500; 0.34×), and creatinine - coronary heart disease (184,305 vs 2,513,800; 0.07×) were underpowered, with the smoking-to-MDD analysis particularly constrained by an effect estimate near the null (log OR $\approx$ 0.0014; OR $\approx$ 1.0014), implying an unrealistically large required sample size, (Table 3).

These comparisons indicate that for adequately powered pairs, the minimum detectable effect size given the available sample size was smaller than the estimated causal effect, whereas for underpowered pairs, it exceeded the observed magnitude. For binary outcomes, calculations accounted for the case–control composition of the corresponding GWAS (case fractions 0.49–0.52, as reported). In two-sample analyses, the outcome sample size was defined as the number of participants contributing to the variant–outcome association estimates, in accordance with the calculator's conventions.

Table 3. Feature comparisons of currently available rs id conversion tools (local and web tools)

Exposure	Outcome	Outcome Type	rsq[a]	b1[b]	OR[c]	ratio[d]	Actual[e]	Estimated[f]
ieu-b-102	ebi-a-GCST90020053	continuous	0.0038	0.1976	1.2185	-	175226	52600
ebi-a-GCST006942	ieu-b-102	binary	0.0036	0.2187	1.2445	0.5183	500199	203700
ieu-b-102	ebi-a-GCST006942	continuous	0.0097	0.1393	1.1494	-	376352	41900
ebi-a-GCST006942	ieu-b-142	continuous	0.0037	0.0255	1.0258	-	249752	3250800
ieu-b-142	ieu-b-102	binary	0.0139	0.0014	1.0014	0.5183	500199	1241241600
ieu-a-299	ieu-a-7	binary	0.0537	0.0350	1.0357	0.4923	184305	538500
ieu-a-300	ieu-a-7	binary	0.0503	0.4110	1.5084	0.4923	184305	4200
ieu-a-302	ieu-a-7	binary	0.0473	0.1731	1.1890	0.4923	184305	25100
met-d-Creatinine	ieu-a-7	binary	0.0358	-0.0199	0.9803	0.4923	184305	2513800

a. coefficient of determination (R^2) of exposure on genetic variants; b.causal effect; c. Odds Ratio; d. ratio of case:controls; e. actual sample size; f. estimated minimum sample size.

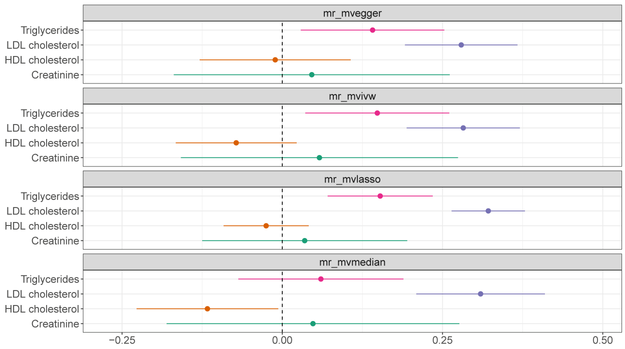

Figure 8. Multivariable Mendelian randomization (MVMR) models investigating the effect of creatinine and major lipid species on coronary heart disease. Each panel represents the results from a different MVMR model (each with different underlying assumptions). MR, Mendelian randomization; LDL cholesterol, low-density lipoprotein cholesterol; HDL cholesterol, high-density lipoprotein cholesterol; IVW, inverse variance weighted.

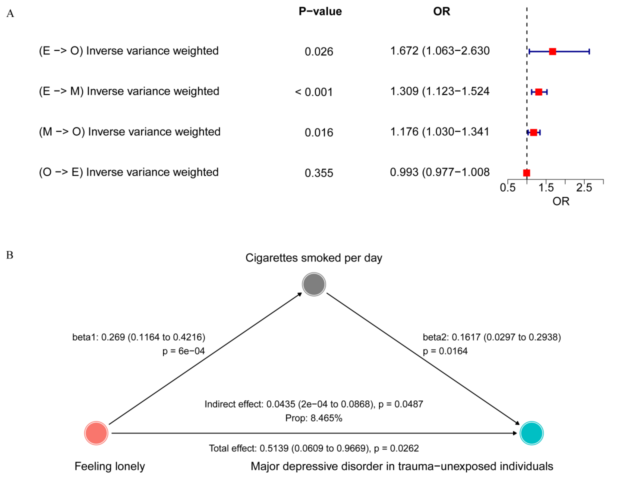

Figure 9. The results of mediation Mendelian randomization (MMR) analysis. (A) Forest plot of four-step two-sample MR approaches; (B) Directed acyclic graph (DAG) for the causal mediation analyses for the complex relationships between feeling lonely, major depressive disorder in trauma-unexposed individuals (MDD), and cigarettes smoked per day. E, exposure; O, outcome; M, mediation; MR, Mendelian randomization; DAG, directed acyclic graph; MDD, major depressive disorder in trauma-unexposed individuals; OR, odd ratio.

Discussion

MRanalysis and GWASkit provide a comprehensive and user-friendly platform for MR analyses and processing GWAS summary data. The development of our platform addresses several key challenges in the field, such as the complexity of different MR methods, the lack of standardized workflows, and the need for extensive coding experience. By integrating data preprocessing, QC, MR analysis and visualization, MRanalysis streamlines the entire MR workflows, making it more accessible to researchers with diverse backgrounds and varying levels of coding expertise (all applications in MRanalysis include test data and complete operation GIFs are recorded).

While MRanalysis democratizes MR methodology for researchers across diverse backgrounds, we acknowledge the critical importance of preventing methodological misuse. Increased accessibility raises legitimate concerns about atheoretical applications—particularly conducting cross-trait analyses without biological justification or examining arbitrarily selected exposure-outcome pairs. MR's validity as a causal inference framework fundamentally depends on satisfying core assumptions and establishing biologically plausible relationships. Users must ground analyses in well-defined, evidence-based hypotheses and adhere to established best-practice guidelines [22]. MRanalysis enforces comprehensive quality control standards including instrument strength evaluation (F-statistics), heterogeneity assessment (Cochran's Q), horizontal pleiotropy detection (MR-Egger intercept, MR-PRESSO), and mandatory power calculations. Violation of fundamental assumptions—relevance, independence, and exclusion restriction—particularly when selecting exposure-outcome pairs without considering biological plausibility or pleiotropic pathways, invariably yields spurious causal inferences. To facilitate methodologically sound research, MRanalysis incorporates automated quality checks, sample size calculators, transparent assumption reporting, and real-time analytical warnings. This integrated framework balances accessibility with methodological rigor, fostering responsible implementation while emphasizing that biological plausibility and methodological scrutiny remain paramount for valid causal inference in genetic epidemiology.

One of the major strengths of MRanalysis is the modular structure, which allows users to perform a wide range of analyses, from univariable and multivariable to mediation MR. Our platform also offers additional utilities, such as power and sample size calculators, SNP to gene annotation, and enrichment analysis, further enhancing the robustness and reliability of MR studies. The incorporation of best practices and guideline proposed by Burgess et al. [22] ensures the standardization of MR analyses and improves the quality and reproducibility of the results. Interpretation of MR results requires comprehensive evidence synthesis rather than reliance on isolated analyses. MRanalysis promotes a robust causal inference framework that integrates multiple analytical strategies, combining univariable MR for establishing basic causal relationships, multivariable MR for controlling confounding exposures, and mediation MR for elucidating causal pathways. Our platform facilitates robust validation through the integration of complementary analyt-

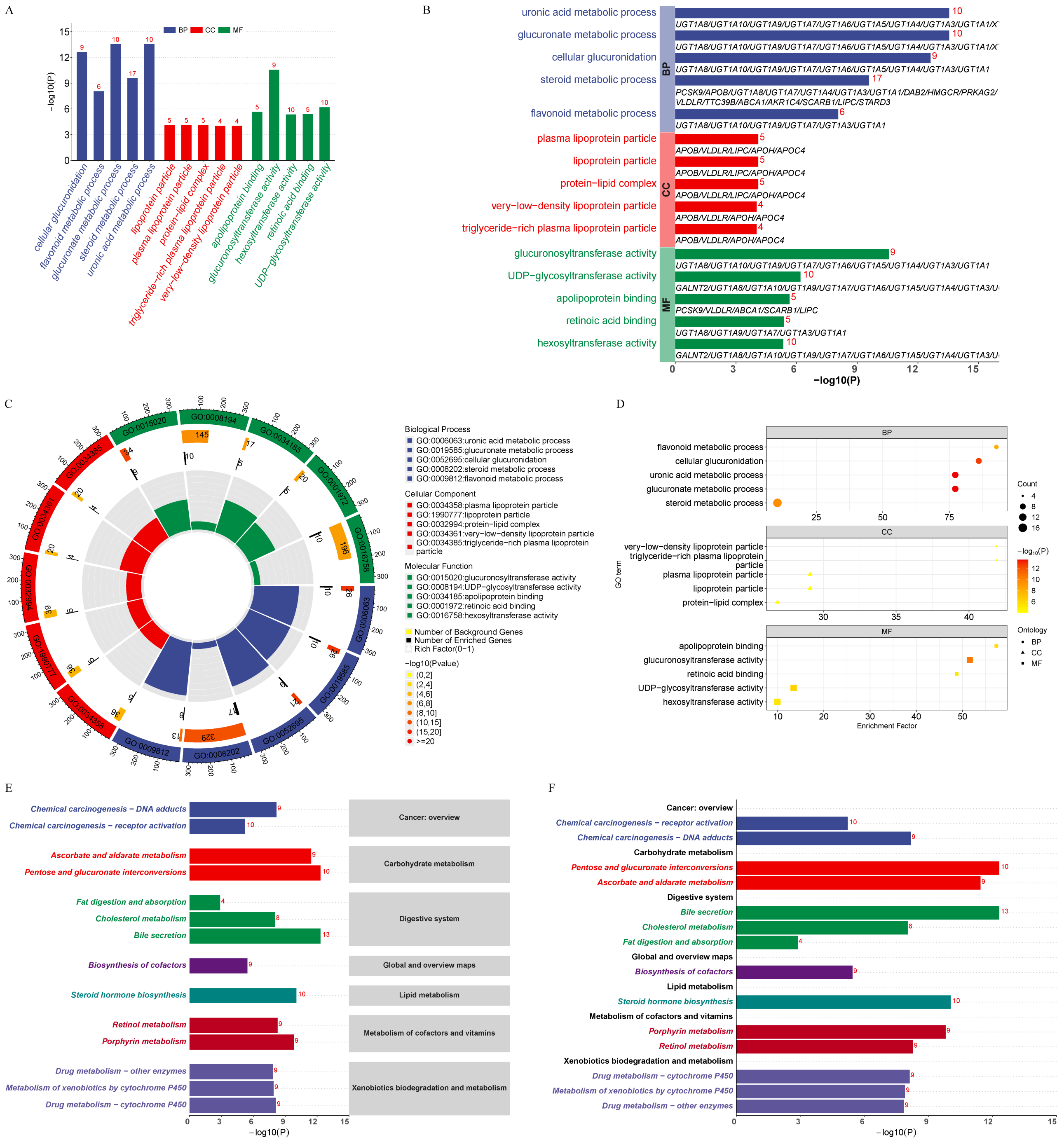

Figure 10. The results of GO/KEGG enrichment analyses. A. a vertical bar chart displaying GO enrichment; B. a horizontal bar chart displaying GO enrichment; C. a circos plot displaying GO enrichment; D. a dot plot displaying GO enrichment; E. a hierarchical horizontal bar chart displaying KEGG enrichment (Style I); F. a hierarchical horizontal bar chart displaying KEGG enrichment (Style II). GO, gene ontology; BP, biological process; CC, cellular component; MF, molecular function.

ical approaches, encompassing rigorous quality control procedures, colocalization analyses, fine-mapping studies, and pathway enrichment analyses where feasible. We emphasize that MR findings should be contextualized within broader evidence bases, including experimental validation, replication across diverse populations, and consistency with observational epidemiological studies. This framework treats MR results as one line of evidence for causality, not definitive proof, helping prevent overinterpretation that could mislead clinical decisions.

GWASkit, as a standalone tool and bridge, complements MRanalysis by facilitating the preprocessing of kinds of GWAS summary datasets. It is efficient handling of tasks such as rs ID mapping, format conversion, and data standardization enables seamless compatibility with a wide range of post-GWAS tools and applications within the MRanalysis platform. These case studies demonstrated the versatility, convenience and robustness of our platform. In particular, the case study on rs ID conversion shows that our GWASkit outperformed other existing tools, exhibiting high accuracy, fast processing speed, and moderate memory usage. In addition, the availability of GWASkit on multiple operating systems and its user-friendly interface makes it accessible to researchers with limited programming skills.

Currently, there are several online platforms for MR analysis (Table 4), with MR-Base [44] being one of the earliest, appearing in 2018. MR-Base is an analytical platform for MR developed by the MRC Integrative Epidemiology Unit (IEU) at the University

of Bristol. This team has also developed the OpenGWAS database [50], which is a database of genetic associations from GWAS summary dataset, available for online querying and download. Since the launch of the MR-Base and OpenGWAS database, other online tools have emerged, each with a slightly different focus. But most of these tools primarily support the two-sample MR approach and do not offer functionality for multivariable and mediation MR. Furthermore, these tools often lack the necessary steps for preprocessing and QC of GWAS datasets. In contrast to the existing platforms, MRanalysis is specially designed for MR analyses and related GWAS data processing. We aim to establish a comprehensive, standardized, and user-friendly open platform that caters to the needs of users conducting MR studies. By providing a complete workflow that includes data preprocessing, QC, and various MR approaches, our platform seeks to address the limitations of current platforms and facilitate more robust and reproducible MR analyses.

Another key strengths of MRanalysis lies in its transparency and reproducibility. By providing full access to the underlying code, we empower researchers to not only understand the intricacies of their analyses but also to modify and extend them as needed. This approach fosters a deeper engagement with the analytical process and promotes methodological rigor in MR studies. By integrating best practices and standardized workflows, we strive to ensure the reliability and consistency of MR analyses conducted using our platform. A crucial feature of MRanalysis is the real-time generation of complete code for all applications based on user-customized parameters. This allows users to gain a deeper understanding of the analytical steps involved. Simultaneously, those with extensive programming experience may prefer more flexible and customizable options. Leveraging this feature, users can generate code from the applications and then perform further personalized analyses locally. This capability bridges the gap between user-friendly interfaces and the need for advanced customization, catering to researchers with varying levels of technical expertise.

However, it's important to acknowledge some limitations and future directions for MR analysis and GWASkit. Several methodological limitations inherent to two-sample Mendelian randomization necessitate careful consideration during result interpretation. The fundamental assumption of shared genetic architecture and linkage disequilibrium patterns between exposure and outcome GWAS cohorts may be compromised when integrating studies across diverse ancestral populations or geographic regions. Differential population stratification between samples can introduce systematic bias, thereby undermining the validity of causal estimates. Furthermore, two-sample MR quantifies lifelong genetic effects, which may not accurately reflect the therapeutic potential of short-term interventions or late-onset exposures of clinical relevance. The methodology remains vulnerable to horizontal pleiotropy, wherein genetic instruments influence outcomes through biological pathways independent of the target exposure. Detecting such pleiotropic effects using summary-level statistics presents considerable analytical challenges. Statistical power is contingent upon instrument strength and GWAS sample dimensions, with weak instrument bias persisting despite apparently adequate F-statistics. Additional methodological concerns include collider bias, and the inability to model time-varying associations or non-linear dose-response relationships, all of which may confound causal inference. Thus, investigators should interpret two-sample MR findings as contributory evidence toward causal inference rather than definitive proof of causation, particularly when considering clinical translation. These methodological constraints underscore the critical importance of our platform's emphasis on comprehensive quality control procedures and multi-method validation for responsible causal inference in biomedical research. As the field of MR continues to evolve, the platform will require regular updates to incorporate new methods and address emerging challenges. Future developments may focus on expanding the platform's compatibility with a broader range of data formats and integrating more advanced visualization tech-

Table 4. Feature comparisons of currently available Mendelian randomization analysis platform.

Platform	Input	Data preprocessing	Free	QC	2SMR	MVMR	MMR	Colocalization Analysis	Visualization	Year	Cite
**MRanalysis**	**BI, UP**	**Yes**	**Yes**	**Yes**	**Yes**	**Yes**	**Yes**	**Yes**	**Yes**	**2025**[a]	-
MR-base	BI, UP[b]	No	Yes	No	Yes	No	No	No	No	2018	Hemani et al. [44]
MetaboAnalyst	BI[c]	No	Yes	No	Yes	No	No	No	Yes	2024	Pang et al. [45]
HiOmics	BI, UP	No	No	No	Yes	No	No	No	Yes	2023	Li et al. [46]
SUMMER	BI[d]	No	Yes	No	Yes	No	No	No	Yes	2019	Xin et al. [47]
MRbrowse	BI[e]	No	Yes	No	Yes	No	No	No	Yes	2018	-
ExPheWas	BI[f]	No	Yes	No	Yes	No	No	No	Yes	2021	Legault et al. [48]
MRAD	BI[g]	No	Yes	No	Yes	No	No	No	No	2024	Zhao et al. [49]

MR: Mendelian randomization; QC, quality control; 2SMR, two-sample MR analysis; MVMR: multivariable MR; MMR: Mediation MR; BI: built-in; UP: upload.
a. Last update time. b. Only the exposure data can be uploaded by users, while the outcome data is not supported for user uploads; c. the exposure data primarily focuses on metabolites; d. the outcome data primarily focuses on cancers; e. preperformed Mendelian Randomization (MR) analyses of 100s of proteins against 100s of clinical outcomes using genetic data; f. The platform reports on genetic associations between genes and phenotypes; g. The MRAD application was created to identify the risk of protective factors for Alzheimer's disease.

niques to facilitate result interpretation.

Conclusion

In summary, MRanalysis and GWASkit offer a comprehensive, efficient and user-centric solution for conducting MR analyses and handling GWAS summary data. By providing a unified and standardized platform that integrates diverse functionalities and promotes best practices, this platform has the potential to accelerate discoveries in genetic epidemiology, ultimately leading to improved understanding of complex diseases and more targeted interventions. As the user base of MRanalysis continues to grow, it is poised to become an essential resource for the genetic epidemiology community, empowering researchers to unravel causal relationships and advance our understanding of human health and disease.

Availability of source code and requirements

- Project name: MRanalysis/GWASkit
- Project home page: https://github.com/xingabao/MRanalysis; https://github.com/Li-OmicsLab-MPU/GWASkit
- Operating system(s): Windows, MacOS, Linux
- Programming language: R, Python, CSS, HTML, Dockerfile
- Other requirements: N/A
- License: The MRanalysis/GWASkit codebase is licensed with a CC0 1.0 license (dataset) and the MIT license.
- Bio.tools id: biotools:mranalysis (https://bio.tools/mranalysis)
- RRID:SCR_027338
- WorkflowHub: 10.48546/WORKFLOWHUB.WORKFLOW.1872.1

Abbreviations

API: application programming interface; BP: biological processes; CC: cellular components; CHD: coronary heart disease; CPU: central processing unit; DAG: Directed Acyclic Graph; FI: frailty index; GO: Gene Ontology; GWAS: genome-wide association study; HDL cholesterol: high-density lipoprotein cholesterol; IV: instrulmental variable; IVW: inverse variance weighted; KEGG: Kyoto Encyclopedia of Genes and Genomes; LD: linkage disequilibrium; LDL cholesterol: low-density lipoprotein cholesterol; LTS: long-term support ; MDD: major depression disorder; MF: molecular functions; MR: Mendelian randomization; MVMR: multivariable Mendelian randomization; OR: odds ratios; PGC: Psychiatric Genomics Consortium; QC: quality control; RCT: randomized controlled trail; SNP: single nucleotide polymorphism; UI: user interface; VCF: variant call format

Data Availability

Publicly available datasets were analyzed in this study. The GWAS summary statistics can be found in the MRC IEU OpenGWAS project, the EMBL-EBI GWAS Catalog, and the Edinburgh DataShare repository. The specific datasets used in this study are available under the following accession IDs: Major depression (MDD): Edinburgh DataShare (doi:10.7488/ds/2458) and OpenGWAS (ieu-b-102), Howard et al. [25]; frailty index: GWAS Catalog (GCST90020053) and OpenGWAS (ebi-a-GCST90020053), Atkins et al. [24]; HDL cholesterol: OpenGWAS (ieu-a-299), Gustafsson et al. [26]; LDL cholesterol: OpenGWAS (ieu-a-300), Gustafsson et al. [26]; Triglycerides: OpenGWAS (ieu-a-302), Gustafsson et al. [26]; Creatinine: OpenGWAS (met-d-Creatinine); Coronary heart disease: OpenGWAS (ieu-a-7), Nikpay et al. [27]; Feeling lonely: OpenGWAS (ebi-a-GCST006942), Nagel et al. [28]; Major depressive disorder in trauma-unexposed individuals (MDD): OpenGWAS (ebi-a-GCST009981), Coleman et al. [29]; Cigarettes smoked per day: OpenGWAS (ieu-b-142), Liu et al. [30]. The database search was completed on August 15, 2024.

Acknowledgements

We gratefully acknowledge the invaluable contributions of all data generators and consortiums whose publicly available GWAS summary statistics made this work possible, including but not limited to the Psychiatric Genomics Consortium, UK Biobank, EMBL-EBI GWAS Catalog, and the MRC IEU OpenGWAS project. We also thank the developers and maintainers of key open-source resources and analytical tools utilized in this study, including dbSNP (NCBI), TwoSampleMR, MVMR, MAGMA, CheckSumStats, clusterProfiler, and other essential R and Python packages.

We sincerely appreciate the broader R, Python, and bioinformatics communities, whose continued efforts have provided the foundations for the development of MRanalysis and GWASkit.

Author Contributions

Abao Xing: Investigation, Conceptualization, Formal analysis, Methodology, Validation, Writing—original draft & editing. Tiantian Cai: Investigation, Data curation, Formal analysis, Visualization, Writing—review & editing. Haofan Du Investigation, Formal analysis, Visualization, Writing—review & editing. Zhifan Li: Methodology, Visualization. HoiMan Ng: Supervision, Validation. Junrong Li: Methodology, Formal analysis. Guanmin Jiang: Supervision, Validation. Lijun Chen: Conceptualization, Supervision, Methodology, Validation. Kefeng Li: Supervision, Project administration, Funding acquisition, Writing – review & editing.

Funding

This work was supported by the fund from Macao Polytechnic University (RP/FCA-14/2023), The Science and Technology Development Funds(FDCT) of Macao (0033/2023/RIB2), and the Joint Research Funding Program between FDCT and the Department of Science and Technology of Guangdong Province (FDCT-GDST) (0009/2024/AGJ) with the submission approval IDfca.f852.focf,2].

Competing Interests

The authors declare that they have no competing interests.

Ethics Statement

All test data in this study were derived from public GWAS summary-level data, ethics approval was not required for the present study.

References

1. Hu X, Zhao J, Lin Z, Wang Y, Peng H, Zhao H, et al. Mendelian randomization for causal inference accounting for pleiotropy and sample structure using genome-wide summary statistics. Proceedings of the National Academy of Sciences 2022;119(28):e2106858119. https://doi.org/10.1073/pnas.2106858119.
2. Boehm FJ, Zhou X. Statistical methods for Mendelian randomization in genome-wide association studies: a review. Computational and structural biotechnology journal 2022;20:2338–2351. https://doi.org/10.1016/j.csbj.2022.05.015.
3. Ference BA, Holmes MV, Smith GD. Using Mendelian randomization to improve the design of randomized trials. Cold

Spring Harbor perspectives in medicine 2021;11(7):a040980. https://doi.org/10.1101/cshperspect.a040980.
4. Hernán MA, Wang W, Leaf DE. Target trial emulation: a framework for causal inference from observational data. JAMA 2022;328(24):2446–2447. https://doi.org/10.1001/jama.2022.21383.
5. Broglio K. Randomization in clinical trials: permuted blocks and stratification. JAMA 2018;319(21):2223–2224. https://doi.org/10.1001/jama.2018.6360.
6. Yu H, Wan X, Yang M, Xie J, Xu K, Wang J, et al. A large-scale causal analysis of gut microbiota and delirium: a Mendelian randomization study. Journal of affective disorders 2023;329:64–71. https://doi.org/10.1016/j.jad.2023.02.078.
7. Li J, Tang M, Gao X, Tian S, Liu W. Mendelian randomization analyses explore the relationship between cathepsins and lung cancer. Communications biology 2023;6(1):1019. https://doi.org/10.1038/s42003-023-05408-7.
8. Ye CJ, Liu D, Chen ML, Kong LJ, Dou C, Wang YY, et al. Mendelian randomization evidence for the causal effect of mental well-being on healthy aging. Nature Human Behaviour 2024;8(9):1798–1809. https://doi.org/10.1038/s41562-024-01905-9.
9. Pozarickij A, Gan W, Lin K, Clarke R, Fairhurst-Hunter Z, Koido M, et al. Causal relevance of different blood pressure traits on risk of cardiovascular diseases: GWAS and Mendelian randomisation in 100,000 Chinese adults. Nature Communications 2024;15(1):6265. https://doi.org/10.1038/s41467-024-50297-x.
10. Cronjé HT, Karhunen V, Hovingh GK, Coppieters K, Lagerstedt JO, Nyberg M, et al. Genetic evidence implicating natriuretic peptide receptor-3 in cardiovascular disease risk: a Mendelian randomization study. BMC medicine 2023;21(1):158. https://doi.org/10.1186/s12916-023-02867-x.
11. Larsson SC, Butterworth AS, Burgess S. Mendelian randomization for cardiovascular diseases: principles and applications. European heart journal 2023;44(47):4913–4924. https://doi.org/10.1093/eurheartj/ehad736.
12. Levin MG, Burgess S. Mendelian randomization as a tool for cardiovascular research: a review. JAMA cardiology 2024;9(1):79–89. https://doi.org/10.1001/jamacardio.2023.4115.
13. Wang Z, Chen J, Zhu L, Jiao S, Chen Y, Sun Y. Metabolic disorders and risk of cardiovascular diseases: a two-sample mendelian randomization study. BMC Cardiovascular Disorders 2023;23(1):529. https://doi.org/10.1186/s12872-023-03567-3.
14. Du Zx, Ren Yy, Wang Jl, Li Sx, Hu Yf, Wang L, et al. The potential association between metabolic disorders and pulmonary tuberculosis: a Mendelian randomization study. European Journal of Medical Research 2024;29(1):277. https://doi.org/10.1186/s40001-024-01845-0.
15. Mu C, Dang X, Luo XJ. Mendelian randomization analyses reveal causal relationships between brain functional networks and risk of psychiatric disorders. Nature human behaviour 2024;8(7):1417–1428. https://doi.org/10.1038/s41562-024-01879-8.
16. Gao X, Qin Y, Jiao S, Hao J, Zhao J, Wang J, et al. Genetic evidence for the causal relations between metabolic syndrome and psychiatric disorders: a Mendelian randomization study. Translational Psychiatry 2024;14(1):46. https://doi.org/10.1038/s41398-024-02759-5.
17. Yu Y, Hou L, Wu Y, Yu Y, Liu X, Wu S, et al. Causal associations between female reproductive behaviors and psychiatric disorders: a lifecourse Mendelian randomization study. BMC psychiatry 2023;23(1):799. https://doi.org/10.1186/s12888-023-05203-y.
18. Wang W, Li W, Zhang D, Mi Y, Zhang J, He G. The causal relationship between PCSK9 inhibitors and malignant tumors: a mendelian randomization study based on drug targeting. Genes 2024;15(1):132. https://doi.org/10.3390/genes15010132.
19. Burgess S, Mason AM, Grant AJ, Slob EA, Gkatzionis A, Zuber V, et al. Using genetic association data to guide drug discovery and development: review of methods and applications. The American Journal of Human Genetics 2023;110(2):195–214. https://doi.org/10.1016/j.ajhg.2022.12.017.
20. Duan QQ, Wang H, Su WM, Gu XJ, Shen XF, Jiang Z, et al. TBK1, a prioritized drug repurposing target for amyotrophic lateral sclerosis: evidence from druggable genome Mendelian randomization and pharmacological verification in vitro. BMC medicine 2024;22(1):96. https://doi.org/10.1186/s12916-024-03314-1.
21. Wootton RE, Sallis HM. Let's call it the effect allele: a suggestion for GWAS naming conventions. International journal of epidemiology 2020;49(5):1734–1735. https://doi.org/10.1093/ije/dyaa149.
22. Burgess S, Smith GD, Davies NM, Dudbridge F, Gill D, Glymour MM, et al. Guidelines for performing Mendelian randomization investigations: update for summer 2023. Wellcome open research 2023;4:186. https://doi.org/10.12688/wellcomeopenres.15555.3.
23. Wray NR, Ripke S, Mattheisen M, Trzaskowski M, Byrne EM, Abdellaoui A, et al. Genome-wide association analyses identify 44 risk variants and refine the genetic architecture of major depression. Nature genetics 2018;50(5):668–681. https://doi.org/10.1038/s41588-018-0090-3.
24. Atkins JL, Jylhävä J, Pedersen NL, Magnusson PK, Lu Y, Wang Y, et al. A genome-wide association study of the frailty index highlights brain pathways in ageing. Aging cell 2021;20(9):e13459. https://doi.org/10.1111/acel.13459.
25. Howard DM, Adams MJ, Clarke TK, Hafferty JD, Gibson J, Shirali M, et al. Genome-wide meta-analysis of depression identifies 102 independent variants and highlights the importance of the prefrontal brain regions. Nature neuroscience 2019;22(3):343–352. https://doi.org/10.1038/s41593-018-0326-7.
26. Gustafsson SK, Ganna A, et al. Discovery and refinement of loci associated with lipid levels. Nature Genetics 2013;45(11):1274–1283. https://doi.org/10.1038/ng.2797.
27. Nikpay M, Goel A, Won H, Hall L, Willenborg C, Kanoni S, et al. A comprehensive 1000 Genomes–based genome-wide association meta-analysis of coronary artery disease. Nature genetics 2015;47(10):1121–1130. https://doi.org/10.1038/ng.3396.
28. Nagel M, Watanabe K, Stringer S, Posthuma D, Van Der Sluis S. Item-level analyses reveal genetic heterogeneity in neuroticism. Nature communications 2018;9(1):905. https://doi.org/10.1038/s41467-018-03242-8.
29. Coleman JR, Peyrot WJ, Purves KL, Davis KA, Rayner C, Choi SW, et al. Genome-wide gene-environment analyses of major depressive disorder and reported lifetime traumatic experiences in UK Biobank. Molecular psychiatry 2020;25(7):1430–1446. https://doi.org/10.1038/s41380-019-0546-6.
30. Liu M, Jiang Y, Wedow R, Li Y, Brazel DM, Chen F, et al. Association studies of up to 1.2 million individuals yield new insights into the genetic etiology of tobacco and alcohol use. Nature genetics 2019;51(2):237–244. https://doi.org/10.1038/s41588-018-0307-5.
31. Surapaneni A, Schlosser P, Zhou L, Liu C, Chatterjee N, Arking DE, et al. Identification of 969 protein quantitative trait loci in an African American population with kidney disease attributed to hypertension. Kidney international 2022;102(5):1167–1177. https://doi.org/10.1016/j.kint.2022.07.005.
32. Haycock PC, Borges MC, Burrows K, Lemaitre RN, Harrison S, Burgess S, et al. Design and quality control of large-scale two-sample Mendelian randomization studies. International journal of epidemiology 2023;52(5):1498–1521. https://doi.org/10.1093/ije/dyad018.

33. Wang Z, Dou Y, Chen L, Feng W, Zou Y, Xiao J, et al. Mendelian randomization identifies causal effects of major depressive disorder on accelerated aging. Journal of Affective Disorders 2024;358:422–431. https://doi.org/10.1016/j.jad.2024.05.056.
34. De Leeuw CA, Mooij JM, Heskes T, Posthuma D. MAGMA: generalized gene-set analysis of GWAS data. PLoS computational biology 2015;11(4):e1004219. https://doi.org/10.1371/journal.pcbi.1004219.
35. Xu S, Hu E, Cai Y, Xie Z, Luo X, Zhan L, et al. Using clusterProfiler to characterize multiomics data. Nature protocols 2024;19(11):3292–3320. https://doi.org/10.1038/s41596-024-01020-z.
36. Burgess S. Sample size and power calculations in Mendelian randomization with a single instrumental variable and a binary outcome. International journal of epidemiology 2014;43(3):922–929. https://doi.org/10.1093/ije/dyu005.
37. Brion MJA, Shakhbazov K, Visscher PM. Calculating statistical power in Mendelian randomization studies. International journal of epidemiology 2013;42(5):1497–1501. https://doi.org/10.1093/ije/dyt179.
38. Davies NM, Holmes MV, Smith GD. Reading Mendelian randomisation studies: a guide, glossary, and checklist for clinicians. bmj 2018;362. https://doi.org/10.1136/bmj.k601.
39. Wang K, Li M, Hakonarson H. ANNOVAR: functional annotation of genetic variants from high-throughput sequencing data. Nucleic acids research 2010;38(16):e164–e164. https://doi.org/10.1093/nar/gkq603.
40. Cingolani P, Platts A, Wang LL, Coon M, Nguyen T, Wang L, et al. A program for annotating and predicting the effects of single nucleotide polymorphisms, SnpEff: SNPs in the genome of Drosophila melanogaster strain w1118; iso-2; iso-3. fly 2012;6(2):80–92. https://doi.org/10.4161/fly.19695.
41. Murphy AE, Schilder BM, Skene NG. MungeSumstats: a Bioconductor package for the standardization and quality control of many GWAS summary statistics. Bioinformatics 2021;37(23):4593–4596. https://doi.org/10.1093/bioinformatics/btab665.
42. He Y, Koido M, Shimmori Y, Kamatani Y. GWASLab: a Python package for processing and visualizing GWAS summary statistics. Jxiv 2023;https://doi.org/10.51094/jxiv.370.
43. Oscanoa J, Sivapalan L, Gadaleta E, Dayem Ullah AZ, Lemoine NR, Chelala C. SNPnexus: a web server for functional annotation of human genome sequence variation (2020 update). Nucleic acids research 2020;48(W1):W185–W192. https://doi.org/10.1093/nar/gkaa420.
44. Hemani G, Zheng J, Elsworth B, Wade KH, Haberland V, Baird D, et al. The MR-Base platform supports systematic causal inference across the human phenome. elife 2018;7:e34408. https://doi.org/10.7554/eLife.34408.
45. Pang Z, Lu Y, Zhou G, Hui F, Xu L, Viau C, et al. MetaboAnalyst 6.0: towards a unified platform for metabolomics data processing, analysis and interpretation. Nucleic acids research 2024;52(W1):W398–W406. https://doi.org/10.1093/nar/gkae253.
46. Li W, Zhang Z, Xie B, He Y, He K, Qiu H, et al. HiOmics: A cloud-based one-stop platform for the comprehensive analysis of large-scale omics data. Computational and Structural Biotechnology Journal 2024;23:659–668. https://doi.org/10.1016/j.csbj.2024.01.002.
47. Xin J, Gu D, Chen S, Ben S, Li H, Zhang Z, et al. SUMMER: a Mendelian randomization interactive server to systematically evaluate the causal effects of risk factors and circulating biomarkers on pan-cancer survival. Nucleic acids research 2023;51(D1):D1160–D1167. https://doi.org/10.1093/nar/gkac677.
48. Legault MA, Perreault LPL, Dubé MP. ExPheWas: a browser for gene-based pheWAS associations. Nucleic acids research 2022;50(W1):W305––W31. https://doi.org/10.1093/nar/gkac289.
49. Zhao T, Li H, Zhang M, Xu Y, Zhang M, Chen L. Systematic evaluation of multifactorial causal associations for Alzheimer's disease and an interactive platform MRAD developed based on Mendelian randomization analysis. Elife 2024;13:RP96224. https://doi.org/10.7554/eLife.96224.
50. Elsworth B, Lyon M, Alexander T, Liu Y, Matthews P, Hallett J, et al. The MRC IEU OpenGWAS data infrastructure. BioRxiv 2020;p. 2020–08. https://doi.org/10.1101/2020.08.10.244293.

Click here to access/download
Supplementary Material
Rebuttal letter_20250816.docx

Cover letter

Click here to access/download;Personal Cover;Cover letter_20250817.docx

澳門理工大學
Universidade Politécnica de Macau
Macao Polytechnic University

GigaScience Aug 18, 2025

Re: Major revision

Dear Editor,

Thank you for your letter and for coordinating the review of our manuscript, "**MRanalysis: A Comprehensive Online Platform for Integrated, Multi-Method Mendelian Randomization and Associated Post-GWAS Analyses**" (GIGA-D-25-00094). We are grateful to the reviewers for their constructive feedback. We have revised the platform and manuscript accordingly and are submitting a comprehensive point-by-point response with the revised files.

Below we summarize the key revisions and how they address the reviewers' main concerns:

1. Licensing, achknowledgements, and transparency

- We audited all integrated packages and tools and confirmed that they are released under permissive open-source licenses (primarily Apache License 2.0, MIT and Unlicense). We added explicit attributions and citations in both the manuscript and the platform website.

- The full MRanalysis codebase is open-sourced for transparency and community contribution:

GitHub: https://github.com/xingabao/MRanalysis

Docker Hub: https://hub.docker.com/r/xingabao/mranalysis

2. Novel contributions and functionality beyond standard MR

- High-performance GWAS format conversion and fast VCF parsing. We released a dedicated FORMAT application enabling flexible VCF/TSV/CSV conversion and column mapping, with substantial speed and memory improvements for large GWAS summary files (typical VCF parsing in 2 ~ 3 miniutes).

FORMAT app: https://mranalysis.cn/plugins/FORMAT/

- Integrated colocalization analysis

We added four widely used methods -- COLOC.ABF, COLOC.SUSIE, COLOC.PWCoCo, and COLOC. eCAVIAR – with unified workflows, visualization, and reporting.

COLOC app: https://mranalysis.cn/plugins/COLOC/

- Enhanced SNP-to-gene mapping and enrichment with translational integration. Gene-level outputs now include gene symbols, full names, and functional annotations, plus direct links to Open Targets, DrugBank, and Clinical.Trials.gov to facilitate target triage and evidence review.

MAGMA app: https://mranalysis.cn/plugins/MAGMA/

3. Scalability and performance

- We provide a ready-to-run Docker image enabling local development within 2 to 3 minutes, eliminating version conflits and supporting large-scale GWAS processing on user's own infrastructure.

- We optimized I/O and computation (including 2SMR, MMR and MVMR) to reduce end-to-end runtimes (commonly ~10× faster compared with previous VariantAnnotation/gwasglue-based workflows).

4. Mendiation MR: justification and limitations.

- We clarified the exposure-mediator-outcome rationale in Case VI (loneliness → cigarettes/day → MDD) and emphasized that summary-data mediation MR follows the product-of-coefficients approach (Burgess et al., 2015) with explicit assumptions and limitations.

- We cautioned that mediation MR using summary statistics is methodological demonstration and should be interpreted alongside complementary evidence.

5. Power and sample size.

We added Case VIII to demonstrate power and minimum sample size estimation across univariable, multivariable, and mediation MR (80% power, two-sided α=0.05), using the integrated calculators and standard methodology (Burgess, 2014). Results are reported in the manuscript and available in the platform.

6. Guidance to avoid misue and over-interpretation.

We expanded the DISCUSSION to: a) Encourage biologically grounded hypotheses and best-practice design. b) Promote triangulation across univariable, multivariable, and mediation MR, and integration with complementary evidence (fine-mapping, colocalization, and analyses of individual-level data where available). c) Highlight the limitations of two-sample MR, including sample overlap, heterogeneity, instrument strength, horizontal pleiotropy, potential collider bias, and the challenges of time-varing and non-linear effects.

7. Website security and access.

We upgraded our SSL certificate and conducted repeated security checks. For institutions with strict network policies, the Docker-based deployment provides a secure, offline alternative.

Resource registration and access:

We have registered MRanalysis and its workflows in bio.tools and SciCrunch to obtain biotoolsID (biotools:mranalysis) and RRID (SCR_027338, pending approval), respectively, and also have applied a WorkflowHub DOI (10.48546/WORKFLOWHUB.WORKFLOW.1872.1). We have included these identifiers and links in the revised manuscript.

Availability of source code and requirements

- Project name: MRanalysis/GWASkit
- Project home page: https://github.com/xingabao/MRanalysis; https://github.com/Li-OmicsLab-MPU/GWASkit
- Operating system(s): Windows, MacOS, Linux
- Programming language: R, Python, CSS, HTML, Dockerfile
- Other requirements: N/A
- License: The MRanalysis/GWASkit codebase is licensed with a CC0 1.0 license (dataset) and the MIT license.
- Bio.tools id: biotools:mranalysis (https://bio.tools/mranalysis)
- RRID:SCR_027338
- WorkflowHub: 10.48546/WORKFLOWHUB.WORKFLOW.1872.1

We sincerely appreciate the reviwers's highlights, which have materially strengthened the platform's methodological completeness, performance, and translational value. We hope the revised manuscript will be suitable for further consideration at GigaScience.

Thank you very much for your time on our manuscript.

Sincerely

Kefeng Li

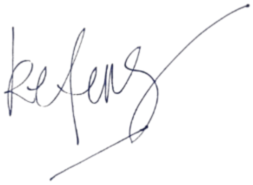

Centre for Artificial Intelligence Driven Drugs Discovery,

Faculty of Applied Sciences,

Macao Polytechnic University

Macau

E-mail: kefengl@mpu.edu.mo

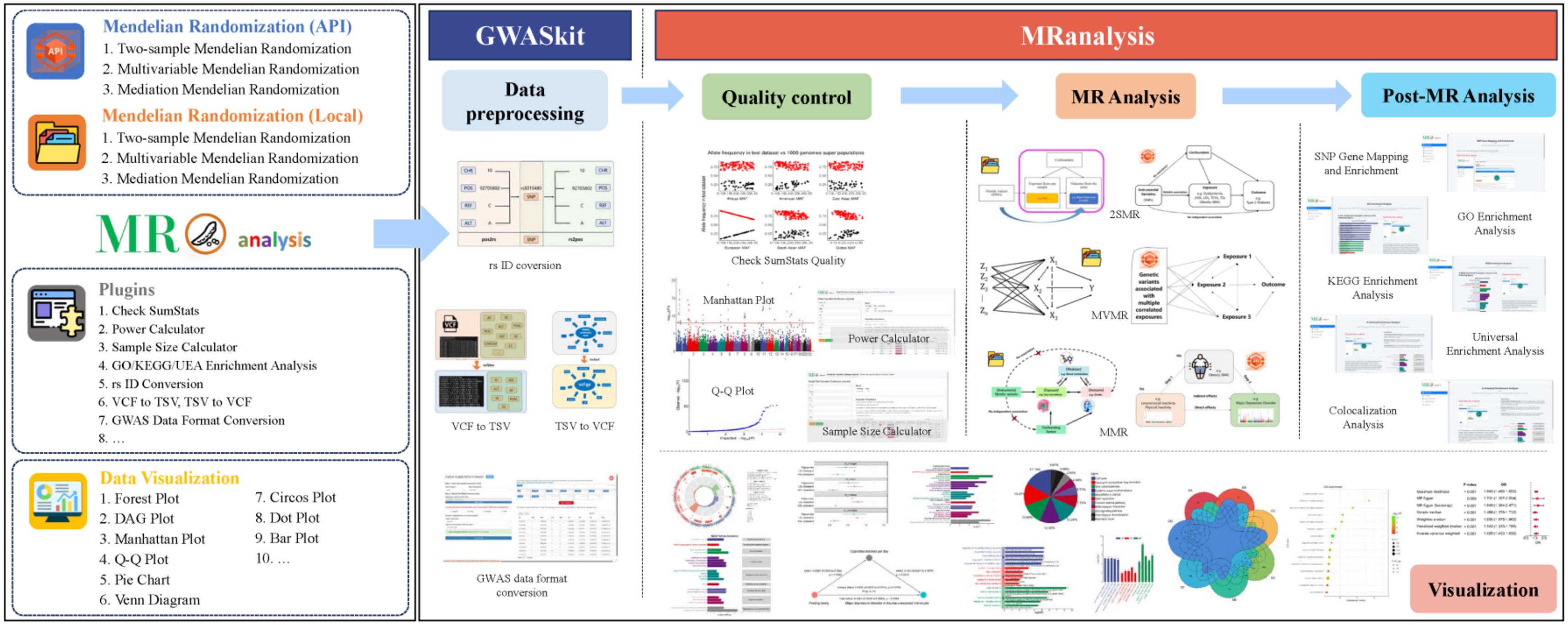
Mendelian Randomization (API)
1. Two-sample Mendelian Randomization
2. Multivariable Mendelian Randomization
3. Mediation Mendelian Randomization
Mendelian Randomization (Local)
1. Two-sample Mendelian Randomization
2. Multivariable Mendelian Randomization
3. Mediation Mendelian Randomization
MR analysis
Plugins
1. Check SumStats
2. Power Calculator
3. Sample Size Calculator
4. GO/KEGG/UEA Enrichment Analysis
5. rs ID Conversion
6. VCF to TSV, TSV to VCF
7. GWAS Data Format Conversion
8. …
Data Visualization
1. Forest Plot
2. DAG Plot
3. Manhattan Plot
4. Q-Q Plot
5. Pie Chart
6. Venn Diagram
7. Circos Plot
8. Dot Plot
9. Bar Plot
10. …
GWASkit
MRanalysis
Data preprocessing
Quality control
MR Analysis
Post-MR Analysis
rs ID coversion
VCF to TSV
TSV to VCF
GWAS data format conversion
Check SumStats Quality
Manhattan Plot
Power Calculator
Q-Q Plot
Sample Size Calculator
2SMR
MVMR
MMR
SNP Gene Mapping and Enrichment
GO Enrichment Analysis
KEGG Enrichment Analysis
Universal Enrichment Analysis
Colocalization Analysis
Visualization
