# Supplementary material for: MRanalysis: a comprehensive online platform for integrated, multimethod Mendelian randomization and associated post-GWAS analyses
Source: Gigascience. 2025 Oct 22;14:giaf131. doi: 10.1093/gigascience/giaf131 (PMC12616851; doi:10.1093/gigascience/giaf131)
Supplement: giaf131_Authors_Response_To_Reviewer_Comments_Original_Submission [file giaf131_authors_response_to_reviewer_comments_original_submission.pdf]

Author's Response To Reviewer Comments

Responses to the editor's comments (Please also see the attachment in the submission portal as a supplemental file)

We provided the "point-to-point" responses to the editor questions below (Please also see the attachment in the submission portal as a supplemental file):

Reviewer #1

In this study, Xing et al. presented an online platform for performing GWAS data preprocessing, Mendelian Randomization (MR) analyses, and post-GWAS annotation. Multiple standard tools have been implemented to ease MR steps and facilitate more robust genetic analyses to infer complex relationships between complex traits. While I see the value of developing integrated platform for MR users, I also have several major concerns regarding to its novelty, framework, and interpretation.

1. While GWASkit is a self-developed tool, all those 3 types of MR analyses and their R packages were previously developed. The novelty level for method part is not high. I am a little concerned about the copyright for implementing those existing tools and suggest adding some acknowledgement or evidence of agreement from original developers.

Response: We sincerely thank you for your thoughtful and constructive comments on our manuscript. We greatly appreciate your recognition of the potential value of our integrated platform for Mendelian randomization (MR) users, as well as your detailed feedback regarding its novelty, framework, and interpretation. Your insights are invaluable in helping us improve the quality and clarity of our work.
In response to your comments, we have taken proactive steps to ensure full compliance with copyright regulations and to acknowledge the contributions of original developers. Your feedback has been instrumental in guiding further improvements to our study. Please find our detailed response below:

(1) Regarding the copyright:

We have thoroughly reviewed the open-source licenses of the key R packages and tools utilized in our project. All of them are distributed under either the Unlicense, the MIT License, or the Apache License 2.0. Each of these licenses explicitly permits free use, reproduction, modification, and incorporation into other projects, especially for academic and non-commercial purposes. To further demonstrate our respect for the original developers, we have clearly acknowledged the use of these packages on our project homepage, cited them appropriately within the manuscript, and expressed our gratitude to the original authors.

(2) Enhancement and innovation in our tool beyond MR analysis:

To improve the functionality and usability of MRanalysis, we have introduced the following new features:
a). To address the challenge of GWAS data format heterogeneity and to improve analysis efficiency, we have developed an application that enables rapid and flexible conversion between mainstream GWAS data formats, including VCF, TSV, and CSV. Notably, our approach introduces an innovative method for parsing and processing VCF files. Traditionally, tools such as VariantAnnotation and gwasglue require more than ten minutes and substantial memory resources to load and parse large-scale GWAS files. In contrast, our optimized implementation achieves VCF parsing in approximately two to three minutes, with significantly reduced memory usage.

The address of this application: https://mranalysis.cn/plugins/FORMAT/

This improvement is achieved through a series of algorithmic and data structure optimizations tailored for GWAS summary data, allowing for seamless data integration and compatibility with downstream Mendelian randomization analyses. By providing a fast and user-friendly tool for GWAS format standardization, we

facilitate reproducible and efficient MR workflows for users with varying technical backgrounds.

b). New interactive applications for colocalization analyses within MRanalysis, including support for COLOC.ABF, COLOC.SUSIE, and COLOC.eCAVIAR, expanding the scope of MRanalysis.
The address of this application: https://mranalysis.cn/plugins/COLOC/

This enhancement broadens the utility of MRanalysis, allowing users to seamlessly conduct comprehensive colocalization analyses alongside MR studies, and facilitating more reliable exploration of shared genetic signals between traits.

c). Upgraded SNP gene mapping and enrichment with comprehensive annotation and translational integration.
The address of this application: https://mranalysis.cn/plugins/MAGMA/

We have significantly enhanced the SNP gene mapping and enrichment application in MRanalysis. During SNP-to-gene annotation, the upgraded application now provides detailed gene information, including gene symbols, full gene names, and functional annotations are automatically displayed for each mapped gene, facilitating immediate biological interpretation.
To further support translational research, we have addressed the previous lack of integration with drug target and clinical trial resources. This application now incorporates direct links and annotation retrieval from widely used resources such as Open Targets, DrugBank, and ClinicalTrials.gov. For each gene, users can instantly access associated drug target data and ongoing or completed clinical trials via embedded hyperlinks to the relevant of official database entries. The seamless integration enables users to trace MR findings to known or emerging drug targets, evaluate pharmacological or clinical evidence, and more efficiently prioritize genes for follow-up functional or therapeutic investigation.

These enhancements collectively empower users to bridge genetic findings with actionable biological and clinical insights, greatly expanding the scope and translational utility of MRanalysis.

d). Open-source release and deployment optimization

GitHub: https://github.com/xingabao/MRanalysis
Docker Hub: https://hub.docker.com/r/xingabao/mranalysis

To promote transparency, reproducibility, and community engagement, we have fully open-sourced the MRanalysis codebase on GitHub. This allows users to inspect the complete workflow, report issues, and contribute to further development. The platform currently consists of over 60,000 lines of code, not only integrating numerous established packages and tools, but also implementing a wide range of self-developed features – including rs ID mapping, universal GWAS format conversion, fast VCF file parsing, and versatile data visualization applications. This comprehensive open-source release enables users to inspect the entire workflow, report issues, and contribute to further development.

Given the scale and complexity of the codebase, conventional installation can be time-consuming (often requiring 3 to 4 hours) and susceptible to software version inconsistencies. To address this, we provide an official Docker image for MRanalysis. With Docker, users can deploy the complete environment locally within 2 to 3 minutes, ensuring reproducibility and greatly lowering the barrier for ordinary users.
In addition, the Docker-based local deployment enables efficient processing of large-scale GWAS summary files and accelerates computationally intensive modules such as GWAS data quality control, MR analysis and GWAS format conversion, effectively overcoming previous limitations on data size and analysis speed.

In summary, we have ensured full compliance with copyright regulations, introduced multiple practical and innovative features, and provided open-source access with one-click deployment to maximize usability. Furthermore, we have duly acknowledged the developers of the original tools or packages in our project documentation and manuscript. We hope these efforts will enhance the scientific value and community impact of MRanalysis.

2. About mediation MR: the justification of defining exposure, mediator, and outcome is not clear. Without individual-level data, how valid is the estimated mediation proportion using sum data? In other words, does lonely-cigarettes-MDD result in case VI align well with traditional one-sample mediation result?

Response: Thank you for your thoughtful comments regarding the mediation MR analysis.

In case VI, we defined "feeling lonely" as the exposure, "cigarettes smoked per day" as the mediator, and "MDD" as the outcome. This pathway is justified based on established epidemiological and biological evidence: loneliness is a recognized psychosocial risk factor for mental health, and smoking is often used to cope with loneliness and stress, which in turn can influence depression risk.

Regarding the use of mediation MR with summary-level data, we fully acknowledge its limitations. Unlike traditional mediation analysis that requires individual-level data, mediation MR based on summary statistics typically adopts the product-of-coefficients method ($\beta1\times \beta2$) to estimate mediation effects. The method was originally proposed and developed by Burgess et al. (2015) [1] and has since been applied in several peer-reviewed studies [2,3]. However, we emphasize that this approach relies on the core MR assumptions and is subject to additional constraints inherent to the use of summary data. Direct comparison with traditional one-sample mediation analysis is not possible here due to the lack of large individual-level datasets that include all relevant variables.

Our implementation in MRanalysis is intended primarily as a methodological demonstration. We have clearly referenced the original methodology and cited relevant literature in our documentation. Users are encouraged to consider these methodological limitations when interpreting results and to consult the cited studies for further details and appropriate use cases. Ultimately, the choice to apply summary-based mediation MR should be made with caution and in the context of specific research questions.

1. Burgess, Stephen, et al. "Network Mendelian randomization: using genetic variants as instrumental variables to investigate mediation in causal pathways." International journal of epidemiology 44.2 (2015): 484-495.
2. Carter, Alice R., et al. "Mendelian randomisation for mediation analysis: current methods and challenges for implementation." European journal of epidemiology 36.5 (2021): 465-478.
3. Sanderson, Eleanor, et al. "Mendelian randomization." Nature reviews Methods primers 2.1 (2022): 6.

3. About post-MR: besides enrichment and visualization, other post-GWAS analysis (e.g., colocalization) that is supplementary to MR should be incorporated, as this will be essential for drug-target selection.

Response: Thank you very much for your valuable suggestion regarding the incorporation of additional post-GWAS analyses, such as colocalization, to supplement Mendelian randomization (MR) and enhance drug-target selection.

In response to your feedback, we have now integrated four widely used colocalization methods into our platform: 1) COLOC.ABF: Bayesian framework estimating the probability that two traits share a causal variant. 2) COLOC.SUSIE: Utilizes the Sum of Single Effects (SuSiE) model for complex multi-SNP colocalization analysis. 3) COLOC.PWCoCo: Hierarchical clustering-based approach to identify shared causal variants across traits. 4) COLOC.eCAVIAR: CAusal Variants Identification in Associated Regions for rigorous colocalization assessment.
These tools allow users to assess whether the genetic association signals for exposure and outcome are likely due to the same underlying causal variant, thereby strengthening the causal inference from MR and facilitating more robust drug-target prioritization.

We have updated the manuscript and user documentation to reflect this important addition. We appreciate your insightful suggestion, which has helped to significantly improve the scientific utility and translational relevance of our platform.

The address of this application: https://mranalysis.cn/plugins/COLOC/

4. Power and sample size calculation: I would suggest adding another case example for showing sample size needed for 3 types of MR analysis.

Response: Thank you for your constructive suggestion to include a case example demonstrating the minimum sample size requirements for the three types of Mendelian randomization (MR) analyses. In response, we have added Case VIII to both the manuscript (Table 3) and the MRanalysis platform.

We can utilize the integrated PowerCalculator and SampleSizeCalculator applications within the platform to estimate the statistical power and minimum required sample size for each MR analysis type. For every exposure–outcome pair in Case VIII, we reported the minimum sample size necessary to achieve 80% power at a two-sided significance level of 0.05, using the SampleSizeCalculator application and the methodology described by Burgess (2014).

The key parameters considered included the proportion of variance in the exposure explained by genetic instruments (R2), the anticipated causal effect size (odds ratio per standard deviation for binary outcomes or standard deviation change per standard deviation for continuous outcomes), the significance level (α), and, for binary outcomes, the case-control ratio. For two-sample MR analyses, the sample size was defined as the number of participants in the variant–outcome association dataset.

We believe these additions substantially enhance the comprehensiveness and practical utility of our resource. Thank you again for your valuable feedback.

5. About Discussion:
1) though it is important to ease MR analysis for all researchers, there is also a caution to avoid abusing MR for randomly selected cross-trait analysis. Need to discuss this when designing MR analysis.
Response: Thank you for your constructive comment regarding the potential risk of misusing Mendelian randomization (MR) for arbitrarily selected cross-trait analyses. We fully agree that, while improving accessibility to MR is important, careful consideration must be given to study design in order to avoid spurious or biologically implausible results.
In response, we have revised the Discussion section to explicitly address this point, emphasizing the need for clear biological rationale, robust hypothesis generation, and best-practice guidelines when selecting exposure-outcome pairs for MR analysis. We believe this addition will help users better understand the importance of proper study design and interpretation when using MRanalysis.
Furthermore, we have included prominent reminders on our website regarding the methodological rigor, evidence integration, and known limitations of MR, to further guide users in conducting reliable and valid analyses.

For your convenience, we also copied the revised DISCUSSION below:

2) the interpretation of MR results should come from combined evidence, for instance, combining univariable, multivariable, and mediation MR together, or combing MR with other types of evidence (fine mapping or raw-data analysis) together. Need to discuss how to avoid overinterpretation of MR results alone.

Response: Thank you for your valuable suggestion regarding the interpretation of MR results. We agree that MR findings should not be interpreted in isolation.
Per your suggestion, in the revised Discussion, we have emphasized the importance of integrating evidence from multiple MR approaches (such as univariable, multivariable, and mediation MR) as well as from complementary sources (including fine mapping, colocalization, and raw data analyses) to strengthen causal inference and avoid overinterpretation based solely on MR results. This addition aims to encourage more robust and cautious interpretation of MR analyses conducted using our platform.
Furthermore, we have included prominent reminders on our website regarding the methodological rigor, evidence integration, and known limitations of MR, to further guide users in conducting reliable and valid analyses (also see above picture).
For your convenience, we also copied the revised DISCUSSION below:

3) Limitation of 2-sample MR should be also discussed.

Response: Thank you for your valuable comment regarding the limitations of two-sample Mendelian randomization (2SMR). We agree that it is important to acknowledge the key assumptions and potential limitations of this approach.
Per your advice, In the revised Discussion, we have added a dedicated paragraph outlining the main limitations of 2SMR, including issues related to sample overlap, heterogeneity between datasets, instrument strength, and potential bias due to horizontal pleiotropy. We believe this addition will provide a more balanced and comprehensive perspective for users of our platform.
Furthermore, we have included prominent reminders on our website regarding the methodological rigor, evidence integration, and known limitations of MR, to further guide users in conducting reliable and valid analyses (also see above picture).
For your convenience, we also copied the revised DISCUSSION below:

Reviewer #2

Review of the Manuscript: "MRanalysis: A Comprehensive Online Platform for Integrated, Multi-Method Mendelian Randomization and Associated Post-GWAS Analyses"
This manuscript introduces MRanalysis, a web-based platform designed to facilitate Mendelian Randomization (MR) analyses and associated post-GWAS workflows, supported by a companion tool, GWASkit, for formatting and validating GWAS summary statistics. The authors aim to address persistent challenges in the MR research landscape, including inconsistent summary data formats, lack of standard analytical workflows, and the technical barrier posed by programming requirements.
The platform is clearly user-oriented, providing a graphical interface that simplifies the process of performing MR analyses without coding. Features such as flexible column mapping, a variety of MR methods, and downstream utilities like gene enrichment and visualization contribute to a positive user experience.
However, while the tool is well-positioned as a front-end solution for MR analyses, several critical limitations must be addressed before the manuscript can be considered for publication.

Response: We appreciate the reviewer's positive comments and recognition for our MRanalysis tool. We have carefully revised our manuscript and the webtool based on your valuable suggestions.

1. Lack of Colocalization Analysis
The absence of integrated genetic colocalization analysis represents a fundamental weakness of the platform. In MR, particularly when used for biomarker discovery and therapeutic target identification, colocalization is essential to validate whether the genetic signal driving the exposure-outcome association is shared between traits or arises from distinct, but nearby, loci in linkage disequilibrium. Without colocalization support (e.g., COLOC, eCAVIAR, or fastENLOC), MR findings are prone to false positives and cannot be reliably interpreted as evidence of causality. This is especially problematic in highly pleiotropic genomic regions. The current version of the platform thus risks encouraging misleading conclusions by presenting potentially spurious associations without necessary validation. Incorporating colocalization is not optional—it is a core requirement for robust causal inference.

Response: Thank you very much for your critical comment regarding the lack of integrated colocalization analysis within our platform. We fully agree that colocalization is essential for robust causal inference in Mendelian randomization (MR), particularly to distinguish whether the genetic association signals for exposure and outcome are driven by the same causal variant or by distinct, but nearby, loci in linkage disequilibrium. This is especially important for biomarker discovery and therapeutic target prioritization, as failure to account for colocalization can result in spurious associations and misleading conclusions.
In response to your feedback, we have now integrated four widely used colocalization methods into our platform: 1) COLOC.ABF: Bayesian framework estimating the probability that two traits share a causal variant. 2) COLOC.SUSIE: Utilizes the Sum of Single Effects (SuSiE) model for complex multi-SNP colocalization analysis. 3) COLOC.PWCoCo: Hierarchical clustering-based approach to identify shared causal

variants across traits. 4) COLOC.eCAVIAR: CAusal Variants Identification in Associated Regions for rigorous colocalization assessment. These tools allow users to assess whether the genetic association signals for exposure and outcome are likely due to the same underlying causal variant, thereby strengthening the causal inference from MR and facilitating more robust drug-target prioritization.

We have updated the manuscript, the webtool and the corresponding user documentation to reflect this important addition (https://mranalysis.cn/plugins/COLOC/). We appreciate your insightful suggestion, which has helped to significantly improve the scientific utility and translational relevance of our platform.

2. Lack of Integration with Drug Target and Clinical Trial Databases
One of the most compelling applications of MR is in identifying and prioritizing potential drug targets. However, MRanalysis currently lacks integration with widely used drug target and trial resources such as Open Targets, DrugBank, or ClinicalTrials.gov. This omission limits the utility of the tool in translational research and biomarker evaluation. Users should be able to trace MR findings to known or emerging drug targets and assess whether implicated genes are supported by clinical or pharmacological evidence.

Response: Thank you for highlighting the importance of integrating drug target and clinical trial databases for translational research. In the latest version of MRanalysis, we have addressed this limitation by incorporating direct links to widely used resources such as Open Targets, DrugBank, and ClinicalTrials.gov. For each prioritized gene, the platform now displays drug target annotations and provides convenient hyperlinks to relevant entries in these databases. This allows users to rapidly trace MR findings to known or emerging drug targets and access supporting clinical or pharmacological evidence.
We believe these enhancements substantially strengthen MRanalysis as a tool for biomarker evaluation and translational research.

The address of this application: https://mranalysis.cn/plugins/MAGMA/

3. Limited Support for Large Datasets
At present, the platform can only process relatively small GWAS summary files, which is a practical limitation given the size of modern GWAS meta-analyses. Support for large-scale data, potentially via cloud integration or offline batch processing, is necessary for broad adoption.

Response: GitHub: https://github.com/xingabao/MRanalysis
Docker Hub: https://hub.docker.com/r/xingabao/mranalysis

Thank you very much for highlighting the current limitation regarding the processing of large GWAS summary data. We agree that the ability to efficiently handle large-scale datasets is essential for the broad adoption of our platform, especially given the increasing size of modern GWAS meta-analyses.

To address this, we have made several significant enhancements:
a). Docker-based deployment. We now provide a Docker image that allows users to easily deploy MRanalysis on their own computational infrastructure. This enables seamless and efficient processing of large datasets without the restrictions of online applications, allowing users to leverage their own hardware resources. With Docker, the entire environment can be set up locally within 2 to 3 minutes, greatly reducing installation time and avoiding software version inconsistencies that previously required 3 to 4 hours of manual setup. Comprehensive installation and usage instructions are included in the user documentation.
b). Open-source and extensible. The complete MRanalysis codebase is fully open-sourced on GitHub, comprising over 60,000 lines of code. Beyond integrating numerous established packages and tools, we have also developed a wide range of proprietary features – including rs ID mapping, universal GWAS format conversion, fast VCF file parsing, and versatile data visualization modules. This comprehensive release enables transparency, reproducibility, and community-driven improvement.
c). Enhanced GWAS data handling. A new data format conversion application has been introduced, allowing users to extract and covert common GWAS data formats (such as VCF, TSV, and CSV) into forms suitable for downstream analysis. This greatly improves the flexibility and usability of the platform for diverse large-

scale GWAS summary datasets.
In future updates, we will continue to explore additional solutions, such as cloud-based processing and enhanced batch analysis, to further improve scalability. We sincerely appreciate your helpful suggestions, which have contributed to strengthening both the utility and accessibility of MRanalysis.

4. Performance Issues
Some computational modules, especially for multivariable MR, run slowly and may become impractical with larger datasets. Optimization of computational backends or asynchronous job handling would improve usability.

Response: Thank you for raising the important issue of computational performance, particularly regarding the speed of multivariable MR analyses on large datasets. We recognize that slow processing can hinder usability and limit the practical application of the platform as data sizes continue to grow.
To address this, we have implemented a series of optimizations in our computational workflow. Previously, analyses using VariantAnnotation and gwasglue to read local files could take 20 to 30 minutes to complete; with our recent improvements, processing speed has increased approximately tenfold, allowing most analyses to be completed within 2 to 3 minutes. In addition, the platform is now fully open source and supports local deployment via Docker, enabling users to utilize their own hardware for faster analysis of large-scale data (also see our response to comment #3).
Thank you again for your valuable feedback, which has been instrumental in guiding the ongoing development and optimization of our platform.

5. Gene Annotation Needs Improvement
In the current implementation, gene annotation results display only Gene IDs, which are not easily interpretable. Inclusion of gene symbols and functional annotations would enhance the accessibility and utility of the results.

Response: Thank you for your valuable suggestions regarding the interpretability of gene annotation results and the integration of translational resources. We fully agree that displaying only Gene IDs limits accessibility and practical utility for users. In response, we have significance enhanced the SNP-to-gene mapping and enrichment module in MRanalysis.
The annotation results now include not only gene symbols but also full gene names and functional annotations for each mapped gene, greatly facilitating immediate biological interpretation. To further support translational research, we have addressed the previous lack of integration with drug target and clinical trial databases. The updated application now provides direct links and annotation retrieval from widely used resources such as Open Targets, DrugBank, and ClinicalTrials.gov (also see our response to comment #2). For each gene, users can conveniently access relevant drug target information and ongoing or completed clinical trials via embedded hyperlinks to the official database entries.
These improvements make the results more user-friendly and valuable for downstream biological interpretation, enabling users to rapidly trace MR findings to known or emerging drug targets, evaluate pharmacological or clinical evidence, and prioritize genes for further functional or therapeutic investigation.
We appreciate your insightful feedback, which has significantly contributed to enhancing the usability and scientific value of our platform.

The address of this application: https://mranalysis.cn/plugins/MAGMA/

6. The website design is acceptable, but the domain https://mranalysis.cn/ is considered insecure in several foreign institutions' networks. The authors should try to fix the issue.

Response:nThank you for bringing up the issue regarding website security and accessibility from certain institutional networks.
Per your suggestion, we have performed the following revisions:
(1) We have upgraded to a more secure and widely recognized SSL certificate. After multiple rounds of testing, we have not encountered any further warnings or issues indicating that the website is insecure.

(2) Additionally, to fully address any potential security concerns, we have open-sourced our application and now provide a Docker image for local deployment. This allows users to install and run MRanalysis entirely on their own infrastructure, eliminating any risks associated with web access. The open-source nature of the code also ensures full transparency, so users can verify that there are no hidden or insecure components.
We hope these measures will resolve the security concerns and further enhance user trust in our platform.
Thank you again for your valuable feedback.

Cookie Preference Center

We use cookies which are necessary to make our site work. We may also use additional cookies to analyze, improve and personalize our content and your digital experience. For more information, see our Cookie Policy.

You may choose not to allow some types of cookies. However, blocking some types may impact your experience of our site and the services we are able to offer. See the different category headings below to find out more or change your settings.

Allow all

Manage Consent Preferences

Strictly Necessary Cookies

Always active

These cookies are necessary for the website to function and cannot be switched off in our systems. They are usually only set in response to actions made by you which amount to a request for services, such as setting your privacy preferences, logging in or filling in forms. You can set your browser to block or alert you about these cookies, but some parts of the site will not then work.

Functional Cookies

These cookies enable the website to provide enhanced functionality and personalisation. They may be set by us or by third party providers whose services we

have added to our pages. If you do not allow these cookies then some or all of these services may not function properly.

Performance Cookies

These cookies allow us to count visits and traffic sources so we can measure and improve the performance of our site. They help us to know which pages are the most and least popular and see how visitors move around the site.

Targeting Cookies

These cookies may be set through our site by our advertising partners. They may be used by those companies to build a profile of your interests and show you relevant adverts on other sites. If you do not allow these cookies, you will experience less targeted advertising.

Cookie List

Clear

Apply Cancel
Consent Leg.Interest

Confirm my choices

Powered by onetrust
