# Supplementary material for: MRanalysis: a comprehensive online platform for integrated, multimethod Mendelian randomization and associated post-GWAS analyses
Source: Gigascience. 2025 Oct 22;14:giaf131. doi: 10.1093/gigascience/giaf131 (PMC12616851; doi:10.1093/gigascience/giaf131)
Supplement: giaf131_Reviewer_1_Report_Original_Submission — Hongsheng Gui -- 5/18/2025 [file giaf131_reviewer_1_report_original_submission.pdf]

Reviewer Report

Title: MRanalysis: A Comprehensive Online Platform for Integrated, Multi-Method Mendelian Randomization and Associated Post-GWAS Analyses

Version: Original Submission **Date:** 5/18/2025

Reviewer name: Hongsheng Gui

Reviewer Comments to Author:

In this study, Xing et al. presented an online platform for performing GWAS data preprocessing, Mendelian Randomization (MR) analyses, and post-GWAS annotation. Multiple standard tools have been implemented to ease MR steps and facilitate more robust genetic analyses to infer complex relationships between complex traits. While I see the value of developing integrated platform for MR users, I also have several major concerns regarding to its novelty, framework, and interpretation.

1. While GWASkit is a self-developed tool, all those 3 types of MR analyses and their R packages were previously developed. The novelty level for method part is not high. I am a little concerned about the copyright for implementing those existing tools and suggest adding some acknowledgement or evidence of agreement from original developers.
2. About mediation MR: the justification of defining exposure, mediator, and outcome is not clear. Without individual-level data, how valid is the estimated mediation proportion using sum data? In other words, does lonely-cigarettes-MDD result in case VI align well with traditional one-sample mediation result?
3. About post-MR: besides enrichment and visualization, other post-GWAS analysis (e.g., colocalization) that is supplementary to MR should be incorporated, as this will be essential for drug-target selection.
4. Power and sample size calculation: I would suggest adding another case example for showing sample size needed for 3 types of MR analysis.
5. About Discussion:

1)though it is important to ease MR analysis for all researchers, there is also a caution to avoid abusing MR for randomly selected cross-trait analysis. Need to discuss this when designing MR analysis.

2)the interpretation of MR results should come from combined evidence, for instance, combining univariable, multivariable, and mediation MR together, or combing MR with other types of evidence (fine mapping or raw-data analysis) together. Need to discuss how to avoid overinterpretation of MR results alone.

3)Limitation of 2-sample MR should be also discussed.

Methods

Are the methods appropriate to the aims of the study, are they well described, and are necessary controls included? Choose an item.

Conclusions

Are the conclusions adequately supported by the data shown? Choose an item.

Reporting Standards

Does the manuscript adhere to the journal's guidelines on minimum standards of reporting? Choose an item.

Choose an item.

Statistics

Are you able to assess all statistics in the manuscript, including the appropriateness of statistical tests used? Choose an item.

Quality of Written English

Please indicate the quality of language in the manuscript: Choose an item.

Declaration of Competing Interests

Please complete a declaration of competing interests, considering the following questions:

- Have you in the past five years received reimbursements, fees, funding, or salary from an organisation that may in any way gain or lose financially from the publication of this manuscript, either now or in the future?
- Do you hold any stocks or shares in an organisation that may in any way gain or lose financially from the publication of this manuscript, either now or in the future?
- Do you hold or are you currently applying for any patents relating to the content of the manuscript?
- Have you received reimbursements, fees, funding, or salary from an organization that holds or has applied for patents relating to the content of the manuscript?
- Do you have any other financial competing interests?
- Do you have any non-financial competing interests in relation to this paper?

If you can answer no to all of the above, write 'I declare that I have no competing interests' below. If your reply is yes to any, please give details below.

I declare that I have no competing interests.

I agree to the open peer review policy of the journal. I understand that my name will be included on my report to the authors and, if the manuscript is accepted for publication, my named report including any attachments I upload will be posted on the website along with the authors' responses. I agree for my report to be made available under an Open Access Creative Commons CC-BY license (http://creativecommons.org/licenses/by/4.0/). I understand that any comments which I do not wish to

be included in my named report can be included as confidential comments to the editors, which will not be published.

Choose an item.

To further support our reviewers, we have joined with Publons, where you can gain additional credit to further highlight your hard work (see: https://publons.com/journal/530/gigascience). On publication of this paper, your review will be automatically added to Publons, you can then choose whether or not to claim your Publons credit. I understand this statement.

Yes Choose an item.
