# Supplementary material for: MRanalysis: a comprehensive online platform for integrated, multimethod Mendelian randomization and associated post-GWAS analyses
Source: Gigascience. 2025 Oct 22;14:giaf131. doi: 10.1093/gigascience/giaf131 (PMC12616851; doi:10.1093/gigascience/giaf131)
Supplement: giaf131_Reviewer_2_Report_Original_Submission — Xia Shen -- 5/21/2025 [file giaf131_reviewer_2_report_original_submission.pdf]

Reviewer Report

Title: MRanalysis: A Comprehensive Online Platform for Integrated, Multi-Method Mendelian Randomization and Associated Post-GWAS Analyses

Version: Original Submission **Date:** 5/21/2025

Reviewer name: Xia Shen

Reviewer Comments to Author:

Review of the Manuscript: "MRanalysis: A Comprehensive Online Platform for Integrated, Multi-Method Mendelian Randomization and Associated Post-GWAS Analyses"

This manuscript introduces MRanalysis, a web-based platform designed to facilitate Mendelian Randomization (MR) analyses and associated post-GWAS workflows, supported by a companion tool, GWASkit, for formatting and validating GWAS summary statistics. The authors aim to address persistent challenges in the MR research landscape, including inconsistent summary data formats, lack of standard analytical workflows, and the technical barrier posed by programming requirements.

The platform is clearly user-oriented, providing a graphical interface that simplifies the process of performing MR analyses without coding. Features such as flexible column mapping, a variety of MR methods, and downstream utilities like gene enrichment and visualization contribute to a positive user experience.

However, while the tool is well-positioned as a front-end solution for MR analyses, several critical limitations must be addressed before the manuscript can be considered for publication.

Major Concerns:

1. Lack of Colocalization Analysis

The absence of integrated genetic colocalization analysis represents a fundamental weakness of the platform. In MR, particularly when used for biomarker discovery and therapeutic target identification, colocalization is essential to validate whether the genetic signal driving the exposure-outcome association is shared between traits or arises from distinct, but nearby, loci in linkage disequilibrium. Without colocalization support (e.g., COLOC, eCAVIAR, or fastENLOC), MR findings are prone to false positives and cannot be reliably interpreted as evidence of causality. This is especially problematic in highly pleiotropic genomic regions. The current version of the platform thus risks encouraging misleading conclusions by presenting potentially spurious associations without necessary validation. Incorporating colocalization is not optionalâ€"it is a core requirement for robust causal inference.

2. Lack of Integration with Drug Target and Clinical Trial Databases

One of the most compelling applications of MR is in identifying and prioritizing potential drug targets. However, MRanalysis currently lacks integration with widely used drug target and trial resources such as Open Targets, DrugBank, or ClinicalTrials.gov. This omission limits the utility of the tool in translational research and biomarker evaluation. Users should be able to trace MR findings to known or emerging drug targets and assess whether implicated genes are supported by clinical or pharmacological evidence.

3. Limited Support for Large Datasets

At present, the platform can only process relatively small GWAS summary files, which is a practical limitation given the size of modern GWAS meta-analyses. Support for large-scale data, potentially via cloud integration or offline batch processing, is necessary for broad adoption.
4. Performance Issues
Some computational modules, especially for multivariable MR, run slowly and may become impractical with larger datasets. Optimization of computational backends or asynchronous job handling would improve usability.
5. Gene Annotation Needs Improvement
In the current implementation, gene annotation results display only Gene IDs, which are not easily interpretable. Inclusion of gene symbols and functional annotations would enhance the accessibility and utility of the results.
6. The website design is acceptable, but the domain https://mranalysis.cn/ is considered insecure in several foreign institutions' networks. The authors should try to fix the issue.

Methods

Are the methods appropriate to the aims of the study, are they well described, and are necessary controls included? Choose an item.

Conclusions

Are the conclusions adequately supported by the data shown? Choose an item.

Reporting Standards

Does the manuscript adhere to the journal's guidelines on minimum standards of reporting? Choose an item.

Choose an item.

Statistics

Are you able to assess all statistics in the manuscript, including the appropriateness of statistical tests used? Choose an item.

Quality of Written English

Please indicate the quality of language in the manuscript: Choose an item.

Declaration of Competing Interests

Please complete a declaration of competing interests, considering the following questions:

- Have you in the past five years received reimbursements, fees, funding, or salary from an organisation that may in any way gain or lose financially from the publication of this manuscript, either now or in the future?
- Do you hold any stocks or shares in an organisation that may in any way gain or lose financially from the publication of this manuscript, either now or in the future?
- Do you hold or are you currently applying for any patents relating to the content of the manuscript?
- Have you received reimbursements, fees, funding, or salary from an organization that holds or has applied for patents relating to the content of the manuscript?
- Do you have any other financial competing interests?
- Do you have any non-financial competing interests in relation to this paper?

If you can answer no to all of the above, write 'I declare that I have no competing interests' below. If your reply is yes to any, please give details below.

I declare that I have no competing interests.

I agree to the open peer review policy of the journal. I understand that my name will be included on my report to the authors and, if the manuscript is accepted for publication, my named report including any attachments I upload will be posted on the website along with the authors' responses. I agree for my report to be made available under an Open Access Creative Commons CC-BY license (http://creativecommons.org/licenses/by/4.0/). I understand that any comments which I do not wish to be included in my named report can be included as confidential comments to the editors, which will not be published.

Choose an item.

To further support our reviewers, we have joined with Publons, where you can gain additional credit to further highlight your hard work (see: https://publons.com/journal/530/gigascience). On publication of this paper, your review will be automatically added to Publons, you can then choose whether or not to claim your Publons credit. I understand this statement.

Yes Choose an item.
